# Identifying eating behaviour phenotype cut-off scores for childhood obesity: an individual participant data meta-analysis using the Child Eating Behaviour Questionnaire (CEBQ)

**DOI:** 10.1186/s12966-026-01951-8

**Published:** 2026-07-25

**Authors:** Laura Kudlek, Andrea D. Smith, Cara F. Ruggiero, Lisa Pennells, Alice R. Kininmonth, Silje Steinsbekk, Pauline W. Jansen, Amy L. Ahern, Elaine K. McCarthy, Amy Gross, Victor Viana, Arend W. van Deutekom, John C. Spence, Jennifer Orlet Fisher, Jacqueline Hayes, Emma Haycraft, Charlotte A. Hardman, Patricia P. Silveira, Jennifer A. Emond, Aino-Maija Eloranta, Darlise R. dos Passos Gomes, Julie C. Lumeng, Matthew B. Cross, Susan Carnell, Alison Fildes, Clare Llewellyn

**Affiliations:** 1 Institute of Metabolic Science Epidemiology, University of Cambridge, Cambridge, UK; 2 Centre for Preventive Medicine and Digital Health, Medical Faculty Mannheim, University of Heidelberg, Heidelberg, Germany; 3 Department of Health and Primary Care, University of Cambridge, Cambridge, UK; 4 School of Food Science and Nutrition, University of Leeds, Leeds, UK; 5 Leeds Institute for Data Analytics, University of Leeds, Leeds, UK; 6 Department of Psychology, Norwegian University of Science and Technology, Høgskoleringen, Norway; 7 Department of Psychology, Education and Child Studies, Erasmus University Rotterdam, Rotterdam, Netherlands; 8 School of Food and Nutritional Sciences, University College Cork, Cork, Ireland; 9 Department of Pediatrics, University of Minnesota, Minneapolis, MN USA; 10 Faculty of Food Sciences and Nutrition, University of Porto, Porto, Portugal; 11 Department of Paediatrics, Division of Paediatric Cardiology Erasmus MC-Sophia Children’s Hospital Rotterdam, Rotterdam,, Netherlands; 12 Faculty of Kinesiology, Sport, & Recreation, University of Alberta, Edmonton, AB Canada; 13 Department of Social and Behavioral Sciences, College of Public Health, Temple University, Philadelphia, PA USA; 14 Alpert Medical School of Brown University and The Miriam Hospital, Providence, RI USA; 15 School of Sport, Exercise and Health Sciences, Loughborough University, Loughborough, UK; 16 Department of Psychology, University of Liverpool, Liverpool, UK; 17 Department of Psychiatry, McGill University, Montréal, Québec Canada; 18 Department of Biomedical Data Science, Geisel School of Medicine at Dartmouth College, Lebanon, NH USA; 19 Institute of Public Health and Clinical Nutrition, School of Medicine, University of Eastern Finland, Kuopio, Finland; 20 Department of Nutrition, Federal University of Pelotas, Pelotas, RS Brazil; 21 Department of Pediatrics, University of Michigan, Ann Arbor, MI USA; 22 USDA/ARS Children’s Nutrition Research Center, Baylor College of Medicine, Bates, Houston, TX 1100 USA; 23 Department of Psychiatry and Behavioral Sciences, Division of Child and Adolescent Psychiatry, Johns Hopkins University School of Medicine, Baltimore, MD USA; 24 School of Psychology, University of Leeds, Leeds, UK; 25 Department of Behavioural Science and Health, University College London, London, UK

**Keywords:** Child eating behaviour questionnaire (CEBQ), Cut-off scores, Childhood obesity, Behavioural obesity phenotypes

## Abstract

**Background:**

Weight management interventions targeting appetitive traits show promise in treating childhood obesity, yet a tool to identify appetitive phenotypes for personalised treatment is missing. The Child Eating Behaviour Questionnaire (CEBQ) is a widely used measure of eight parent-reported appetitive traits associated with childhood obesity. We aimed to explore the utility of the CEBQ for phenotyping childhood obesity and derive cut-off scores to support clinical decision-making.

**Methods:**

This individual participant data meta-analysis combined cross-sectional data from 17 international studies (*N* = 15,273) on CEBQ scores and Body Mass Index Standard Deviation Scores from children (2–18 years). The potential of CEBQ subscales (Food Responsiveness, Enjoyment of Food, Emotional Over-eating, Desire to Drink, Satiety Responsiveness, Slowness in Eating, Emotional Under-eating, Food Fussiness) to discriminate weight status was explored in random-effect meta-analyses of Area Under the Curve (AUC) metrics. We explored heterogeneity across studies and age- and sex- specific subgroups. Cut-off scores were derived within subgroups using (a) the Youden’s Index and (b) the point maximising specificity after a minimum sensitivity of 60%. We meta-analysed study-specific performance with the selected cut-offs to describe heterogeneity across studies.

**Results:**

AUCs for CEBQ subscales ranged from 0.60 to 0.71. The magnitude and shape of associations between CEBQ scores and obesity varied by age and sex. Cut-off performance was generally better in middle- than early childhood. The best performing cut-off score of 2.6 was observed for females aged 7–12 years for Food Responsiveness (Sensitivity: 0.81 [95%CI 0.77, 0.85]; Specificity: 0.85 [95%CI 0.82, 0.88]). High heterogeneity in cut-off performance across studies and wide prediction intervals suggest the need to further investigate external validity.

**Conclusions:**

We provide the first empirically derived CEBQ subscale cut-offs to phenotype children with obesity based on weight-related eating behaviours. Cut-offs could support personalised treatment in clinical and research contexts.

**Funding:**

MC_UU_00006/6 and MC_UU_00006/5.

**Supplementary Information:**

The online version contains supplementary material available at https://doi.org/10.1186/s12966-026-01951-8.

## Background

Childhood obesity is one of the greatest public health challenges of our time. Despite large investment in prevention and treatment strategies, its prevalence remains high. Globally, 35 million children are currently living with overweight or obesity, [1] putting them at greater risk of physical and mental comorbidities concurrently and in later life [2]. The economic impact of childhood obesity is predicted to double by 2035, reaching 208 million USD annually for boys and 175 USD million annually for girls [3]. While behavioural obesity treatments can be effective for some children, they do not work equally well for every child [4]. Identifying ways to improve treatment approaches and address the individual variability in outcomes is therefore essential to reduce the health burden, as well as the substantial economic and societal costs of childhood obesity.

To improve obesity treatment, researchers and clinicians have been advocating for the development of precision medicine approaches, whereby different treatments are matched to individuals based on personal characteristics, including obesity phenotypes based on *‘appetitive’-* or *‘eating behaviour’* traits [5]. A well-established and widely used tool for assessing eating behaviour is the Child Eating Behaviour Questionnaire (CEBQ), which has been translated into more than 20 languages worldwide and captures eight distinct traits: four food approach traits, indicating a more avid appetite (i.e. Food Responsiveness (FR), Enjoyment of Food (EF), Emotional over-eating (EOE), Desire to Drink (DD)), and four food avoidant traits, reflecting a less avid appetite (i.e. Satiety Responsiveness (SR), Slowness in eating (SE), Emotional under-eating (EUE), Food Fussiness (FF)) [6]. These traits reflect partially heritable, partially learned individual differences in eating behaviour in response to a range of internal and external cues [7].

A growing body of evidence indicates that appetitive traits play a key role in both the development and treatment of childhood obesity. For example, a large systematic review and meta-analysis found consistent associations between the CEBQ traits and childhood obesity measures [8]. Importantly, appetitive traits have been linked to differences in weight loss outcomes following treatment in children, [9] supporting their potential for guiding precision treatment approaches. Moreover, a recent systematic review and meta-analysis suggests many of these traits may be modifiable through weight management interventions, [10] highlighting their value as intervention targets. Interventions designed to address specific appetitive traits in children, such as satiety responsiveness and food responsiveness, have already been developed and show promising results, underscoring recent advancements in the field [11–14]. For example, interventions targeting satiety responsiveness used mindfulness and self-monitoring exercises to increase awareness of internal hunger and fulness, whereas interventions targeting food responsiveness employed cue-exposure and behavioural reinforcement exercises to change children’s eating patterns [11–14]. Despite this growing evidence, there is currently no standardised method for using the CEBQ, or a similar instrument, to identify children that may benefit most from such different types of eating behaviour-guided obesity treatment. As a result, previous research and practice have not incorporated appetitive trait assessments in participant selection for targeted interventions.

The aim of this study was to explore the potential utility of the CEBQ as a standardised tool to identify appetitive traits associated with obesity in paediatric populations and inform future research and clinical decision-making in precision medicine approaches to childhood obesity treatment. Specifically, we conducted an Individual Participant Data (IPD) meta-analysis, a method where raw data from systematically identified studies are combined and re-analysed, allowing for flexible and in-depth analyses independent of the original study aims and providing access to a large, diverse sample. Using this approach, we derived cut-off scores for the CEBQ-measured appetitive traits that can serve as reference points to phenotype a child’s obesity and may lay the groundwork for future research and clinical practice to provide tailored treatment based on eating behaviour traits. We further explored variability in the performance of these cut-off scores across included cohorts to evaluate their generalisability across different contexts.

## Methods

This IPD meta-analysis was retrospectively registered on PROSPERO (CRD42024592027). The analysis plan was pre-registered on the Open Science Framework (https://doi.org/10.17605/OSF.IO/B49TU). Reporting adheres to the Preferred Reporting Items for Systematic Reviews and Meta-Analyses IPD extension (PRISMA-IPD) [15].

### Study identification and selection

**Fig. 1:**
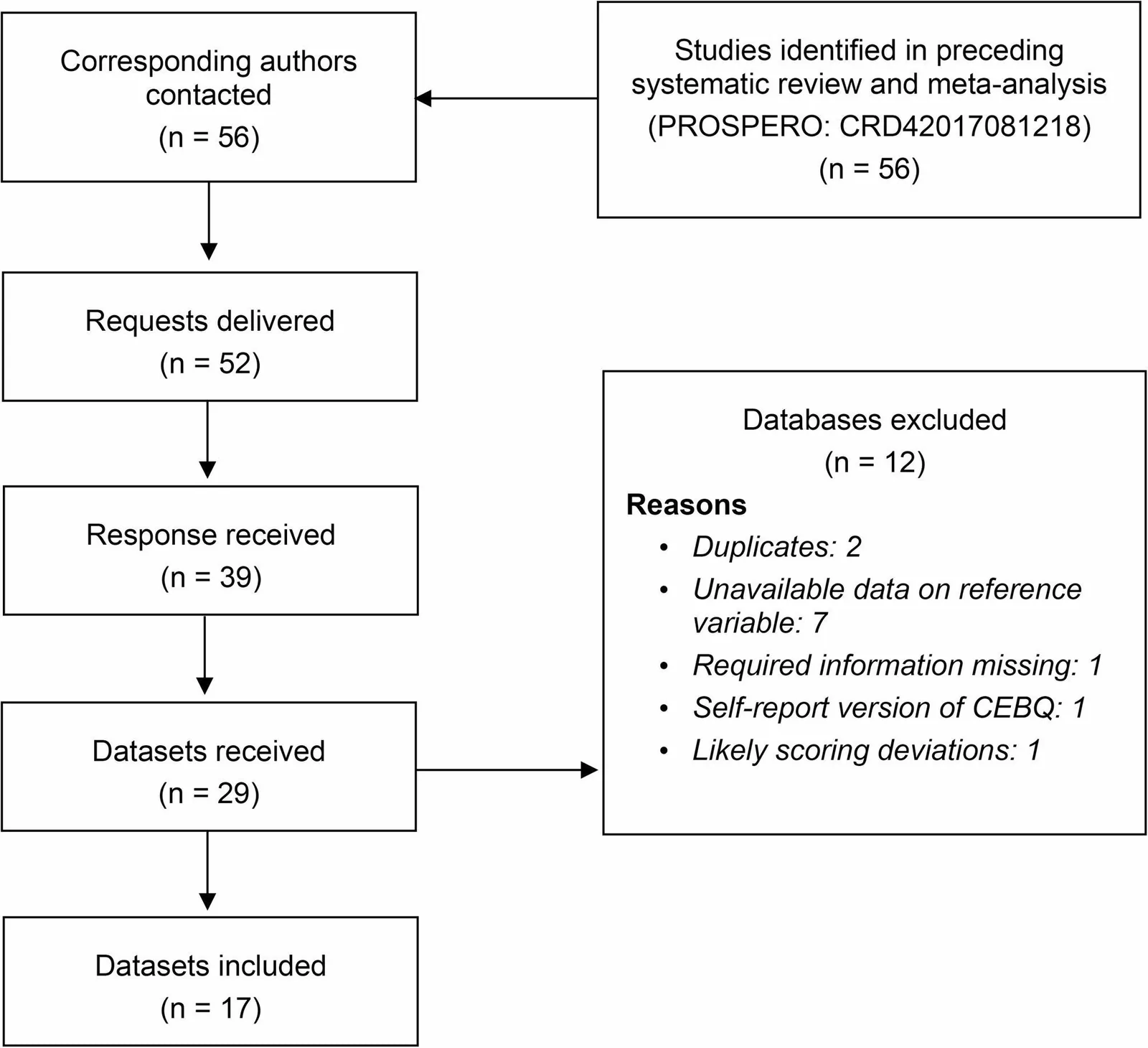
Flowchart of the data collection process

The identification of eligible datasets was based on a previously published systematic review and meta-analysis [8]. This review identified observational studies on children that reported at least one association between a CEBQ subscale and any measure of adiposity. The corresponding authors of included studies were contacted by e-mail to request raw data on CEBQ scores, age, sex, height and weight. Data requests were followed up with at least two reminders. Eligible IPD datasets contained data allowing for the computation of at least one original CEBQ subscale and Body Mass Index Standard Deviation Scores (BMI-SDS) calculated using World Health Organization [WHO] reference standards [16]. Datasets were excluded if no response was received after three contacts or irreversible modifications were applied to the CEBQ (e.g. child self-report format, reduced number of items) (Fig. 1; Supplementary Material Section "Results").

### Data extraction, risk of bias assessment, harmonisation and checking

Data on country, sample and language of included studies were extracted by three independent reviewers (AS, AK, LK). Risk of bias was assessed in duplicate (CR, LK) using an adapted Newcastle-Ottawa-Scale (Supplementary Material Sect. 5.1) [17].

IPD were harmonised by transforming height and weight into metric units, age into age in months, applying a uniform coding scheme for sex, and re-coding all CEBQ subscales to match the original scoring approach. Authors were contacted for additional information (e.g. missing labels, or unknown reversed status of items) where necessary (*n* = 14).

The scoring of the CEBQ was checked by re-calculating subscale scores when both item and subscale scores were reported (*n* = 13). This led to the re-calculation of subscale scores in datasets where single items had been reversed or allocated differently (*n* = 2).

### Measures

#### Index test (appetitive traits)

The CEBQ is a 35-item parent-reported measure of eight appetitive traits, including four food-approach and four food-avoidant traits [6]. Each item is assessed using a five-point Likert scale (1 = “never” to 5 = “always”) (see Supplementary Material Sect. 1.0). Food-approach traits describing a larger appetite include food responsiveness (FR, 5 items, e.g. “my child is always asking for food”), emotional over-eating (EOE, 4 items, e.g. “my child eats more when worried”), enjoyment of food (EF, 4 items, e.g. “my child loves food”) and desire to drink (DD, 3 items, e.g. “my child is always asking for a drink”). Food-avoidant traits indicating a lower appetite include satiety responsiveness (SR, 5 items, e.g. “my child gets full up easily”), slowness in eating (SE, 4 items, e.g. “my child eats slowly”), emotional under-eating (EUE, 4 items, e.g. “my child eats less when upset”) and food fussiness (FF, 6 items, e.g. “my child is difficult to please with meals”). CEBQ subscale scores are calculated as the mean of the items corresponding to each scale, with higher scores indicating a greater expression of the behaviours relating to the underlying appetitive trait.

The CEBQ has good psychometric properties, including a robust factor structure, [18] high test-retest reliability, [6] and good criterion validity when compared to behavioural measures assessed in the laboratory or observed in naturalistic settings, such as caloric compensation, eating in the absence of hunger, and energy intake [19]. It has been examined in several international populations, supporting its cross-cultural validity [18, 20–22].

#### Reference test (weight status)

BMI-SDS were used to generate a uniform reference variable for weight status across all data sources. BMI-SDS were calculated based on the WHO 2006 reference group of 0- to 5-year-olds, [23] and the WHO 2007 reference groups of 5- to 19-year-olds, [16] and were dichotomised (*“no obesity”*, i.e. BMI-SDS ≤ 2 vs. *“obesity”*, i.e. BMI-SDS *> 2*) based on the WHO cut-offs for weight classification [24]. BMI-SDS values equal to or smaller than − 3 and equal to or larger than + 8 were excluded, as they were considered to be Biologically Implausible Values (BIV), most likely resulting from errors in measurement or data entry [25].

### Statistical analyses

Before deriving cut-off scores on CEBQ subscales, we explored the obtained dataset through multiple approaches. Firstly, we visualised the shape of the association between CEBQ subscale scores and BMI-SDS category by plotting the odds ratios (ORs) for BMI-SDS category within quintiles or categories of CEBQ subscale scores against the mean value within each category using the Stata “mvshape” command [26]. This process involves pooling of ORs across studies using random effects meta-analysis and construction of 95% confidence intervals (CIs) using theory based on ‘floating absolute risks’ with standard errors estimated for each group (including the reference group) that corresponds to the amount of information underlying each group.

Next, we visually inspected CEBQ score distribution in BMI-SDS categories across cohorts in Ridgeline plots. These were produced using ggplot2 and ggridges in R [27, 28]. Then, we explored heterogeneity in CEBQ subscale performance by performing random-effects meta-analyses of the Area Under the Curve (AUC) values for each CEBQ subscale, adjusting for age and sex, and inspected I-squared (I^2^) to quantify the proportion of variability attributable to between-study heterogeneity. To give further insight into potential sources of heterogeneity, we then conducted meta-regressions entering pre-specified study characteristics as moderators. These included:

- Risk of bias (Cohorts classified as low risk vs. some concerns).
- Language (CEBQ administered in English vs. non-English).
- Sampling Type (Community sample (i.e. no eligibility criteria regarding weight or appetite) vs. Birth cohort samples (i.e. children were recruited from birth, including twin cohorts) vs. Clinical sample (i.e. at least some children were recruited from a clinic or eligibility criteria specified overweight and/or problematic appetite).
- Standard deviation of CEBQ subscale.
- Percentage of children with BMI-SDS > 2.

All pre-specified study-level moderators were entered simultaneously into a single meta-regression model for each CEBQ subscale. Results from such explorations were used to inform sensitivity analyses. All meta-analyses and meta-regression were performed using the metafor package in R [29].

To derive covariate-specific cut-off scores on all CEBQ subscales corresponding to an “optimal” discrimination of weight status, we determined the Youden’s Index, corresponding to the cut-off score with the highest sum of sensitivity and specificity [30]. Given an inclusive approach whereby higher sensitivity is prioritised in clinical practice (i.e. it is considered better to offer support to a child who may not need it than to miss one who does), we also derived cut-off scores maximising specificity after a minimum sensitivity level of 60% as a secondary approach. Cut-off scores were derived in age- and sex- strata including individuals from all cohorts. Age was categorised into three groups - early childhood (≥ 2 to < 7 years), middle childhood (≥ 7 to < 12 years) and late childhood (≥ 12 to ≤ 18 years) - to align with broadly recognised developmental stages and transitions in schooling. Age groups were pre-defined by the research team. Sex was treated as a binary variable (male vs. female). Cut-off scores were derived using the OptimalCutpoint Package in R [31].

To then provide an estimation of the variability of cut-off score performance across individual cohorts, we applied the derived cut-off scores in each cohort individually and synthesised sensitivity and specificity in a random-effects meta-analysis.

Analyses were conducted in Stata v.17 [32] and R v.4.1.2 [33].

#### Sensitivity analyses

Subsets of cohorts to be examined in sensitivity analyses were decided based on visual inspection of ridgeline plots, Receiver Operating Curves (ROCs), forest plots, and results from meta-regressions.

## Results

### Sample characteristics

We identified 56 studies during the preceding review [8] and contacted corresponding authors for additional participation in the present study. Datasets from 29 studies were received, of which 17 were eligible for inclusion. A total of 15,273 observations after excluding biologically implausible values (*n* = 123) and missing BMI-SDS values (*n* = 1889) were retained (see Supplementary Table SM 7 − 1). Figure 1 displays the data collection process. Supplementary Table SM 6 − 1 displays a summary of sample characteristics of individual data sources and the total sample. Average CEBQ subscale scores in each cohort are displayed in Supplementary Table SM 8 − 1.

Children across all cohorts were on average 7.21 (SD 2.83) years old and had a mean BMI-SDS of 0.55 (SD 1.44). Roughly half of children were female (50.46%), and 14.14% were classified as living with obesity. Most studies were conducted in the USA (*n* = 6), [34–39] followed by the United Kingdom (*n* = 3), [40–42] the Netherlands (*n* = 2), [43, 44] Canada (*n* = 2), [45, 46] Brazil (*n* = 1), [47] Finland (*n* = 1), [48] Ireland (*n* = 1) [49] and Portugal (*n* = 1) [50]. The CEBQ was mostly administered in English (*n* = 11), [34, 36–42, 45, 46, 49] and the studies were mostly classified as low risk of bias (*n* = 11) [35–37, 39, 42, 44–49]. Ten of the included studies were considered to be community samples, [35, 36, 38–41, 43, 46–48] four were birth cohort samples, [42, 44, 45, 49] and three were clinical samples [34, 37, 50].

### Exploration of age- and sex- specific patterns in the shape of associations between CEBQ subscales and obesity

Supplement S16 illustrates the shape of association between CEBQ subscale scores and BMI-SDS category across age-and sex- specific subgroups. Due to the low number of observations for children older than 12 years, plots are restricted to early- and middle childhood age groups. Higher scores on food approach traits (FR, EOE, EF, DD) appeared to be associated with a higher likelihood of obesity, whereas higher scores on food avoidant traits (SR, SE, FF, EUE) tended to correspond to a lower likelihood, though the strength and shape of these associations varied across CEBQ traits and age- and sex- groups. Stronger associations were often observed in middle childhood (FR, EOE, DD, SR). Only EF showed a comparatively stronger association in early childhood, particularly among males. Weak or negligible associations were observed for EOE in males in early childhood, for DD in males across both age groups, and for FF and EUE across all subgroups.

### Exploration of overall CEBQ subscale performance across cohorts

Table 1 displays the overall age- and sex- adjusted AUCs resulting from synthesising AUCs in individual cohorts by random effects meta-analysis. The overall discriminatory abilities of CEBQ subscales ranged from 0.60 (FF and EUE) to 0.71 (FR), whereby an AUC above 0.70 is generally considered to indicate acceptable discriminatory ability [51]. High heterogeneity was observed for the traits EOE (I^2^ = 85), FR (I^2^ = 80), and EF (I^2^ = 70) (Table 1).Supplement S12.0 shows ridgeline plots of the distributions of CEBQ subscale scores in BMI-SDS groups across cohorts, with some variation across cohorts for all CEBQ subscales. In particular, Hayes et al. (2016) often appeared to have high overlap in score distributions between BMI-SDS groups in comparison to other cohorts for all CEBQ subscales. This is likely due to the clinical nature of the sampling type, and the low numbers of children with BMI-SDS lower than two (see Table SM 10 − 1). We deemed this likely to be unproblematic when included in the pooled sample for cut-off derivation and thus decided not to exclude the cohort for further analyses. No other cohorts appeared to be outliers in terms of their score distributions.

**Table 1:** Meta-analysis of AUCs results

CEBQ subscale	AUC (95%CI)	Tau^2^	I^2^	*N* Cohorts
FR	0.71 (0.67, 0.76)	0.0071	80.11	15
EOE	0.69 (0.63, 0.75)	0.0109	85.95	15
EF	0.66 (0.63, 0.70)	0.0029	70.32	16
DD	0.61 (0.58, 0.64)	0.0013	40.50	15
SR	0.67 (0.65, 0.70)	0.0003	17.02	15
SE	0.65 (0.62, 0.67)	0.0003	14.57	15
FF	0.60 (0.57, 0.62)	0.0002	9.91	15
EUE	0.60 (0.57, 0.63)	0.0009	32.55	15

*Abbreviations:* *CEBQ* Child Eating Behaviour Questionnaire, *FR* Food Responsiveness, *EOE* Emotional over-eating, *EF* Enjoyment of Food, *DD* Desire to Drink, *SR* Satiety Responsiveness, *SE* Slowness in Eating, *FF* Food Fussiness, *EUE* Emotional under-eating, *AUC* Area Under the Curve, *CI* Confidence Interval, *N* Number of

Supplementary Material 13.0 contains ROC curves displaying the discriminatory abilities of CEBQ subscale scores in individual cohorts. Forest plots depicting AUCs values for CEBQ subscale scores in individual cohorts are displayed in Supplementary Materials 14.0. ROC curves and forest plots equally presented some variation across cohorts, in particular for FR and EOE, corresponding to the high heterogeneity estimates in Table 1. We observed no systematic patterns of variation or outlying cohorts.

The impact of pre-specified study-level characteristics (i.e. risk of bias, language, sampling type, standard deviation of CEBQ subscale, proportion of participants classified as having obesity) on predictive performance (AUC) was explored using meta-regression (Supplementary Material 15.0). Results from the test of moderators indicated that collectively, study characteristics explained a significant portion of the variance in AUC estimates for FR (QM (df = 6) 70.38, *p* < 0.0001), EOE (QM (df = 6) = 16.69, *p* = 0.0105), EF (QM (df = 6) = 18.65, *p* = 0.0048), EUE (QM (df = 6) = 14.13, *p* = 0.0281), and DD (QM (df = 6) = 15.96, *p* = 0.0139). The residual heterogeneity, I^2^, in meta-regression models ranged from 0 (FR, DD, SR, SE, EUE) to 72.54% (EOE), with most I^2^ values closer to zero, representing a reduction in heterogeneity values (Table SM 15 − 1). Among the study characteristics examined, English language (SR, FR, EF) and risk of bias (FR, EOE) were most frequently identified to impact AUC estimates. Specifically, studies using an English CEBQ version and studies classified as low risk of bias were associated with lower AUCs. However, the number of cohorts was imbalanced across study characteristics, with some categories containing only very few cohorts, limiting the statistical power of analyses and interpretability of results. Further details on individual CEBQ subscale results can be found in Table SM 15 − 2 for meta-regression results and in Table 15 − 3 to 15 − 6 for subgroup analyses.

### Derivation of cut-off scores

Cut-off scores are provided in Table 2. No cut-off scores were derived for the subscales of FF and EUE, nor for male subgroups in DD, given the low overall AUC of these subscales (see Table 1) and the weak associations observed across age- and sex- specific subgroups, decreasing their utility for phenotyping purposes (see Figure SM 16 − 4, SM 16 − 7 and SM 16 − 8). Additionally, a low number of observations for children older than 12 years meant that no cut-off scores could be generated for the “late childhood” age group.

**Table 2:** Age- and sex-specific cut-off scores to identify weight status (no obesity ^a^ vs. obesity ^b^) as derived using Youden’s Index

	Early childhood^c^					Middle childhood^d^				
	*N*	AUC [95%CI]	Cut-off^e^	Se [95%CI]	Sp [95%CI]	*N*	AUC [95%CI]	Cut-off^e^	Se [95%CI]	Sp [95%CI]
FR										
Males	3893	0.68 [0.64, 0.72]	2.60	0.50 [0.44, 0.56]	0.82 [0.80, 0.83]	3556	0.87 [0.84, 0.89]	2.50	0.78 [0.73, 0.82]	0.81 [0.77, 0.84]
Females	3817	0.70 [0.66, 0.73]	2.60	0.53 [0.46, 0.59]	0.80 [0.79, 0.82]	3760	0.89 [0.86, 0.91]	2.60	0.81 [0.77, 0.85]	0.85 [0.82, 0.88]
EF										
Males	3893	0.69 [0.66, 0.72]	3.50	0.68 [0.63, 0.73]	0.61 [0.60, 0.63]	3556	0.57 [0.54, 0.59]	4.25	0.57 [0.53, 0.61]	0.54 [0.52, 0.56]
Females	3817	0.71 [0.68, 0.74]	3.67	0.69 [0.63, 0.74]	0.67 [0.65, 0.68]	3760	0.58 [0.55, 0.60]	4.33	0.47 [0.43, 0.51]	0.64 [0.62, 0.66]
EOE										
Males	3893	0.55 [0.52, 0.59]	2.50	0.20 [0.16, 0.25]	0.90 [0.89, 0.92]	3556	0.79 [0.76, 0.82]	2.25	0.68 [0.63, 0.72]	0.79 [0.75, 0.83]
Females	3817	0.61 [0.58, 0.65]	2.00	0.50 [0.44, 0.56]	0.67 [0.65, 0.70]	3760	0.79 [0.76, 0.82]	2.25	0.67 [0.63, 0.72]	0.78 [0.75, 0.82]
DD										
Females	3817	0.55 [0.52, 0.59]	2.33	0.78 [0.73, 0.83]	0.32 [0.30, 0.34]	3760	0.70 [0.67, 0.74]	2.33	0.69 [0.64, 0.73]	0.70 [0.66, 0.74]
SR										
Males	3893	0.54 [0.50, 0.57]	2.60	0.52 [0.46, 0.58]	0.56 [0.55, 0.58]	3556	0.78 [0.75, 0.81]	2.60	0.79 [0.74, 0.83]	0.64 [0.59, 0.68]
Females	3817	0.56 [0.53, 0.60]	2.60	0.51 [0.45, 0.57]	0.61 [0.59, 0.62]	3760	0.78 [0.76, 0.81]	2.60	0.79 [0.75, 0.83]	0.67 [0.62, 0.71]
SE										
Males	3893	0.56 [0.53, 0.60]	2.50	0.58 [0.53, 0.64]	0.52 [0.50, 0.54]	3556	0.68 [0.65, 0.72]	2.00	0.57 [0.52, 0.62]	0.70 [0.66, 0.74]
Females	3817	0.57 [0.53, 0.61]	2.75	0.68 [0.62, 0.73]	0.42 [0.40, 0.44]	3760	0.72 [0.69, 0.75]	2.75	0.85 [0.81, 0.88]	0.47 [0.42, 0.51]

*Abbreviations:CEBQ* Child Eating Behaviour Questionnaire, *FR* Food Responsiveness, *EF* Enjoyment of Food, *EOE* Emotional over-eating, *SR* Satiety Responsiveness, *N* Number of participants, *AUC* Area Under the Curve, *CI* Confidence Interval, *Se* Sensitivity, *Sp* Specificity

^a^Classified into the no obesity group if BMI SDS ≤ 2

^b^Classified into the obesity group if BMI SDS > 2

^c^Children aged ≥ 2 to < 7 years

^d^Children aged ≥ 7 to < 12 years

^e^positive classification follows a score ≤ cut-off for subscales SR and a score ≥ cut-off for subscales FR, EF, and EOE

Overall, cut-off scores ranged from 2.0 to 2.75 in food-avoidant subscales (SE) and from 2.0 to 4.3 in food-approach subscales (EOE and EF). Sensitivity ranged from 20% (EOE) to 81% (FR), and specificity from 32% (DD) to 90% (EOE) (Table 2). As higher scores on food-approach traits are associated with a *higher* risk of obesity, while higher scores on food-avoidant traits are associated with a *lower* risk, a positive classification into the “obesity” group follows from a score *above* the cut-off for food-approach CEBQ subscales and *below* the cut-off for food-avoidant CEBQ subscales.

To illustrate potential clinical utility, examples of cut-off scores reaching a sensitivity of ≥ 60% (i.e. correctly identifying at least 60% of children with obesity) and specificity of ≥ 50% (i.e. correctly identifying at least 50% of children without obesity) were 2.5 (Se = 78%, Sp = 81%) and 2.6 (Se = 81%, Sp = 85%) for males and females for FR in middle childhood; 3.5 (Se = 68%, Sp = 61%) and 3.67 (Se = 69%, Sp = 67%) for males and females for EF in early childhood; 2.25 for both males (Se = 68%, Sp = 79%) and females (Se = 67%, Sp = 78%) for EOE in middle childhood; 2.33 (Se = 69%, Sp = 70%) for females for DD in middle childhood; and 2.6 for both males (Se = 79%, Sp = 64%) and females (Se = 79%, Sp = 67%) for SR in middle childhood (Table 2).

While our primary method to derive cut-off scores used the Youdens Index, we also explored thresholds that prioritised higher sensitivity (minimum 60%) (see Supplementary Materials 17.0). These alternative cut-off scores came at the cost of substantially reduced specificity and may thus be better suited for broad screening purposes rather than for more precise phenotyping.

#### Sensitivity analyses

Given that no clear patterns of heterogeneity were identified through visual inspection of plots (SM 12.0, SM 13.0, SM 14.0), and meta-regression results were constrained by the low number of cohorts within each study characteristic category (SM 15.0), we were unable to identify meaningful subsets of cohorts for sensitivity analyses. Thus, we only derived cut-off scores using the full sample of cohorts.

#### Sensitivity and specificity of cut-off scores across cohorts

Results from meta-analyses synthesising the sensitivity and specificity values resulting from applying the cut-off scores derived in Sect. 3.3 (Table 2) to each individual cohort are displayed in Table 3. Heterogeneity was particularly high for specificity and for middle childhood. Prediction intervals, indicating the expected range of future values of sensitivity or specificity, were generally wide and often included the 50% mark for at least one of these metrics at a given cutoff. This reflects uncertainty in the classification performance of cut-off scores for future samples.

**Table 3:** Random-Effects Meta-Analysis of Cut-Off Score Performance (Youden’s Index) and Heterogeneity Across Cohorts

	Early childhood ^a^					Middle childhood ^b^				
	*N* Cohorts	Se [95%CI]; [95%PI] ^c^	I^2^	Sp [95%CI]; [95%PI] ^c^	I^2^	*N* Cohorts	Se [95%CI]; [95%PI] ^c^	I^2^	Sp [95%CI]; [95%PI] ^c^	I^2^
FR										
Males	12	0.5 [0.39, 0.6]; [0.2, 0.79]	70.81	0.71 [0.6, 0.81]; [0.38, 1.03]	96.86	9	0.72 [0.57, 0.87]; [0.33, 1.11]	91.18	0.61 [0.39, 0.83]; [0.03, 1.2]	95.72
Females	12	0.49 [0.35, 0.62]; [0.09, 0.88]	80.32	0.69 [0.58, 0.81]; [0.33, 1.05]	97.34	9	0.78 [0.69, 0.87]; [0.59, 0.96]	65.72	0.71 [0.49, 0.94]; [0.1, 1.33]	97.64
EF										
Males	13	0.67 [0.53, 0.81]; [0.22, 1.12]	90.11	0.44 [0.28, 0.6]; [−0.09, 0.97]	99.06	10	0.46 [0.34, 0.59]; [0.12, 0.81]	86.91	0.72 [0.57, 0.87]; [0.3, 1.13]	97.59
Females	13	0.68 [0.53, 0.84]; [0.19, 1.18]	90.55	0.54 [0.4, 0.68]; [0.09, 0.99]	98.67	10	0.37 [0.25, 0.49]; [0.04, 0.7]	86.25	0.78 [0.64, 0.91]; [0.4, 1.15]	97.86
EOE										
Males	12	0.21 [0.12, 0.31]; [−0.06, 0.49]	75.50	0.9 [0.85, 0.94]; [0.78, 1.01]	85.45	8	0.69 [0.58, 0.8]; [0.43, 0.96]	76.91	0.73 [0.59, 0.87]; [0.38, 1.08]	89.09
Females	12	0.53 [0.4, 0.65]; [0.17, 0.88]	75.43	0.67 [0.6, 0.73]; [0.49, 0.84]	83.87	8	0.65 [0.48, 0.82]; [0.21, 1.08]	90.05	0.65 [0.43, 0.87]; [0.05, 1.25]	96.77
DD										
Females	12	0.8 [0.73, 0.86]; [0.68, 0.92]	28.36	0.34 [0.22, 0.46]; [−0.03, 0.71]	96.73	8	0.56 [0.33, 0.79]; [−0.07, 1.19]	95.64	0.6 [0.44, 0.75]; [0.19, 1]	91.38
SR										
Males	12	0.51 [0.4, 0.62]; [0.21, 0.81]	71.11	0.66 [0.55, 0.77]; [0.31, 1]	96.84	9	0.69 [0.55, 0.83]; [0.35, 1.03]	86.29	0.64 [0.58, 0.71]; [0.54, 0.75]	30.95
Females	12	0.48 [0.37, 0.58]; [0.2, 0.76]	65.45	0.69 [0.55, 0.82]; [0.27, 1.11]	98.03	9	0.69 [0.58, 0.81]; [0.44, 0.95]	76.85	0.53 [0.32, 0.74]; [−0.05, 1.1]	95.92
SE										
Males	12	0.58 [0.47, 0.68]; [0.3, 0.86]	69.56	0.61 [0.52, 0.69]; [0.36, 0.85]	92.73	9	0.49 [0.4, 0.59]; [0.29, 0.7]	57.90	0.7 [0.66, 0.75]; [0.65, 0.75]	4.32
Females	12	0.69 [0.62, 0.76]; [0.56, 0.81]	25.09	0.51 [0.41, 0.6]; [0.22, 0.79]	94.39	9	0.85 [0.82, 0.89]; [0.82, 0.89]	0.00	0.41 [0.28, 0.53]; [0.09, 0.72]	84.85

*Abbreviations:FR* Food Responsiveness, *EF* Enjoyment of Food, *EOE* Emotional over-eating, *SR* Satiety Responsiveness, *N* Number of, *Se* Sensitivity, *Sp* Specificity, *CI* Confidence Interval, *PI* Prediction Interval

^a^Children aged ≥ 2 to < 7 years

^b^Children aged ≥ 7 to < 12 years

^c^Representing results from random effects meta-analyses after applying pooled-sample cut-offs to each cohort

## Discussion

This study synthesised cross-sectional IPD on CEBQ-measured appetitive traits and BMI-SDS from studies previously identified in a completed systematic review and meta-analysis [8]. It is the first to explore the potential of the CEBQ as a tool to characterise children’s eating behaviour-related obesity phenotypes, with a view to support personalised intervention approaches in future research and clinical practice. The ability of the CEBQ to discriminate child weight status varied across appetitive trait subscales, age- and sex-specific subgroups, and cohorts. While pooled discrimination was modest, discriminatory potential was observed in specific subgroups for the subscales of FR (middle childhood), EOE (middle childhood), DD (middle childhood in females only), SR (middle childhood), SE (middle childhood), and EF (early childhood). Cut-off scores were derived within age- and sex-specific subgroups using two different methods, resulting in varying combinations of sensitivity and specificity.

Overall, the findings suggest that certain CEBQ subscales may be suitable for use in some age- and sex- groups for personalised treatment approaches that tailor interventions for children with obesity based on their individual appetitive traits. For example, an FR cut-off of 2.5 (Se = 75%, Sp = 81%) for males and 2.6 (Se = 81%, Sp = 85%) for females in middle childhood led to sensitivity and specificity levels above 75%, indicating that more than 75% of children were correctly classified as living with or without obesity. In practice, this score may be used to assign children scoring above the cut-off to a treatment specifically targeting food responsiveness, while other children may receive a different intervention. However, high heterogeneity in sensitivity and specificity persisted when applying cut-offs to included cohorts, suggesting variability in their performance across different contexts. Prediction intervals, indicating the range within which sensitivity and specificity values of future studies applying the cut-offs would fall, were also generally wide. Thus, future research may investigate how sample- and individual characteristics that were not assessed in this study, such as children’s local food environment, socioeconomic status or cultural background, may influence the external validity of cut-off scores. Additionally, in order to mitigate potential unintended harms of applying appetitive trait-based classifications, such as shifts in parents’ or children’s self-perceptions, and more controlling or coercive feeding practices, cut-off scores should be embedded within sensitive clinical assessment, potentially alongside additional anthropometric and behavioural measures, and communicated in ways that minimise stigma and avoid deterministic interpretation. Findings also suggest that EUE, FF, and DD (in males) are not useful behavioural phenotypes upon which to base personalised behavioural interventions for children’s weight management, as their discriminatory ability was limited.

The finding that FR showed the highest accuracy in classifying weight status is of particular interest given recent evidence linking high FR in early childhood to higher odds of developing eating disorder symptoms [52]. This stresses the potential utility of FR not only for tailoring weight management advice but also for identifying children who may benefit from closer monitoring to mitigate emerging eating disorder risk. The limited discriminatory abilities of EUE, FF, and DD are in line with previous research reporting similar findings, where EUE, FF and DD are often only weakly or not significantly associated with measures of overweight/obesity [53, 54]. These traits are also less commonly measured across studies, which limits the overall strength and consistency of the available evidence. A possible explanation is that FF typically determines the content of what children are eating - rather than their total energy intake - which could be related to both over- or underweight [55]. Similarly, DD assesses general wanting for drinks without distinguishing between options such as sugar-sweetened, non-nutritively sweetened and unsweetened beverages [6]. Although some studies have reported an association between DD and preference for sweet drinks, [54] a modified subscale version that more clearly differentiates between types of drink may be needed to enhance DD’s ability to discriminate weight status.

Apart from EF, the discriminatory ability of appetitive traits was consistently lower in early childhood subgroups compared to middle childhood. This pattern likely reflects, in part, that fewer children will have developed obesity at this early stage of life, making it harder to distinguish between potential phenotypes. It also aligns with Behavioural Susceptibility Theory (BST), proposing that children’s appetitive traits interact with the obesogenic environment: [56] As older children with increasing autonomy experience greater exposure to their food environment, they may display more pronounced differences in eating behaviour traits and obesity phenotypes. EF was the only subscale where discriminatory potential was lower in middle childhood compared to early childhood. The expression of EF in children with a healthy weight and with overweight, as well as its relationship with the development of obesity over time, remains to be investigated more closely in future research.

### Strengths and limitations

Pooling IPD from 17 systematically identified studies constitutes a major strength of this study, allowing for the generation of a large sample size (*n* = 15,273), thus enhancing statistical power and enabling subgroup analyses by age and sex. It further enabled us to explore heterogeneity of estimates across different populations from various geographical locations (e.g. UK, US, Netherlands), thus enhancing insights into the external validity of findings. Additionally, applying and comparing two methods to derive cut-off scores, using both Youden’s Index and a minimum sensitivity of 60%, allowed for a more flexible and nuanced interpretation by offering alternative thresholds for application based on user priorities and by providing insights into the variability of cut-off scores under different methodological assumptions. Lastly, the CEBQ is a psychometrically sound and widely used questionnaire, which increases the utility of the derived cut-offs.

However, synthesising findings from multiple cohorts also presents various challenges and limitations. Firstly, not all eligible studies were included, which could raise concerns about selection bias and external validity. Furthermore, fewer studies included children older than 10 years, resulting in smaller sample sizes in the older age groups and preventing us from being able to provide cut-off scores for the late childhood age group. Additionally, older participants were more commonly from clinical samples as opposed to community or birth cohorts, where the prevalence of obesity is generally lower (see Table SM 6 − 1). These differences in obesity spectrum limit the ability to draw conclusions from the comparisons of age groups and introduces the risk of distorted or biased accuracy estimates. Although we conducted prespecified meta-regressions to test potential sources of heterogeneity, the inclusion of samples spanning both clinical and population-based cohorts across different countries and cultural contexts may present further challenges in the generalisability of findings. Future research on the CEBQ’s potential to characterise eating behaviour phenotypes of obesity should randomly select participants from the target population of interest to avoid such limitations.

Implications following from the choice of measures also need to be considered when interpreting results. Although BMI-SDS is considered the best-practice and most practical measure to screen for obesity, studies may explore the CEBQ’s performance in relation to more direct measures of obesity, such as body composition. Furthermore, due to its parent-reported nature, the CEBQ might be subject to social desirability and test interpretation bias. However, the subscales of SR, SE, FR and EF have been cross validated with behavioural measures of eating behaviours, reducing the concerns of response biases for these subscales [42]. Moreover, a meta-analysis on biases in accuracy studies found the evidence on test interpretation bias to be mixed, with some studies finding no effects of blinding - comparable to parents being unaware of their child’s weight status - on accuracy estimates [57]. Given the restrictions in accessing extensive sets of covariates for secondary IPD meta-analysis, we were unable to control for other aspects that may influence the expression of appetitive traits. Future research is required to examine the impact of potential influences such as socioeconomic status, parental feeding practices, and environmental aspects on cut-off score performance. Lastly, our search was conducted up to February 2019, and the included datasets are therefore restricted to studies published before this date. However, the study identification and selection process was undertaken solely to generate a dataset for original analyses, rather than to synthesise existing evidence. Accordingly, the findings remain relevant irrespective of the search date.

### Directions for future research

Initial trials investigating SR- and FR-based treatments in children show promising results, encouraging further research on appetitive trait-based interventions [11–14]. Since, according to BST, the expression of traits like FR depend on the exposure to palatable food cues, these interventions may also benefit from targeting children’s proximal food environments, such as the home, alongside individual behaviour. Future trials may investigate whether such treatments are particularly helpful for children exhibiting the targeted eating behaviour phenotype, as determined by using the cut-off scores provided in this study. This would allow for more informed personalised treatment approaches for the future. Additionally, recent studies have started to investigate how multiple appetitive traits interact and cluster together to form appetitive profiles [58, 59]. Future research may thus explore how the cut-off scores derived here relate to emerging clusters. Individuals exceeding multiple cut-offs may represent distinct phenotypes, and combining cut-off-based classification with an understanding of appetite profiles could enhance precision in early identification of children for personalised interventions. Patient and public involvement and engagement (PPIE) will be particularly useful to inform the optimal implementation of such cut-off scores. Furthermore, future research may extend the current findings by using data from longitudinal studies of appetitive traits, particularly from preschool aged children, to investigate whether CEBQ scores can predict obesity risk at later developmental stages. Such early identification could enable timely prevention of excess weight gain and associated health consequences through personalised, eating-behaviour-trait-sensitive guidance.

## Conclusions

To our knowledge, this study is the first to explore the CEBQ’s potential as a tool for phenotyping childhood obesity based on appetitive traits. The findings suggest that cut-off scores for six of the eight CEBQ measured appetitive traits (FR, EF, EOE, DD, SR and SE) could be meaningful in characterising appetitive trait-based obesity and inform personalised treatment approaches, though their utility differs by trait, age and sex. In research, these cut-off scores could be used to assign participants to trials targeting specific appetitive traits, and in clinical practice they could assist decision-making to identify children who may benefit from particular types of behavioural support. For example, among children aged 7 to 12 years, FR showed the most robust discrimination and could identify children suited to food cue-based interventions alongside modifications of the home environment, while SR and EOE may flag children for appetite-awareness training and emotional-eating support. EF (in younger children), DD (in females), and SE provided weaker, more tentative signals and should be applied with greater caution. If integrated into routine care and embedded within broader clinical judgement, cut-offs could enhance treatment effectiveness by enabling more targeted intervention strategies that respond to children’s individual needs.

## Supplementary Information

Supplementary Material 1.

## Data Availability

This study used secondary data obtained from original investigators. Thus, any request for datasets supporting the conclusions of this article should be made to original owners of data. An analysis plan is available on the Open Science Framework (DOI 10.17605/OSF.IO/B49TU). Analysis code used to inform the conclusions of this article will be made available upon request to the corresponding author.

## Abbreviations

USD: United states dollars
CEBQ: Child eating behaviour questionnaire
FR: Food responsiveness
EF: Enjoyment of Food
EOE: Emotional over-eating
DD: Desire to drink
SR: Satiety responsiveness
SE: Slowness in eating
EUE: Emotional under-eating
FF: Food fussiness
IPD: Individual participant data
BMI-SDS: Body mass index standard deviation scores
OR: Odds ratio
CI: Confidence interval
AUC: Area under the curve
ROC: Receiver operating curve
Se: Sensitivity
Sp: Specificity
