# Supplementary material for: Identifying eating behaviour phenotype cut-off scores for childhood obesity: an individual participant data meta-analysis using the Child Eating Behaviour Questionnaire (CEBQ)
Source: Int J Behav Nutr Phys Act. 2026 Jul 25;23:98. doi: 10.1186/s12966-026-01951-8 (PMC13693115; doi:10.1186/s12966-026-01951-8)
Supplement: Supplementary file 1 [file 12966_2026_1951_MOESM1_ESM.pdf]

Identifying eating behaviour phenotype cut-off scores for childhood obesity: An individual participant data meta-analysis using the Child Eating Behaviour Questionnaire (CEBQ)

[SUPPLEMENTARY MATERIAL]

Contents

1.0 Child Eating Behaviour Questionnaire (CEBQ) and Scoring Instructions 5
1.1 Scoring of the CEBQ 7
1.2 Reference, use and reproduction of the CEBQ 7
2.0 PRISMA-IPD Checklist 8
3.0 PICO(S) Terms 11
4.0 Electronic search strategy 12
5.0 Risk of Bias assessment 13
5.1 Newcastle-Ottawa-Scale (NOS) scoring adaptions 13
6.0 Study characteristics 15
7.0 Biologically Implausible Values (BIV) excluded from further analysis 17
8.0 Average CEBQ subscale scores of included cohorts 18
9.0 Number of missing values for CEBQ subscales 19
10.0 Number of children in BMI-SDS groups 19
11.0 Quintile scores on CEBQ subscales 21
12.0 Ridgeline plots of the distribution of CEBQ subscale scores across BMI-SDS groups within Cohorts 24
13.0 Receiver Operating Curves (ROC) of CEBQ subscale scores across cohorts 32
14.0 Forest Plots of AUCs 40
15.0 Meta-regression exploring impact of pre-defined study characteristics on AUCs 47
15.1 Subgroup analyses 48
16.0 Shape of association between CEBQ subscale scores and BMI-SDS groups 49
17.0 Cut-off scores derived with a fixed sensitivity level of at least 60% 53

Tables

Table SM 2-1: PRISMA-IPD Checklist indicating the manuscripts fulfilment of outlined criteria........................8

Table SM 3-1: PICO(S) terms indicating eligibility criteria guiding the identification and inclusion of studies in the preceding meta-analysis 11

Table SM 3-2: PICO(S) terms indicating eligibility criteria guiding the identification and inclusion of studies in the current study 11

Table SM 4-1: Search terms as applied to MEDLINE (Ovid)...12

Table SM 5-1: Newcastle-Ottawa quality assessment scale for included cohorts...13

Table SM 6-1: Detailed characteristics of included studies that provided IPD as extracted from published manuscripts 15

Table SM 7-1: Total number of observations for all appetitive trait subscales, including missing and Biologically Implausible Values (BIV) 17

Table SM 8-1: Average CEBQ scores of included studies as derived from individual participant data..............18

Table SM 9-1: Number of missing CEBQ scores of included studies as derived from individual participant data 19

Table SM 10-1: Number of children in BMI-SDS groups by CEBQ subscale and cohort as derived from individual participant data...19

Table SM 11-1: Quintile scores for the CEBQ subscale Food Responsiveness..21

Table SM 11-2: Quintile scores for the CEBQ subscale Emotional Overeating..21

Table SM 11-3: Quintile scores for the CEBQ subscale Enjoyment of Food 21

Table SM 11-4: Quintile scores for the CEBQ subscale Desire to Drink..22

Table SM 11-5: Quintile scores for the CEBQ subscale Satiety Responsiveness..22

Table SM 11-6: Quintile scores for the CEBQ subscale Slowness in Eating...22

Table SM 11-7: Quintile scores for the CEBQ subscale Food Fussiness..22

Table SM 11-8: Quintile scores for the CEBQ subscale Emotional Undereating..23

Table SM 15-1: Heterogeneity estimates from meta-regression models testing the impact of predefined study characteristics on area under the curve estimates 47

Table SM 15-2: Meta-regression models testing the impact of predefined study characteristics on area under the curve (AUC) estimates...47

Table SM 15-3: Meta-analysis of AUC results for cohorts administering the CEBQ in English 48

Table SM 15-4: Meta-analysis of AUC results for cohorts NOT administering the CEBQ in English................48

Table SM 15-5: Meta-analysis of AUC results for cohorts classified as LOW risk of bias.................................48

Table SM 15-6: Meta-analysis of AUC results for cohorts classified as SOME risk of bias...............................48

Table SM 17-1: Age- and sex-specific cut-off scores to identify weight status (no obesity a vs obesity b) as derived using a minimum sensitivity level of 60%...53

Table SM 17-2: Random-Effects Meta-Analysis of Cut-Off Score Performance (Minimum Sensitivity 60%) and Heterogeneity Across Cohorts 54

Figures

Figure SM 12-1 Distribution of Food Responsiveness (FR) scores across BMI-SDS Groups within Cohorts 24
Figure SM 12-2 Distribution of Emotional Overeating (EOE) scores across BMI-SDS Groups within Cohorts.25
Figure SM 12-3 Distribution of Enjoyment of Food (EF) scores across BMI-SDS Groups within Cohorts 26
Figure SM 12-4 Distribution of Desire to Drink (DD) scores across BMI-SDS Groups within Cohorts 27
Figure SM 12-5 Distribution of Satiety Responsiveness (SR) scores across BMI-SDS Groups within Cohorts..28
Figure SM 12-6 Distribution of Slowness in Eating (SE) scores across BMI-SDS Groups within Cohorts.........29
Figure SM 12-7 Distribution of Food Fussiness (FF) scores across BMI-SDS Groups within Cohorts 30
Figure SM 12-8 Distribution of Emotional Undereating (EUE) scores across BMI-SDS Groups within Cohorts 31
Figure SM 13-1 Receiver Operating Curve (ROC) of Food Responsiveness (FR) scores across Cohorts 32
Figure SM 13-2 Receiver Operating Curve (ROC) of Emotional Overeating (EOE) scores across Cohorts 33
Figure SM 13-3 Receiver Operating Curve (ROC) of Enjoyment of Food (EF) scores across Cohorts 34
Figure SM 13-4 Receiver Operating Curve (ROC) of Desire to Drink (DD) scores across Cohorts 35
Figure SM 13-5 Receiver Operating Curve (ROC) of Satiety Responsiveness (SR) scores across Cohorts 36
Figure SM 13-6 Receiver Operating Curve (ROC) of Slowness in Eating (SE) scores across Cohorts 37
Figure SM 13-7 Receiver Operating Curve (ROC) of Food Fussiness (FF) scores across Cohorts......................38
Figure SM 13-8 Receiver Operating Curve (ROC) of Emotional Undereating (EUE) scores across Cohorts 39
Figure SM 14-1 Forest Plot of Food Responsiveness (FR) Scores across Cohorts 40
Figure SM 14-2 Forest Plot of Emotional Overeating (EOE) Scores across Cohorts 41
Figure SM 14-3 Forest Plot of Enjoyment of Food (EF) Scores across Cohorts..42
Figure SM 14-4 Forest Plot of Desire to Drink (DD) Scores across Cohorts...43
Figure SM 14-5 Forest Plot of Satiety Responsiveness (SR) Scores across Cohorts 44
Figure SM 14-6 Forest Plot of Slowness in Eating (SE) Scores across Cohorts 44
Figure SM 14-7 Forest Plot of Food Fussiness (FF) Scores across Cohorts 45
Figure SM 14-8 Forest Plot of Emotional Undereating (EUE) Scores across Cohorts 46
Figure SM 16-1 Shape of Association of Food Responsiveness (FR) and BMI-SDS Group in stratified sex- and age- groups 49
Figure SM 16-2 Shape of Association of Emotional Overeating (EOE) and BMI-SDS Group in stratified sex- and age- groups 49
Figure SM 16-3 Shape of Association of Enjoyment of Food (EF) and BMI-SDS Group in stratified sex- and age- groups 50
Figure SM 16-4 Shape of Association of Desire to Drink (DD) and BMI-SDS Group in stratified sex- and age- groups 50
Figure SM 16-5 Shape of Association of Satiety Responsiveness (SR) and BMI-SDS Group in stratified sex- and age- groups 51
Figure SM 16-6 Shape of Association of Slowness in Eating (SE) and BMI-SDS Group in stratified sex- and age- groups 51
Figure SM 16-7 Shape of Association of Food Fussiness (FF) and BMI-SDS Group in stratified sex- and age- groups 52
Figure SM 16-8 Shape of Association of Emotional Undereating (EUE) and BMI-SDS Group in stratified sex- and age- groups 52

1.0 Child Eating Behaviour Questionnaire (CEBQ) and Scoring Instructions

Please read the following statements and tick the boxes most appropriate to your child's eating behaviour.

	**Never**	**Rarely**	**Sometimes**	**Often**	**Always**
My child loves food (EF)	□	□	□	□	□
My child eats more when worried (EOE)	□	□	□	□	□
My child has a big appetite (SR*)	□	□	□	□	□
My child finishes his/her meal quickly (SE*)	□	□	□	□	□
My child is interested in food (EF)	□	□	□	□	□
My child is always asking for a drink (DD)	□	□	□	□	□
My child refuses new foods at first (FF)	□	□	□	□	□
My child eats slowly (SE)	□	□	□	□	□
My child eats less when angry (EUE)	□	□	□	□	□
My child enjoys tasting new foods (FF*)	□	□	□	□	□
My child eats less when s/he is tired (EUE)	□	□	□	□	□
My child is always asking for food (FR)	□	□	□	□	□
My child eats more when annoyed (EOE)	□	□	□	□	□
If allowed to, my child would eat too much (FR)	□	□	□	□	□
My child eats more when anxious (EOE)	□	□	□	□	□
My child enjoys a wide variety of foods (FF*)	□	□	□	□	□

My child leaves food on his/her plate at the end of a meal (SR)	□	□	□	□	□
My child takes more than 30 minutes to finish a meal (SE)	□	□	□	□	□
Given the choice, my child would eat most of the time (FR)	□	□	□	□	□
My child looks forward to mealtimes (EF)	□	□	□	□	□
My child gets full before his/her meal is finished (SR)	□	□	□	□	□
My child enjoys eating (EF)	□	□	□	□	□
My child eats more when she is happy (EUE)	□	□	□	□	□
My child is difficult to please with meals (FF)	□	□	□	□	□
My child eats less when upset (EUE)	□	□	□	□	□
My child gets full up easily (SR)	□	□	□	□	□
My child eats more when s/he has nothing else to do (EOE)	□	□	□	□	□
Even if my child is full up s/he finds room to eat his/her favourite food (FR)	□	□	□	□	□
If given the chance, my child would drink continuously throughout the day (DD)	□	□	□	□	□
My child cannot eat a meal if s/he has had a snack just before (SR)	□	□	□	□	□
If given the chance, my child would always be having a drink (DD)	□	□	□	□	□
My child is interested in tasting food s/he hasn't tasted before (FF*)	□	□	□	□	□

My child decides that s/he doesn't like a food, even without tasting it (FF)	□	□	□	□	□
If given the chance, my child would always have food in his/her mouth (FR)	□	□	□	□	□
My child eats more and more slowly during the course of a meal (SE)	□	□	□	□	□

1.1 Scoring of the CEBQ

(Never = 1, Rarely = 2, Sometimes = 3, Often = 4, Always = 5)

Food responsiveness	=	item mean FR
Emotional over-eating	=	item mean EOE
Enjoyment of food	=	item mean EF
Desire to drink	=	item mean DD
Satiety responsiveness	=	item mean SR
Slowness in eating	=	item mean SE
Emotional under-eating	=	item mean EUE
Food fussiness	=	item mean FF

*Reversed items

1.2 Reference, use and reproduction of the CEBQ

Reference: Wardle, J, Guthrie CA, Sanderson, S and Rapoport, L. Development of the Children's Eating Behaviour Questionnaire. *Journal of Child Psychology and Psychiatry.* 42, 2001, 963-970.

Please note there is an error in the text of this paper concerning the scoring of the CEBQ which is given as 0 - 4. In fact, responses were scored 1- 5 and the means and standard deviations given in the tables reflect this.

Any use or reproduction of the CEBQ for academic research or non-profit organizations requires a ***free license***, which can be obtained here:

https://xip.uclb.com/product/child-eating-behaviour-questionnaire-cebq

2.0 PRISMA-IPD Checklist

Table SM 2-1: PRISMA-IPD Checklist indicating the manuscripts fulfilment of outlined criteria

PRISMA-IPD Section/topic	Item No	Checklist item	Reported on page
**Title**			
Title	1	Identify the report as a systematic review and meta-analysis of individual participant data.	1
**Abstract**			
Structured summary	2	Provide a structured summary including as applicable:	3
		**Background**: state research question and main objectives, with information on participants, interventions, comparators and outcomes.	
		**Methods**: report eligibility criteria; data sources including dates of last bibliographic search or elicitation, noting that IPD were sought; methods of assessing risk of bias.	
		**Results**: provide number and type of studies and participants identified and number (%) obtained; summary effect estimates for main outcomes (benefits and harms) with confidence intervals and measures of statistical heterogeneity. Describe the direction and size of summary effects in terms meaningful to those who would put findings into practice.	
		**Discussion:** state main strengths and limitations of the evidence, general interpretation of the results and any important implications.	
		**Other:** report primary funding source, registration number and registry name for the systematic review and IPD meta-analysis.	
**Introduction**			
Rationale	3	Describe the rationale for the review in the context of what is already known.	5
Objectives	4	Provide an explicit statement of the questions being addressed with reference, as applicable, to participants, interventions, comparisons, outcomes and study design (PICOS). Include any hypotheses that relate to particular types of participant-level subgroups.	6
**Methods**			
Protocol and registration	5	Indicate if a protocol exists and where it can be accessed. If available, provide registration information including registration number and registry name. Provide publication details, if applicable.	7
Eligibility criteria	6	Specify inclusion and exclusion criteria including those relating to participants, interventions, comparisons, outcomes, study design and characteristics (e.g. years when conducted, required minimum follow-up). Note whether these were applied at the study or individual level i.e. whether eligible participants were included (and ineligible participants excluded) from a study that included a wider population than specified by the review inclusion criteria. The rationale for criteria should be stated.	7
Identifying studies - information sources	7	Describe all methods of identifying published and unpublished studies including, as applicable: which bibliographic databases were searched with dates of coverage; details of any hand searching including of conference proceedings; use of study registers and agency or company databases; contact with the original research team and experts in the field; open adverts and surveys. Give the date of last search or elicitation.	7
Identifying studies - search	8	Present the full electronic search strategy for at least one database, including any limits used, such that it could be repeated.	SM 12
Study selection processes	9	State the process for determining which studies were eligible for inclusion.	7
Data collection processes	10	Describe how IPD were requested, collected and managed, including any processes for querying and confirming data with investigators. If IPD were not sought from any eligible study, the reason for this should be stated (for each such study).	7-8
		If applicable, describe how any studies for which IPD were not available were dealt with. This should include whether, how and what aggregate data were sought or extracted from study reports and publications (such as extracting data independently in duplicate) and any processes for obtaining and confirming these data with investigators.	
Data items	11	Describe how the information and variables to be collected were chosen. List and define all study level and participant level data that were sought, including baseline and follow-up information. If applicable, describe methods of standardising or translating variables within the IPD datasets to ensure common scales or measurements across studies.	8-9
IPD integrity	A1	Describe what aspects of IPD were subject to data checking (such as sequence generation, data consistency and completeness, baseline imbalance) and how this was done.	8
Risk of bias assessment in individual studies.	12	Describe methods used to assess risk of bias in the individual studies and whether this was applied separately for each outcome. If applicable, describe how findings of IPD checking were used to inform the assessment. Report if and how risk of bias assessment was used in any data synthesis.	8

Specification of outcomes and effect measures	13	State all treatment comparisons of interests. State all outcomes addressed and define them in detail. State whether they were pre-specified for the review and, if applicable, whether they were primary/main or secondary/additional outcomes. Give the principal measures of effect (such as risk ratio, hazard ratio, difference in means) used for each outcome.	8-9
Synthesis methods	14	Describe the meta-analysis methods used to synthesise IPD. Specify any statistical methods and models used. Issues should include (but are not restricted to):<br>• Use of a one-stage or two-stage approach.<br>• How effect estimates were generated separately within each study and combined across studies (where applicable).<br>• Specification of one-stage models (where applicable) including how clustering of patients within studies was accounted for.<br>• Use of fixed or random effects models and any other model assumptions, such as proportional hazards.<br>• How (summary) survival curves were generated (where applicable).<br>• Methods for quantifying statistical heterogeneity (such as I^2 and t^2).<br>• How studies providing IPD and not providing IPD were analysed together (where applicable).<br>• How missing data within the IPD were dealt with (where applicable).	9-11
Exploration of variation in effects	A2	If applicable, describe any methods used to explore variation in effects by study or participant level characteristics (such as estimation of interactions between effect and covariates). State all participant-level characteristics that were analysed as potential effect modifiers, and whether these were pre-specified.	10-11
Risk of bias across studies	15	Specify any assessment of risk of bias relating to the accumulated body of evidence, including any pertaining to not obtaining IPD for particular studies, outcomes or other variables.	11
Additional analyses	16	Describe methods of any additional analyses, including sensitivity analyses. State which of these were pre-specified.	11
**Results**			
Study selection and IPD obtained	17	Give numbers of studies screened, assessed for eligibility, and included in the systematic review with reasons for exclusions at each stage. Indicate the number of studies and participants for which IPD were sought and for which IPD were obtained. For those studies where IPD were not available, give the numbers of studies and participants for which aggregate data were available. Report reasons for non-availability of IPD. Include a flow diagram.	11
Study characteristics	18	For each study, present information on key study and participant characteristics (such as description of interventions, numbers of participants, demographic data, unavailability of outcomes, funding source, and if applicable duration of follow-up). Provide (main) citations for each study. Where applicable, also report similar study characteristics for any studies not providing IPD.	SM 15-20
IPD integrity	A3	Report any important issues identified in checking IPD or state that there were none.	12-13
Risk of bias within studies	19	Present data on risk of bias assessments. If applicable, describe whether data checking led to the up-weighting or down-weighting of these assessments. Consider how any potential bias impacts on the robustness of meta-analysis conclusions.	SM 13
Results of individual studies	20	For each comparison and for each main outcome (benefit or harm), for each individual study report the number of eligible participants for which data were obtained and show simple summary data for each intervention group (including, where applicable, the number of events), effect estimates and confidence intervals. These may be tabulated or included on a forest plot.	SM 19-20;<br>SM 40-46
Results of syntheses	21	Present summary effects for each meta-analysis undertaken, including confidence intervals and measures of statistical heterogeneity. State whether the analysis was pre-specified, and report the numbers of studies and participants and, where applicable, the number of events on which it is based.<br>When exploring variation in effects due to patient or study characteristics, present summary interaction estimates for each characteristic examined, including confidence intervals and measures of statistical heterogeneity. State whether the analysis was pre-specified. State whether any interaction is consistent across trials.<br>Provide a description of the direction and size of effect in terms meaningful to those who would put findings into practice.	13-15
Risk of bias across studies	22	Present results of any assessment of risk of bias relating to the accumulated body of evidence, including any pertaining to the availability and representativeness of available studies, outcomes or other variables.	15
Additional analyses	23	Give results of any additional analyses (e.g. sensitivity analyses). If applicable, this should also include any analyses that incorporate aggregate data for studies that do not have IPD. If applicable, summarise the main meta-analysis results following the inclusion or exclusion of studies for which IPD were not available.	15
**Discussion**			
Summary of evidence	24	Summarise the main findings, including the strength of evidence for each main outcome.	15-17
Strengths and limitations	25	Discuss any important strengths and limitations of the evidence including the benefits of access to IPD and any limitations arising from IPD that were not available.	17-19
Conclusions	26	Provide a general interpretation of the findings in the context of other evidence.	20

Implications	A4	Consider relevance to key groups (such as policy makers, service providers and service users). Consider implications for future research.	19-20
**Funding**			
Funding	27	Describe sources of funding and other support (such as supply of IPD), and the role in the systematic review of those providing such support.	21

A1 – A3 denote new items that are additional to standard PRISMA items. A4 has been created as a result of re-arranging content of the standard PRISMA statement to suit the way that systematic review IPD meta-analyses are reported.

© Reproduced with permission of the PRISMA IPD Group, which encourages sharing and reuse for non-commercial purposes

3.0 PICO(S) Terms

Table SM 3-1: PICO(S) terms indicating eligibility criteria guiding the identification and inclusion of studies in the preceding meta-analysis

**Inclusion criteria**	
PICOs	Eligibility
Population	• Children younger or equal to 18 years
Study type	• Observational Studies
Predictor	• At least one reported CEBQ or BEBQ measured appetitive trait
Outcomes	• Any measure of adiposity (e.g. BMI-SDS, BMI percentile, Waist Circumference, Body composition)
**Exclusion criteria**	
• CEBQ/BEBQ subscales modified from their original version (e.g. reorganised into new dimensions)<br>• Manuscript not published in English language and no available translation	

Table SM 3-2: PICO(S) terms indicating eligibility criteria guiding the identification and inclusion of studies in the current study

**Inclusion criteria**	
PICOs	Eligibility
Population	• Children aged between two and 18 years
Study type	• Cross-sectional observational study cohorts
Predictor	• Data allowing computation of at least one original CEBQ appetitive trait
Outcomes	• Data allowing computation of BMI-SDS according to WHO reference standards
**Exclusion criteria**	
• CEBQ items/subscales modified from their original version (e.g. child self-report, reduced number of items)<br>• Manuscript not published in English language and no available translation<br>• Essential information not provided after three reminders (e.g. reversed status of item-level data)	

4.0 Electronic search strategy

Table SM 4-1: Search terms as applied to MEDLINE (Ovid)

**1**	exp CHILD/ or exp ADOLESCENT/ or exp CHILD, PRESCHOOL/ or CHILD/ or exp INFANT/
**2**	(child* or adolescen* or infant*).mp
**3**	(teenage* or young people or young person or young adult*).mp.
**4**	(schoolchildren or school children).mp.
**5**	(pediatr* or paediatr*).mp
**6**	(boys or girls or youth or youths).mp.
**7**	1 or 2 or 3 or 4 or 5 or 6
**8**	(Child* Eating Behavio?r Questionnaire or CEBQ).mp or appet* traits.mp or eating behaviours.mp or food fussiness.mp or food responsiveness.mp
**9**	(Emotional over-eating or emotional overeating or emotional eating or emotional over eating).mp
**10**	(enjoyment of food or desire to drink or satiety responsiveness or slowness in eating).mp
**11**	(Emotional under-eating or emotional undereating or emotional under eating)).mp.
**12**	8 or 9 or 10 or 11
**13**	(adipos* or (weight or weight status)).mp. or exp obesity/ or exp overweight/
**14**	BMI z-scores.mp. or BMI-for-age.mp. or weight-for-length percentiles.mp.
**15**	((weight-for-height percentiles or waist circumference) adj2 growth charts).mp. or skinfold thickness.mp. or Anthropometr*.mp. or
**16**	((weight or bmi or body mass index) adj2 (gain or loss or change or reduc*)).mp.
**17**	13 or 14 or 15 or 16
**18**	7 and 12 and 17
**19**	limit 18 to yr="2001 -Current"

**The syntax used in this search strategy was adjusted where necessary according to the requirements of each database.*

5.0 Risk of Bias assessment

Table SM 5-1: Newcastle-Ottawa quality assessment scale for included cohorts

Studies (n = 17)	Selection				Outcome	Total Score
	Representativeness of the sample	Sample size	Non-respondents	Ascertainment of exposure	Assessment of outcome	
Carnell & Wardle, 2008 [a]	B*	A*	A*	B*	D	4
Cross, 2014	C	B	A*	B*	A**	4
De Groot, 2017	C	B	C	B*	A**	3
Domoff, 2015	C	A*	B	A*	A**	4
Dos Passos, 2015	C	B	A*	B*	A**	4
Eloranta, 2012	B*	B	B	B*	A**	4
Emond, 2017	C	B	B	B*	A**	3
Escobar, 2014	A*	B	C	B*	A**	4
Gross, 2016	C	B	C	B*	B**	3
Hardman, 2016	B*	B	C	B*	D	2
Haycraft, 2011	B*	B	B	B*	D	2
Hayes, 2016	C	B	A*	B*	A**	4
McCarthy, 2015	A*	A*	A*	B*	A**	6
Garcia, 2016	C	A*	B	A*	A**	4
Spence, 2011	B*	A*	B	A*	A**	5
van Deutekom, 2016	A*	A*	A*	B*	B**	6
Viana, 2008	B*	B	C	B*	D	2

[a] only TEDs cohort data was received and assessed for quality using NOS

5.1 Newcastle-Ottawa-Scale (NOS) scoring adaptions

This scale has been adapted from the Newcastle-Ottawa Quality Assessment Scale for cohort studies to perform a quality assessment of cohort studies for the present Individual participant Data Meta-analysis. The NOS usually assesses three main areas of study quality, namely 1) the selection of the cohort, 2) the comparability of study analysis, and 3) the ascertainment of the outcome and rates each area using a star scoring system (see additional information about scoring procedure below). The summation of the stars is then used to generate the total score. For the present analysis, we removed the component of comparability, as no differential outcome groups were considered. We also removed items pertaining to the statistical test used, as this is not relevant for an individual participant data meta-analysis, where raw data is reanalysed as opposed to synthesised from study reports. Typically, 10 stars are maximally possible with NOS, and 7 or greater are considered good. In our adapted scale, a maximum of 7 stars is possible, and a score of 4 or greater was considered good (reflecting roughly 70% of possible scores)

Selection (max 5 stars)

1) Representativeness of the exposed cohort
A*= Truly representative of the general population
B* = Somewhat representative of the general population
C = Selected group of users e.g. nurses, volunteers
2) Sample size
A* = Justified and satisfactory
B = Not justified
3) Non-respondents
A* = Comparability between respondents' and non-respondents' characteristics is established, and the response rate is satisfactory
B = The response rate is unsatisfactory, or the comparability between respondents and non-respondents is unsatisfactory.
C = No description of the response rate or the characteristics of the respondents and the non-respondents
4) Ascertainment of exposure (CEBQ)
A** = Self or parent-administered questionnaire (with extra validation/reliability information reported for specific target sample)
B* = Parent or self-reported questionnaire

Outcome (max 2 stars)
1) Assessment of outcome (obesity)
A** = Clinical assessment
B** = Record linkage
C = Self-report
D = Parent-report

6.0 Study characteristics

Table SM 6-1: Detailed characteristics of included studies that provided IPD as extracted from published manuscripts

Authors, year	Country	Sample source	CEBQ language	Subscales included	N (%female)	Age range	Age Mean (SD)	BMI-SDS range	BMI-SDS Mean (SD)	%ob
Carnell & Wardle, 2008 [2]	United Kingdom	TEDs	English	EF	5440 (51.08)	8 - 11	9.91 (0.86)	-2.93 - 6.30	0.15 (1.19)	6.75
Cross et al., 2014 + Frankel et al., 2014 [3]	United States	(Head Start) low-income preschool sample	English and Spanish	SR, SE, EUE, FF, FR, EF, EOE, DD	209 (52.15)	2 - 5	4.41 (0.66)	-2.74 - 7.79	1.03 (1.67)	22.49
De Groot et al., 2017 [4]	Netherlands	community sample	Dutch	SR, SE, EUE, FF, FR, EF, EOE, DD	44 (52.27)	12 - 16	14.80 (1.43)	-1.96 - 5.40	1.63 (1.98)	56.82
Domoff et al., 2015 [5]	United States	(Head Start) low-income preschool sample from ABC and "Growing Healthy" cohorts	English	SR, SE, EUE, FF, FR, EF, EOE, DD	989 (50.66)	2 - 6	4.15 (0.53)	-2.54 - 6.62	0.87 (1.20)	13.25
Dos Passos et al., 2015 [6]	Brazil	community sample	Portuguese	SR, SE, EUE, FF, FR, EF, EOE, DD	331 (51.66)	5 - 10	7.41 (0.87)	-1.93 - 6.17	1.17 (1.44)	24.77
Eloranta et al., 2012 [7]	Finland	Baseline PANIC (School-based diet and exercise intervention)	Finnish	SR, SE, EUE, FF, FR, EF, EOE, DD	478 (48.74)	6 - 8	7.63 (0.38)	-2.75 - 3.83	0.14 (1.12)	6.90
Emond et al., 2017 [8]	United States	community sample	English	SR, SE, EUE, FF, FR, EF, EOE, DD	178 (51.12)	8 - 11	9.93 (0.58)	-1.55 - 3.82	0.63 (1.19)	14.04
Escobar et al., 2014 [9]	Canada	Maternal Adversity, Vulnerability and Neurodevelopment (MAVAN) birth cohort	English	SR, SE, EUE, FF, FR, EF, EOE, DD	346 (46.24)	3 - 4	4.11 (0.10)	-1.36 - 6.05	0.48 (1.01)	6.07
Gross et al., 2016 [10]	United States	Healthy You! routine clinic sample	English	SR, SE, EUE, FF, FR, EF, EOE, DD	817 (53.37)	2 - 12	9.62 (2.50)	-0.44 - 7.91	3.65 (1.22)	96.57
Hardman et al., 2016 [11]	United Kingdom	community sample	English	SR, SE, EUE, FF, FR, EF, EOE, DD	79 (49.37)	3 - 13	8.71 (1.99)	-2.97 - 5.46	0.51 (1.71)	21.52
Haycraft et al., 2011 [12]	United Kingdom	community sample	English	SR, SE, EUE, FF, FR, EF, EOE, DD	137 (46.72)	3 - 8	5.11 (1.43)	-2.74 - 7.83	0.30 (1.52)	14.60
Hayes et al., 2016 [13]	United States	Family-based Behavioural Treatment (FBT) baseline sample	English	SR, SE, EUE, FF, FR, EF, EOE, DD	241 (62.66)	7 - 12	9.93 (1.32)	1.20 - 7.77	3.15 (0.92)	92.53
McCarthy et al., 2015 [14]	Ireland	BASELINE birth cohort	English	EF, EUE, EOE, DD, FF	1189 (48.70)	2 - 2	2.15 (0.09)	-2.13 - 4.91	0.70 (0.91)	7.82
Rudy et al., 2018 + Silva Garcia et al., 2016 [15]	United States	(Headstart) low-income preschool sample	English	SR, SE, EUE, FF, FR, EF, EOE, DD	185 (47.57)	4 - 5	4.74 (0.44)	-1.79 - 5.90	1.11 (1.33)	20.54
Spence et al., 2011[16]	Canada	community sample (pre-school immunisation program)	English	SR, SE, EUE, FF, FR, EF, EOE, DD	1107 (48.24)	4 - 5	4.93 (0.40)	-2.98 - 6.77	0.41 (1.08)	7.59

Van Deutekom et al. 2016 [17]	Netherlands	Amsterdam Born Children and their Development (ABCD) birth cohort	Dutch and English	EF, FR, SR, SE	3259 (49.80)	5 - 7	5.78 (0.50)	-2.92 - 4.33	0.06 (0.95)	3.22
Viana et al., 2008 [18]	Portugal	clinic- and community-based sample	Portuguese	SR, SE, EUE, FF, FR, EF, EOE, DD	238 (51.26)	2 - 13	7. 87 (2.57)	-2.46 - 6.60	1.32 (1.53)	29.83
**Total**	/	/	/	/						

Abbreviations: *CEBQ* = Child Behaviour Eating Questionnaire, *SR* = Satiety Responsiveness, *SE* = Slowness in Eating, *EUE* = Emotional under-eating, *FF* = Food Fussiness, *FR* = Food Responsiveness, *EF*= Enjoyment of Food, *EOE* = Emotional over-eating, *DD* = Desire to Drink, *N* = number of observations, *SD* = standard deviation, *BMI-SDS* = Body Mass Index Standard Deviation Score(s), *%ob* = percentage of children with obesity, given by BMI SDS > 2

[a] values are presented after removal of observations with missing BMI-SDS and Biologically Implausible Values (BIVs)

7.0 Biologically Implausible Values (BIV) excluded from further analysis

Table SM 7-1: Total number of observations for all appetitive trait subscales, including missing and Biologically Implausible Values (BIV)

N	Total	FR	EOE	EF	DD	SR	SE	FF	EUE
**Normal Weight**[a]	13182	6668	4791	12872	4843	6664	6671	4832	4802
BIV underweight[b]	70	21	20	69	20	21	21	20	20
**Overweight/ Obesity**[c]	2214	1619	1607	2079	1616	1619	1619	1619	1602
BIV overweight[d]	53	26	26	51	26	26	26	26	26
**Missing BMI-SDS**[e]	1889	1223	209	1607	217	1222	1222	216	213

Abbreviations: *N* = number of observations, *BMI-SDS* = Body Mass Index Standard Deviation Score(s), *BIV* = Biologically Implausible Values, *SR* = Satiety Responsiveness, *SE* = Slowness in Eating, *FR* = Food Responsiveness, *EF*= Enjoyment of Food, *DD* = Desire to Drink, *FF* = Food Fussiness, *EOE* = Emotional over-eating, *EUE* = Emotional under-eating

[a] Observations classified into the no obesity group if BMI SDS ≤ 2

[b] Of those classified as having normal weight, observations were considered implausible if BMI SDS ≤ - 3

[c] Observations classified into the obesity group if BMI SDS > 2

[d] Of those classified as having obesity, observations were considered implausible if BMI SDS ≥ 8

[e] Observations for which BMI-SDS could not be determined due to missing data on either height, weight, sex or age

8.0 Average CEBQ subscale scores of included cohorts

Table SM 8-1: Average CEBQ scores of included studies as derived from individual participant data

Cohort	FR	EOE	EF	DD	SR	SE	EUE	FF
Carnell & Wardle et al. (2008)	/	/	4.13 (0.74)	/	/	/	/	/
Cross et al. (2014)	2.31 (0.77)	1.65 (0.61)	3.83 (0.73)	3.40 (1.03)	2.84 (0.62)	2.83 (0.68)	2.66 (0.81)	2.74 (0.78)
De Groot et al. (2017)	2.61 (1.02)	2.33 (1.00)	/	2.61 (0.93)	2.37 (0.76)	2.15 (0.71)	2.20 (0.81)	2.43 (0.82)
Domoff et al. (2015)	2.64 (0.94)	1.80 (0.69)	3.91 (0.80)	3.85 (1.08)	2.91 (0.70)	2.97 (0.77)	2.90 (0.91)	2.78 (0.92)
Dos Passos et al. (2015)	2.21 (0.85)	2.03 (0.89)	3.09 (0.86)	2.62 (1.10)	2.93 (0.79)	2.83 (0.83)	2.63 (0.87)	3.39 (0.95)
Eloranta et al. (2012)	1.71 (0.58)	1.45 (0.49)	3.23 (0.69)	1.80 (0.72)	3.00 (0.62)	2.73 (0.75)	2.34 (0.82)	2.86 (0.77)
Emond et al. (2017)	2.24 (0.71)	1.77 (0.63)	3.71 (0.68)	2.20 (0.75)	2.91 (0.62)	2.61 (0.74)	2.63 (0.81)	2.67 (0.90)
Escobar et al. (2014)	2.28 (0.79)	1.61 (0.60)	3.61 (0.76)	3.04 (1.03)	3.16 (0.67)	3.11 (0.8)0	2.95 (0.89)	2.99 (0.85)
Garcia et al. (2016)	2.20 (0.84)	1.67 (0.61)	3.80 (0.71)	3.32 (0.99)	2.85 (0.65)	2.90 (0.71)	2.76 (0.82)	2.87 (0.83)
Gross et al. (2016)	3.36 (1.06)	2.52 (1.05)	4.12 (0.75)	2.74 (1.08)	2.17 (0.65)	2.13 (0.80)	2.37 (0.79)	2.84 (0.98)
Hardman et al. (2016)	2.47 (0.86)	1.71 (0.73)	4.06 (0.68)	2.30 (0.88)	2.65 (0.74)	2.71 (0.90)	2.51 (0.86)	2.53 (0.99)
Haycraft et al. (2011)	2.17 (0.64)	1.68 (0.55)	3.76 (0.68)	2.35 (0.91)	2.99 (0.66)	3.02 (0.80)	2.72 (0.89)	2.78 (0.82)
Hayes et al. (2016)	3.65 (0.80)	2.84 (0.79)	4.26 (0.65)	2.84 (0.91)	2.20 (0.56)	2.13 (0.71)	2.65 (0.65)	2.93 (0.94)
McCarthy et al. (2015)	/	1.51 (0.50)	3.94 (0.73)	2.80 (1.00)	/	/	2.89 (0.93)	2.55 (0.81)
Spence et al. (2011)	2.22 (0.65)	1.66 (0.56)	3.54 (0.70)	2.78 (0.93)	3.19 (0.60)	3.06 (0.73)	2.80 (0.83)	3.04 (0.83)
Van Deutekom et al. (2016)	1.89 (0.51)	/	2.53 (0.50)	/	2.37 (0.50)	2.47 (0.58)	/	/
Viana et al. (2008)	2.36 (1.11)	2.24 (0.91)	3.14 (1.04)	2.60 (0.97)	2.92 (0.62)	2.80 (0.72)	2.82 (0.87)	2.82 (0.82)
Overall	2.28 (0.89)	1.83 (0.79)	3.64 (0.94)	2.88 (1.12)	2.67 (0.70)	2.66 (0.76)	2.72 (0.88)	2.84 (0.89)

Abbreviations: *FR* = Food Responsiveness, *EOE* = Emotional over-eating, *EF*= Enjoyment of Food, *DD* = Desire to Drink, *SR* = Satiety Responsiveness, *SE* = Slowness in Eating, *EUE* = Emotional under-eating, *FF* = Food Fussiness

9.0 Number of missing values for CEBQ subscales

Table SM 9-1: Number of missing CEBQ scores of included studies as derived from individual participant data

Cohort	FR	EOE	EF	DD	SR	SE	EUE	FF
Carnell & Wardle et al. (2008)	/	/	3	/	/	/	/	/
Cross et al. (2014)	0	0	0	0	0	0	0	0
De Groot et al. (2017)	3	3	/	3	3	3	3	3
Domoff et al. (2015)	6	42	11	15	9	8	35	7
Dos Passos et al. (2015)	0	0	0	0	0	0	0	0
Eloranta et al. (2012)	3	6	2	1	6	1	1	4
Emond et al. (2017)	0	0	0	0	0	0	0	0
Escobar et al. (2014)	0	0	0	0	0	0	0	0
Garcia et al. (2016)	0	0	0	0	0	0	0	0
Gross et al. (2016)	92	97	90	92	90	91	100	90
Hardman et al. (2016)	13	13	13	13	13	13	13	13
Haycraft et al. (2011)	0	1	0	0	0	0	0	0
Hayes et al. (2016)	0	0	0	0	0	0	0	0
McCarthy et al. (2015)	/	0	0	0	/	/	0	0
Spence et al. (2011)	46	59	38	36	46	47	63	51
Van Deutekom et al. (2016)	240	/	241	/	240	237	/	/
Viana et al. (2008)	0	0	0	0	0	0	0	0
Overall	403	221	398	160	407	400	215	168

Abbreviations: *FR* = Food Responsiveness, *EOE* = Emotional over-eating, *EF*= Enjoyment of Food, *DD* = Desire to Drink, *SR* = Satiety Responsiveness, *SE* = Slowness in Eating, *EUE* = Emotional under-eating, *FF* = Food Fussiness

10.0 Number of children in BMI-SDS groups

Table SM 10-1: Number of children in BMI-SDS groups by CEBQ subscale and cohort as derived from individual participant data

Cohort	BMI-SDS group	FR	EOE	EF	DD	SR	SE	EUE	FF
Carnell & Wardle et al. (2008)	No obesity[a]	/	/	5075	/	/	/	/	/
	Obesity[b]	/	/	363	/	/	/	/	/
Cross et al. (2014)	No obesity[a]	162	162	162	162	162	162	162	162
	Obesity[b]	47	47	47	47	47	47	47	47
De Groot et al. (2017)	No obesity[a]	18	18	/	18	18	18	18	18
	Obesity[b]	23	23	/	23	23	23	23	23
Domoff et al. (2015)	No obesity[a]	853	825	850	847	852	853	832	854
	Obesity[b]	130	122	128	127	128	128	122	128
Dos Passos et al. (2015)	No obesity[a]	249	249	249	249	249	249	249	249
	Obesity[b]	82	82	82	82	82	82	82	82
Eloranta et al. (2012)	No obesity[a]	442	439	443	444	439	444	444	441
	Obesity[b]	33	33	33	33	33	33	33	33
Emond et al. (2017)	No obesity[a]	153	153	153	153	153	153	153	153
	Obesity[b]	25	25	25	25	25	25	25	25
Escobar et al. (2014)	No obesity[a]	325	325	325	325	325	325	325	325
	Obesity[b]	21	21	21	21	21	21	21	21
Garcia et al. (2016)	No obesity[a]	147	147	147	147	147	147	147	147
	Obesity[b]	38	38	38	38	38	38	38	38
Gross et al. (2016)	No obesity[a]	26	25	26	26	26	25	26	26
	Obesity[b]	704	700	706	704	706	706	696	706

Cohort	BMI-SDS group	FR	EOE	EF	DD	SR	SE	EUE	FF
Hardman et al. (2016)	No obesity[a]	54	54	54	54	54	54	54	54
	Obesity[b]	12	12	12	12	12	12	12	12
Haycraft et al. (2011)	No obesity[a]	119	118	119	119	119	119	119	119
	Obesity[b]	18	18	18	18	18	18	18	18
Hayes et al. (2016)	No obesity[a]	19	19	19	19	19	19	19	19
	Obesity[b]	222	222	222	222	222	222	222	222
McCarthy et al. (2015)	No obesity[a]	/	1097	1097	1097	/	/	1097	1097
	Obesity[b]	/	92	92	92	/	/	92	92
Spence et al. (2011)	No obesity[a]	984	972	989	995	984	983	969	980
	Obesity[b]	77	76	80	76	77	77	75	76
Van Deutekom et al. (2016)	No obesity[a]	2928	/	2927	/	2928	2931	/	/
	Obesity[b]	91	/	91	/	91	91	/	/
Viana et al. (2008)	No obesity[a]	168	168	168	168	168	168	168	168
	Obesity[b]	70	70	70	70	70	70	70	70

Abbreviations: *FR* = Food Responsiveness, *EOE* = Emotional over-eating, *EF*= Enjoyment of Food, *DD* = Desire to Drink, *SR* = Satiety Responsiveness, *SE* = Slowness in Eating, *EUE* = Emotional under-eating, *FF* = Food Fussiness

[a] Observations classified into the no obesity group if BMI SDS ≤ 2

[b] Observations classified into the obesity group if BMI SDS > 2

11.0 Quintile scores on CEBQ subscales

Table SM 11-1: Quintile scores for the CEBQ subscale Food Responsiveness

	Males			Females		
Quintile	Early Childhood	Middle Childhood	Late Childhood	Early Childhood	Middle Childhood	Late Childhood
0%	1.00	1.00	1.20	1.00	1.00	1.00
20%	1.50	1.56	2.20	1.50	1.60	2.20
40%	2.00	2.00	3.00	2.00	2.00	2.80
60%	2.20	2.80	3.60	2.25	2.75	3.60
80%	2.60	3.80	4.40	2.60	3.80	4.20
100%	5.00	5.00	5.00	5.00	5.00	5.00

Table SM 11-2: Quintile scores for the CEBQ subscale Emotional Overeating

	Males			Females		
Quintile	Early Childhood	Middle Childhood	Late Childhood	Early Childhood	Middle Childhood	Late Childhood
0%	1.00	1.00	1.00	1.00	1.00	1.00
20%	1.00	1.25	1.75	1.00	1.25	2.00
40%	1.50	1.75	2.25	1.25	1.75	2.25
60%	1.75	2.25	3.00	1.75	2.25	3.00
80%	2.00	3.00	3.75	2.25	3.00	3.50
100%	5.00	5.00	5.00	5.00	5.00	5.00

Table SM 11-3: Quintile scores for the CEBQ subscale Enjoyment of Food

	Males			Females		
Quintile	Early Childhood	Middle Childhood	Late Childhood	Early Childhood	Middle Childhood	Late Childhood
0%	1.00	1.00	2.00	1.00	1.00	1.25
20%	2.40	3.25	3.50	2.50	3.25	3.50
40%	2.80	4.00	4.00	2.88	4.00	4.00
60%	3.50	4.25	4.50	3.50	4.25	4.50
80%	4.00	4.75	4.75	4.00	4.75	4.75
100%	5.00	5.00	5.00	5.00	5.00	5.00

Table SM 11-4: Quintile scores for the CEBQ subscale Desire to Drink

	Males			Females		
Quintile	Early Childhood	Middle Childhood	Late Childhood	Early Childhood	Middle Childhood	Late Childhood
0%	1.00	1.00	1.00	1.00	1.00	1.00
20%	2.00	1.67	1.94	2.00	1.33	1.33
40%	2.67	2.00	2.33	2.67	2.00	2.00
60%	3.33	2.33	3.00	3.33	2.33	2.50
80%	4.33	3.33	3.67	4.00	3.00	3.30
100%	5.00	5.00	5.00	5.00	5.00	5.00

Table SM 11-5: Quintile scores for the CEBQ subscale Satiety Responsiveness

	Males			Females		
Quintile	Early Childhood	Middle Childhood	Late Childhood	Early Childhood	Middle Childhood	Late Childhood
0%	1.00	1.00	1.00	1.00	1.00	1.00
20%	2.00	2.00	1.60	2.20	2.00	1.60
40%	2.40	2.40	2.00	2.60	2.40	2.00
60%	2.80	2.80	2.20	2.80	2.80	2.40
80%	3.20	3.20	2.80	3.25	3.40	2.80
100%	5.00	4.60	4.00	5.00	5.00	4.40

Table SM 11-6: Quintile scores for the CEBQ subscale Slowness in Eating

	Males			Females		
Quintile	Early Childhood	Middle Childhood	Late Childhood	Early Childhood	Middle Childhood	Late Childhood
0%	1.00	1.00	1.00	1.00	1.00	1.00
20%	2.00	1.75	1.25	2.25	2.00	1.50
40%	2.50	2.00	1.75	2.50	2.25	2.00
60%	2.75	2.50	2.00	3.00	2.75	2.50
80%	3.25	3.00	2.50	3.25	3.25	3.00
100%	5.00	5.00	4.00	5.00	4.75	5.00

Table SM 11-7: Quintile scores for the CEBQ subscale Food Fussiness

	Males			Females		
Quintile	Early Childhood	Middle Childhood	Late Childhood	Early Childhood	Middle Childhood	Late Childhood
0%	1.00	1.00	1.00	1.00	1.00	1.00
20%	2.17	2.00	1.83	2.00	2.00	1.70
40%	2.67	2.67	2.50	2.50	2.50	2.33
60%	3.00	3.17	2.83	3.00	3.00	2.67
80%	3.67	3.73	3.50	3.50	3.67	3.17
100%	5.00	5.00	4.83	5.00	5.00	5.00

Table SM 11-8: Quintile scores for the CEBQ subscale Emotional Undereating

	Males			Females		
Quintile	Early Childhood	Middle Childhood	Late Childhood	Early Childhood	Middle Childhood	Late Childhood
0%	1.00	1.00	1.00	1.00	1.00	1.00
20%	2.00	1.75	1.50	2.00	1.75	2.00
40%	2.75	2.25	2.00	2.50	2.25	2.30
60%	3.00	2.75	2.50	3.00	2.75	2.50
80%	3.50	3.25	2.88	3.50	3.25	3.00
100%	5.00	5.00	4.00	5.00	5.00	4.50

12.0 Ridgeline plots of the distribution of CEBQ subscale scores across BMI-SDS groups within Cohorts

Figure SM 12-1 Distribution of Food Responsiveness (FR) scores across BMI-SDS Groups within Cohorts

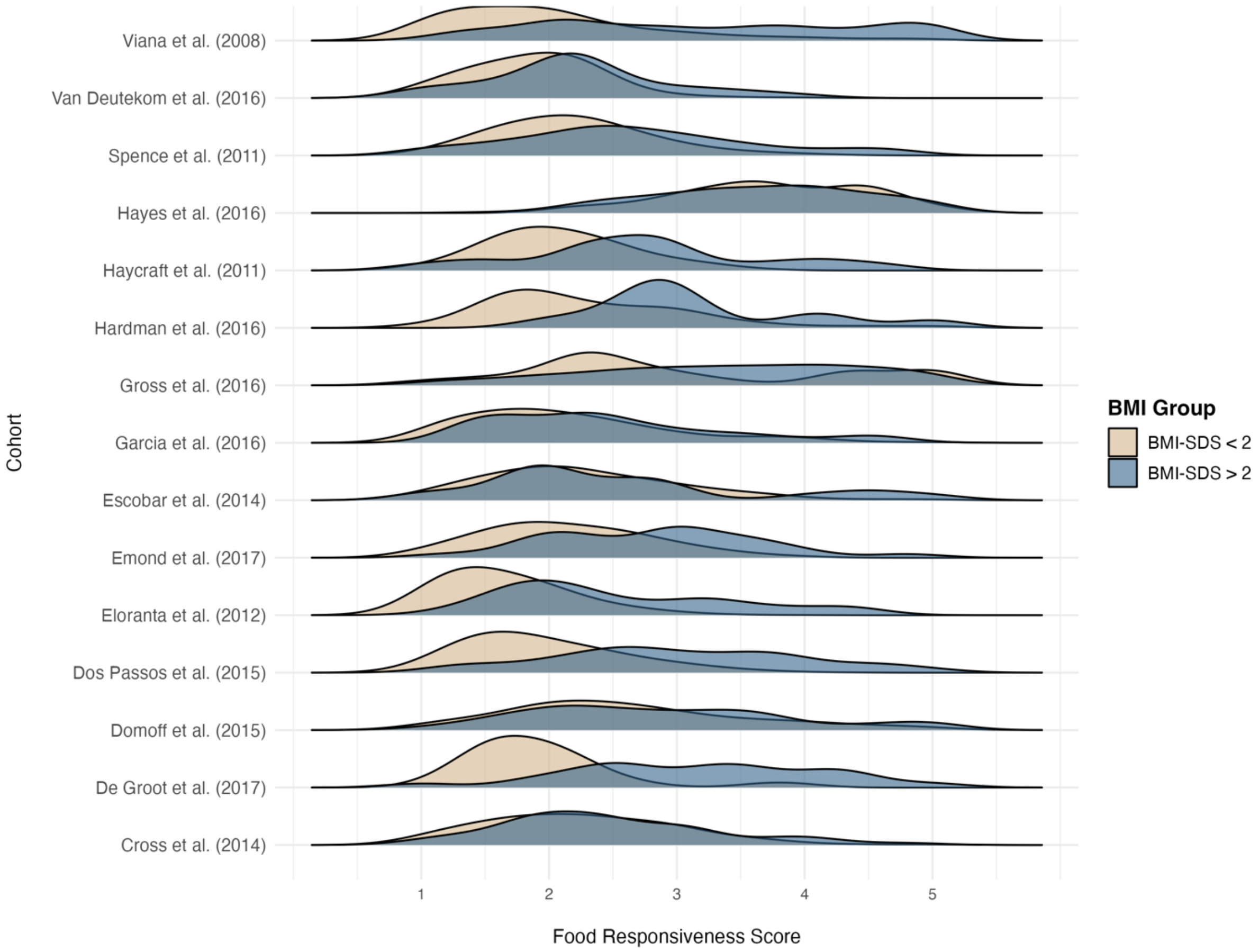

Figure SM 12-2 Distribution of Emotional Overeating (EOE) scores across BMI-SDS Groups within Cohorts

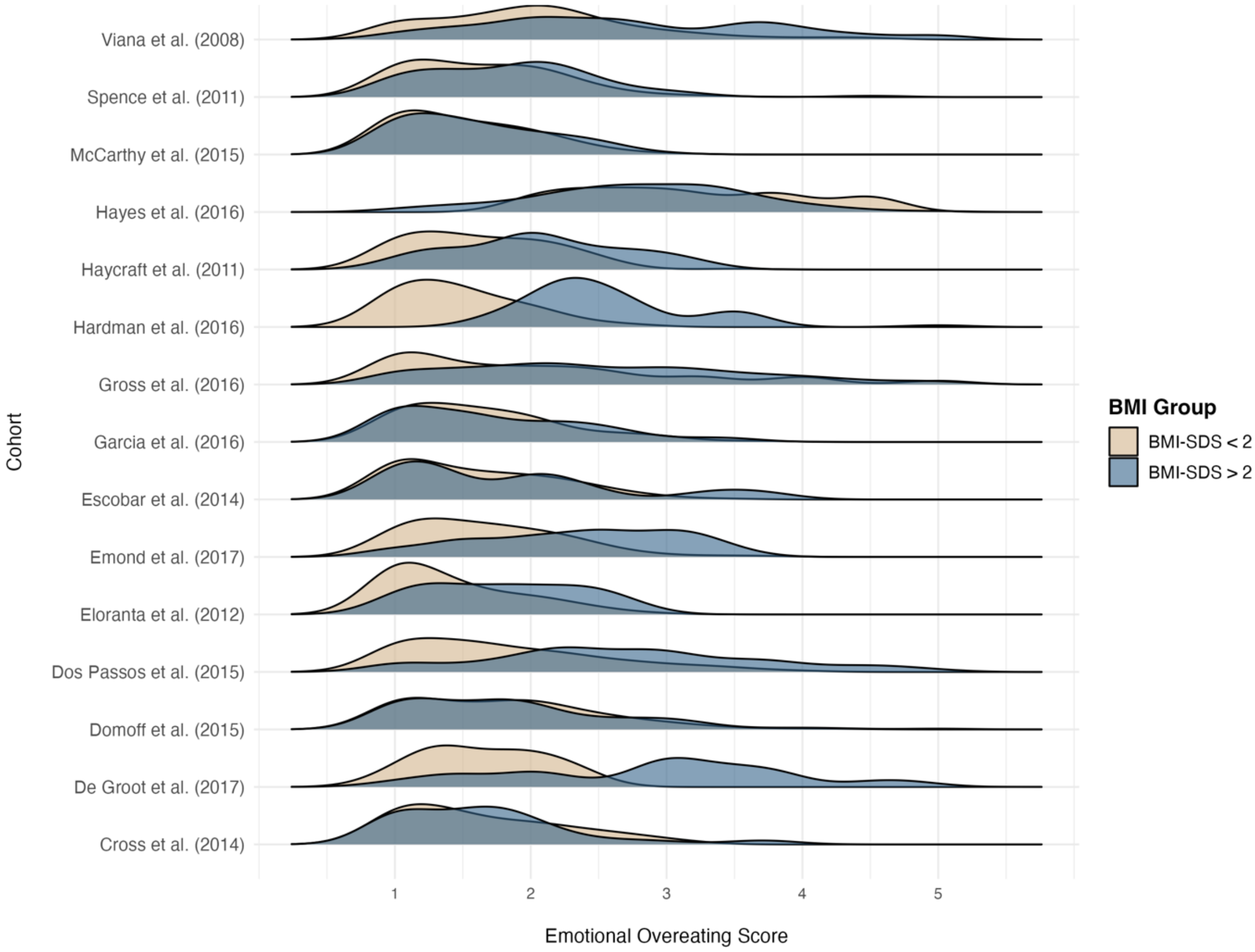

Figure SM 12-3 Distribution of Enjoyment of Food (EF) scores across BMI-SDS Groups within Cohorts

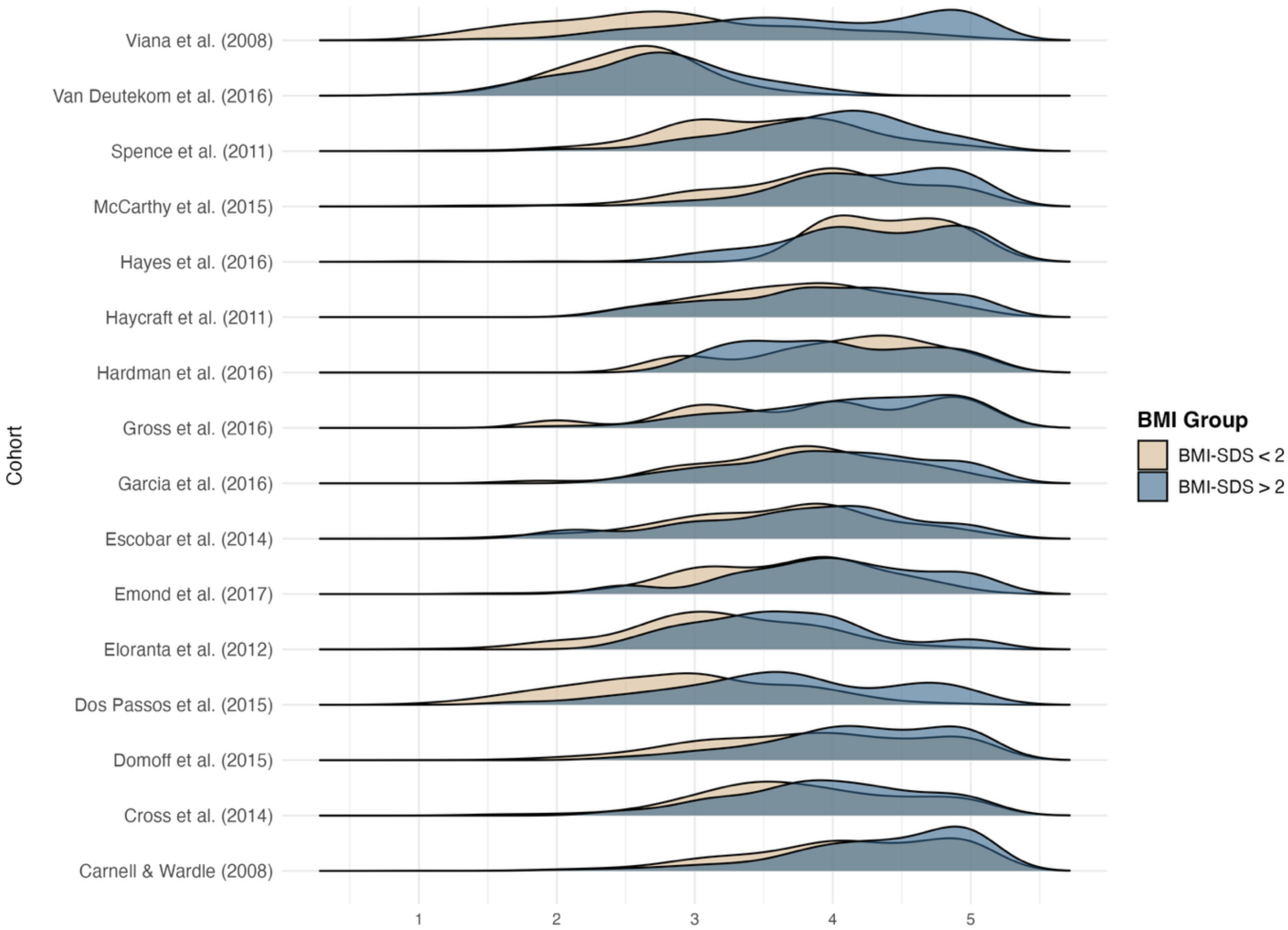

Figure SM 12-4 Distribution of Desire to Drink (DD) scores across BMI-SDS Groups within Cohorts

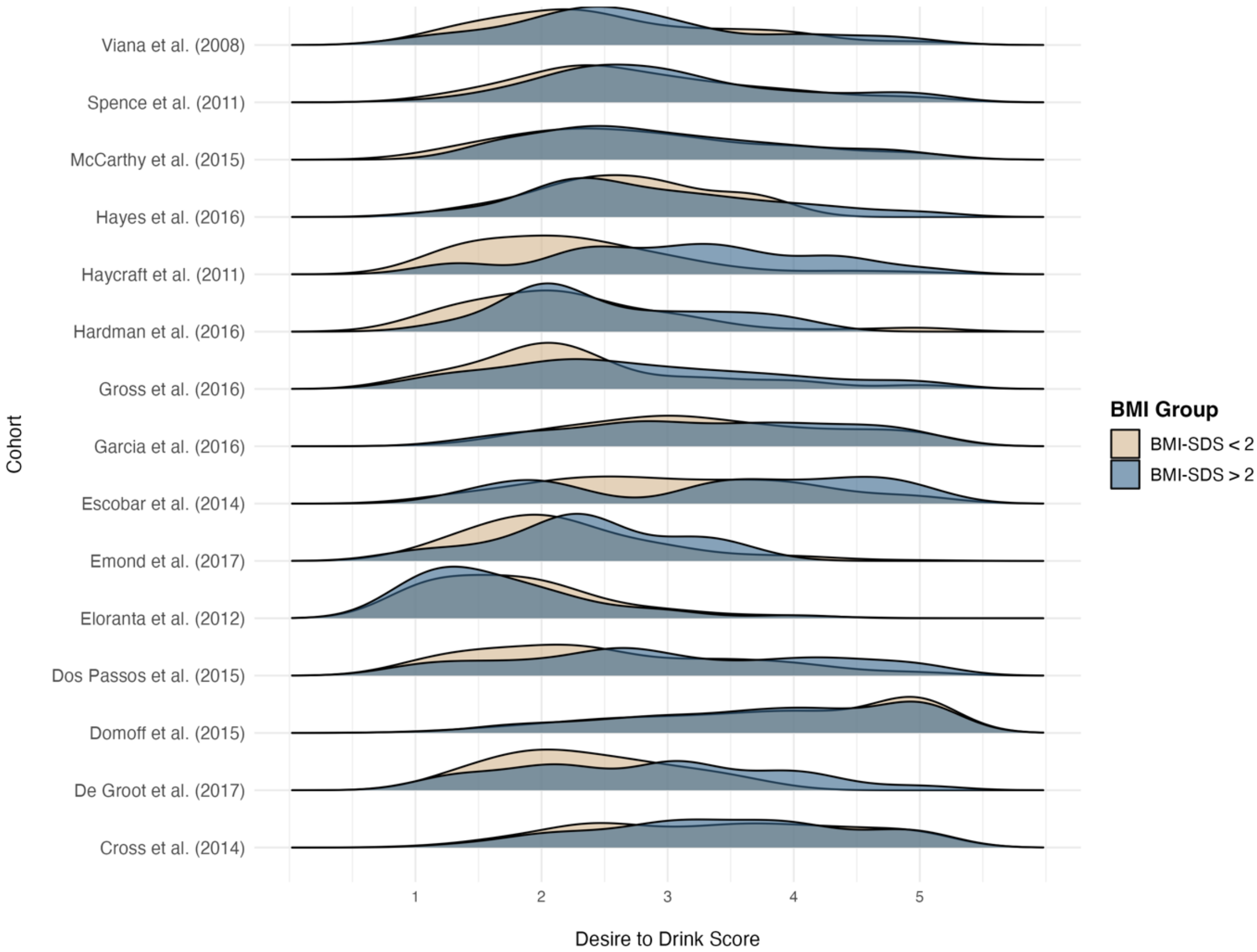

Figure SM 12-5 Distribution of Satiety Responsiveness (SR) scores across BMI-SDS Groups within Cohorts

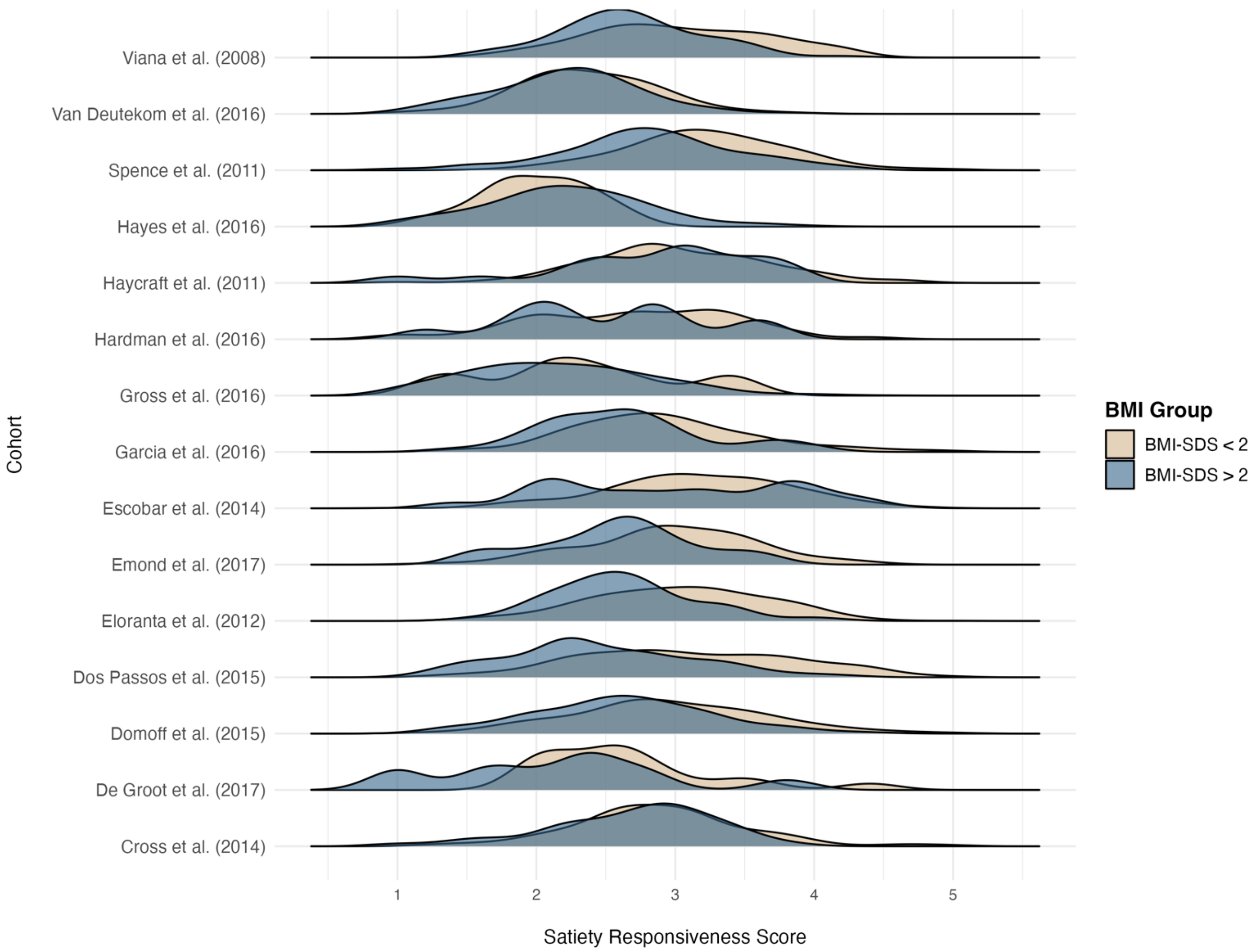

Figure SM 12-6 Distribution of Slowness in Eating (SE) scores across BMI-SDS Groups within Cohorts

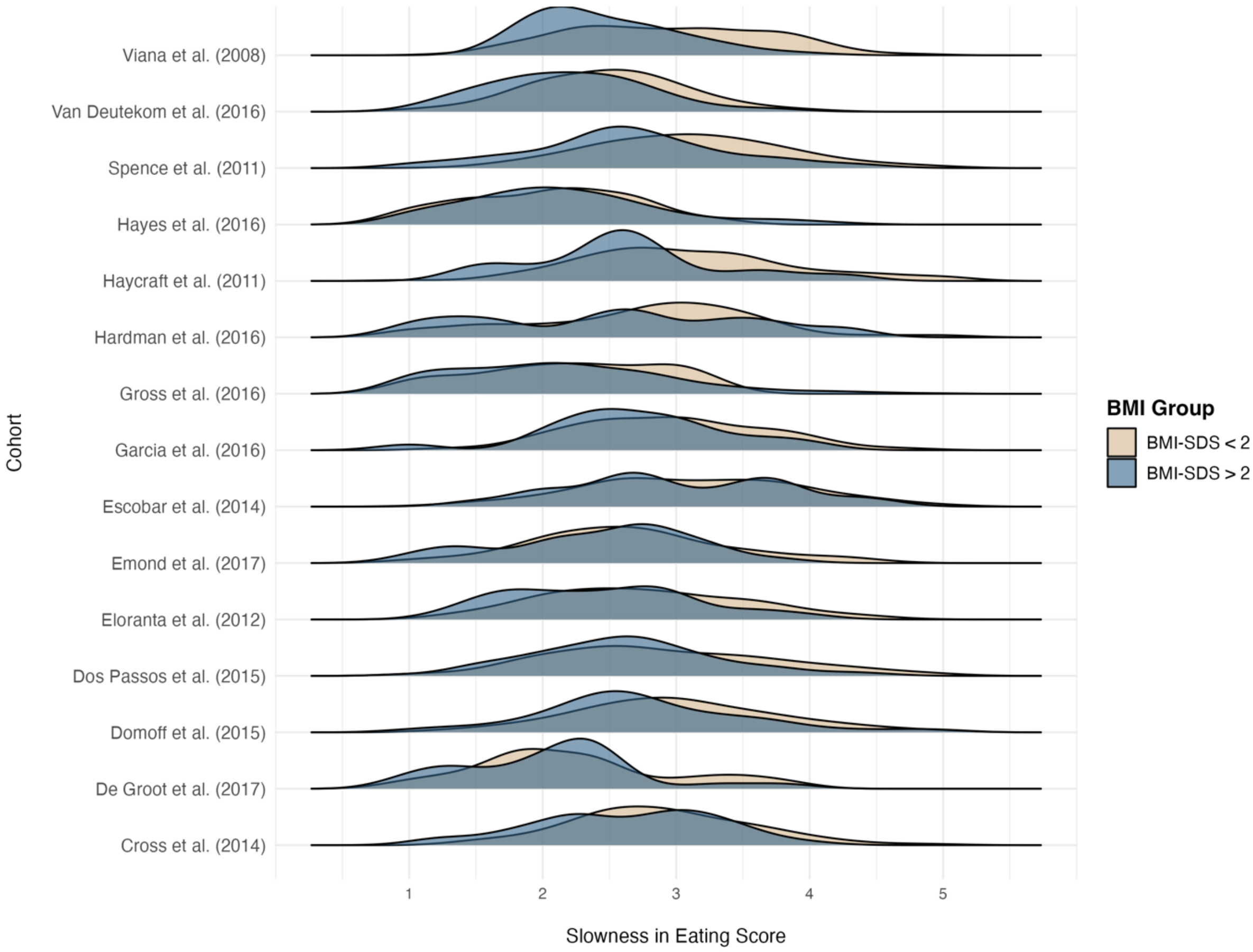

Figure SM 12-7 Distribution of Food Fussiness (FF) scores across BMI-SDS Groups within Cohorts

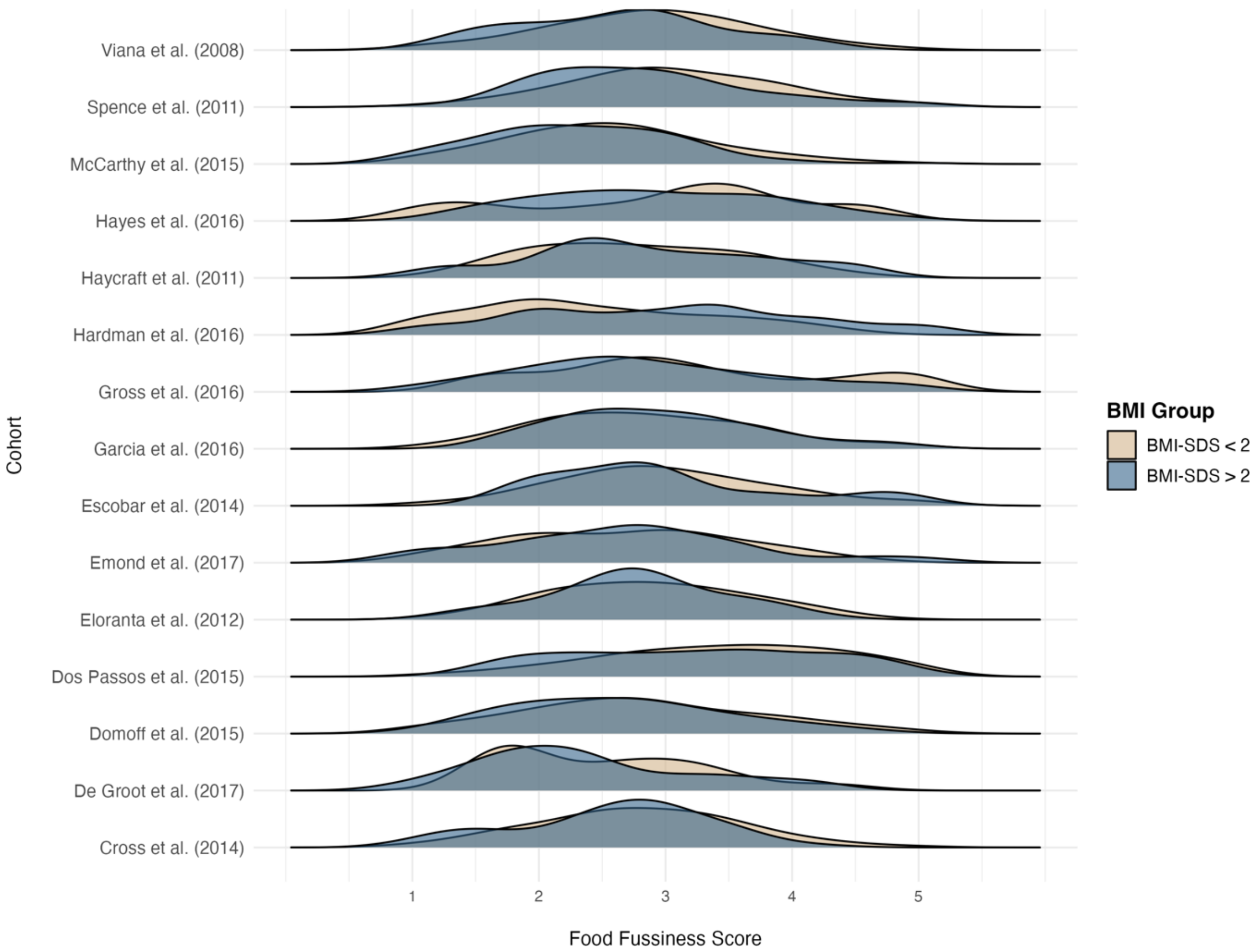

Figure SM 12-8 Distribution of Emotional Undereating (EUE) scores across BMI-SDS Groups within Cohorts

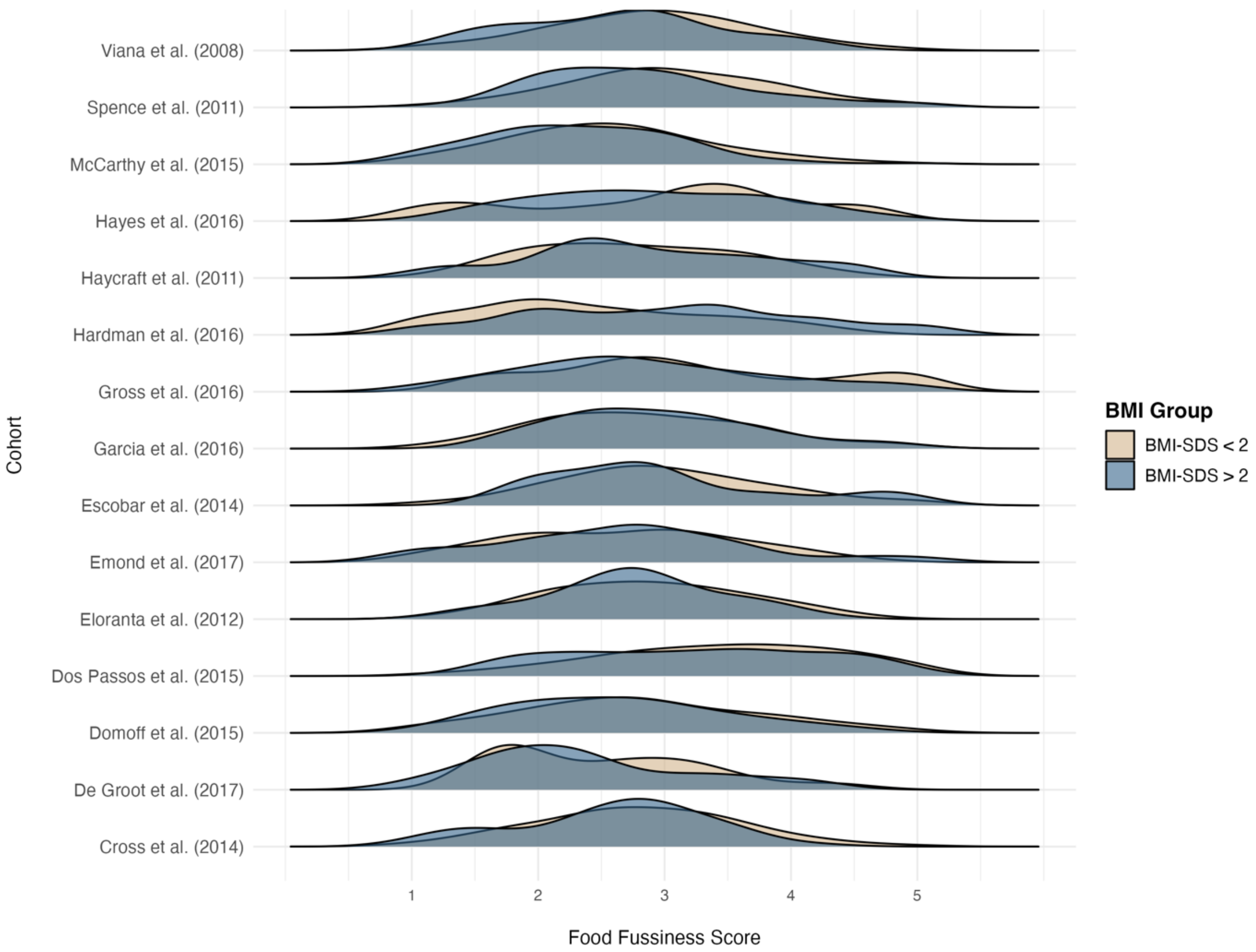

13.0 Receiver Operating Curves (ROC) of CEBQ subscale scores across cohorts

Figure SM 13-1 Receiver Operating Curve (ROC) of Food Responsiveness (FR) scores across Cohorts

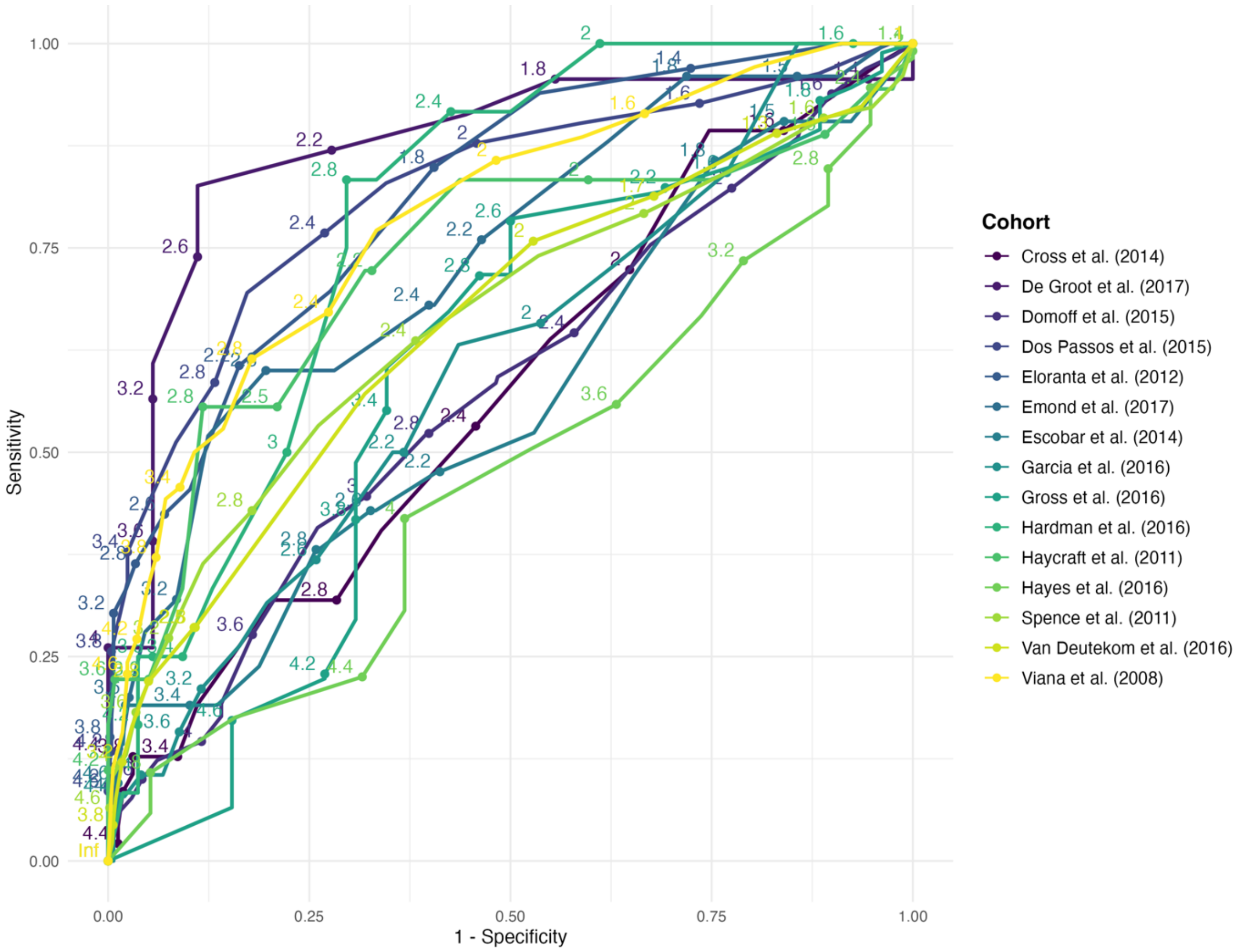

Figure SM 13-2 Receiver Operating Curve (ROC) of Emotional Overeating (EOE) scores across Cohorts

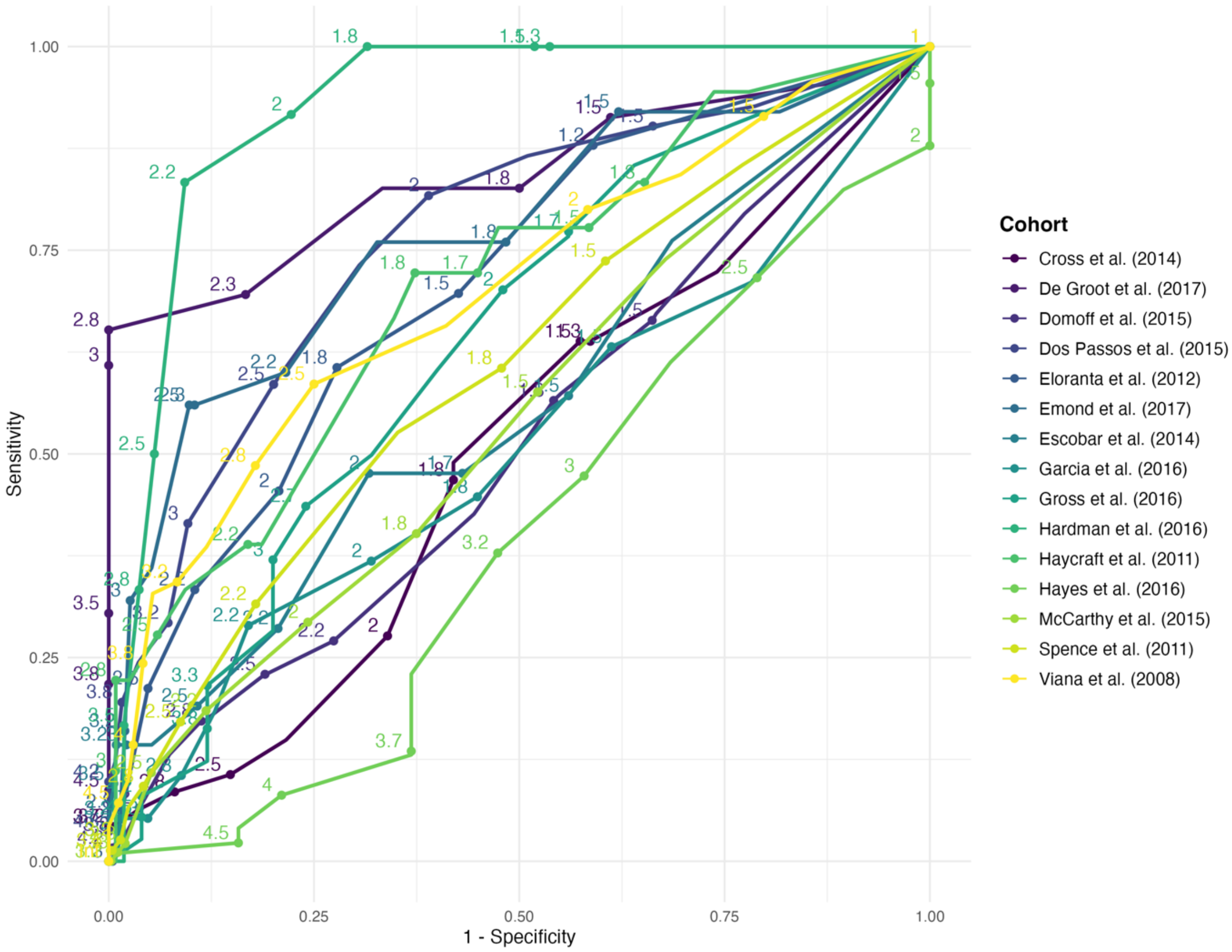

Figure SM 13-3 Receiver Operating Curve (ROC) of Enjoyment of Food (EF) scores across Cohorts

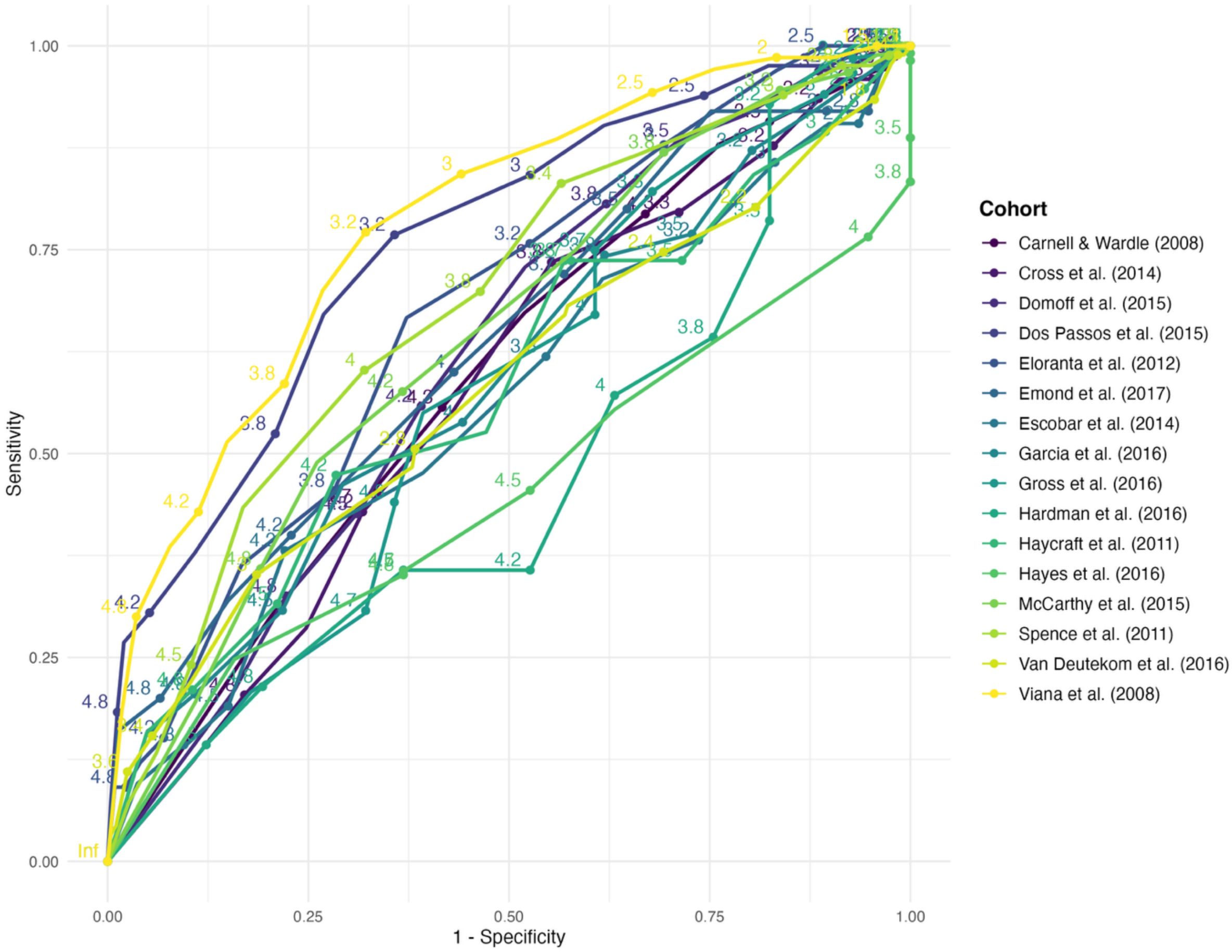

Figure SM 13-4 Receiver Operating Curve (ROC) of Desire to Drink (DD) scores across Cohorts

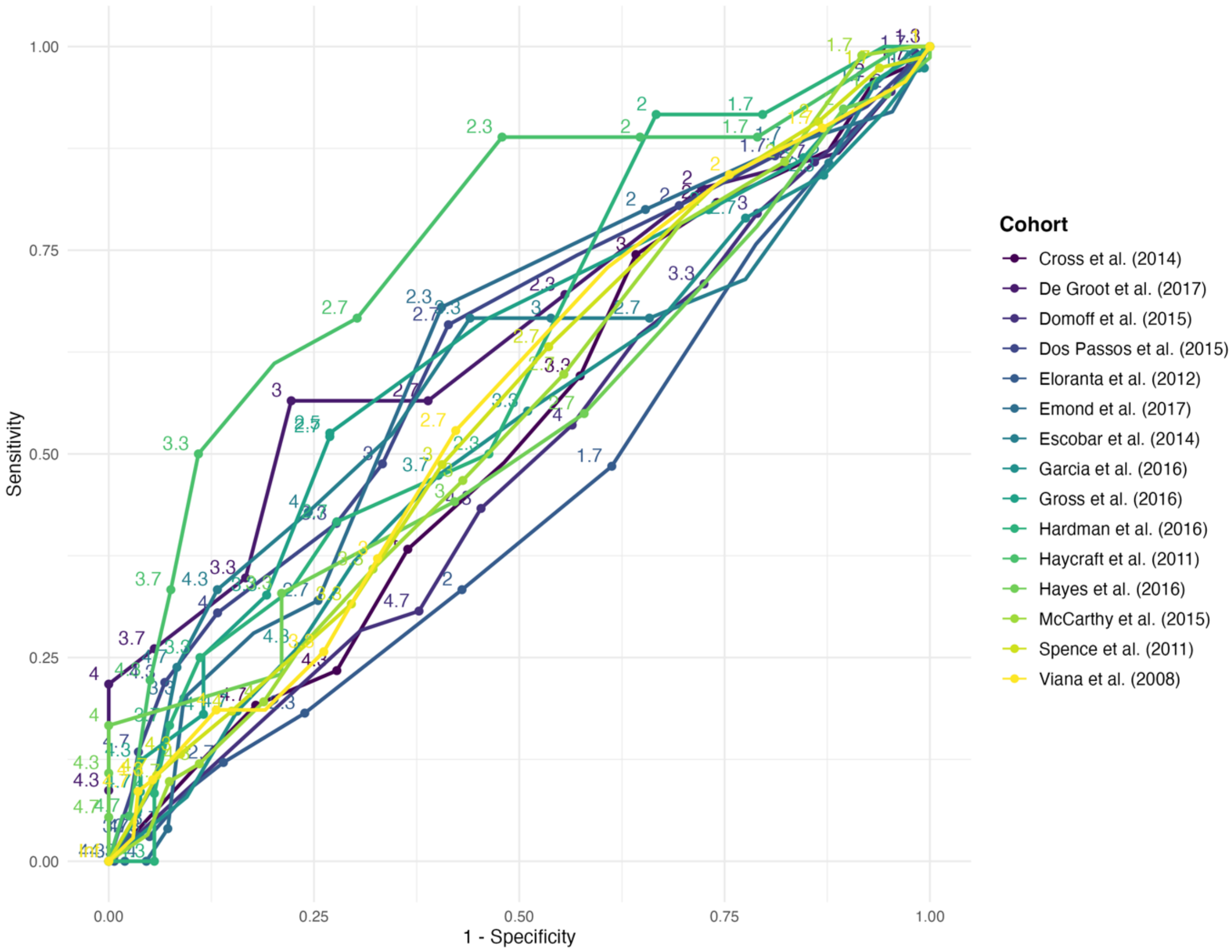

Figure SM 13-5 Receiver Operating Curve (ROC) of Satiety Responsiveness (SR) scores across Cohorts

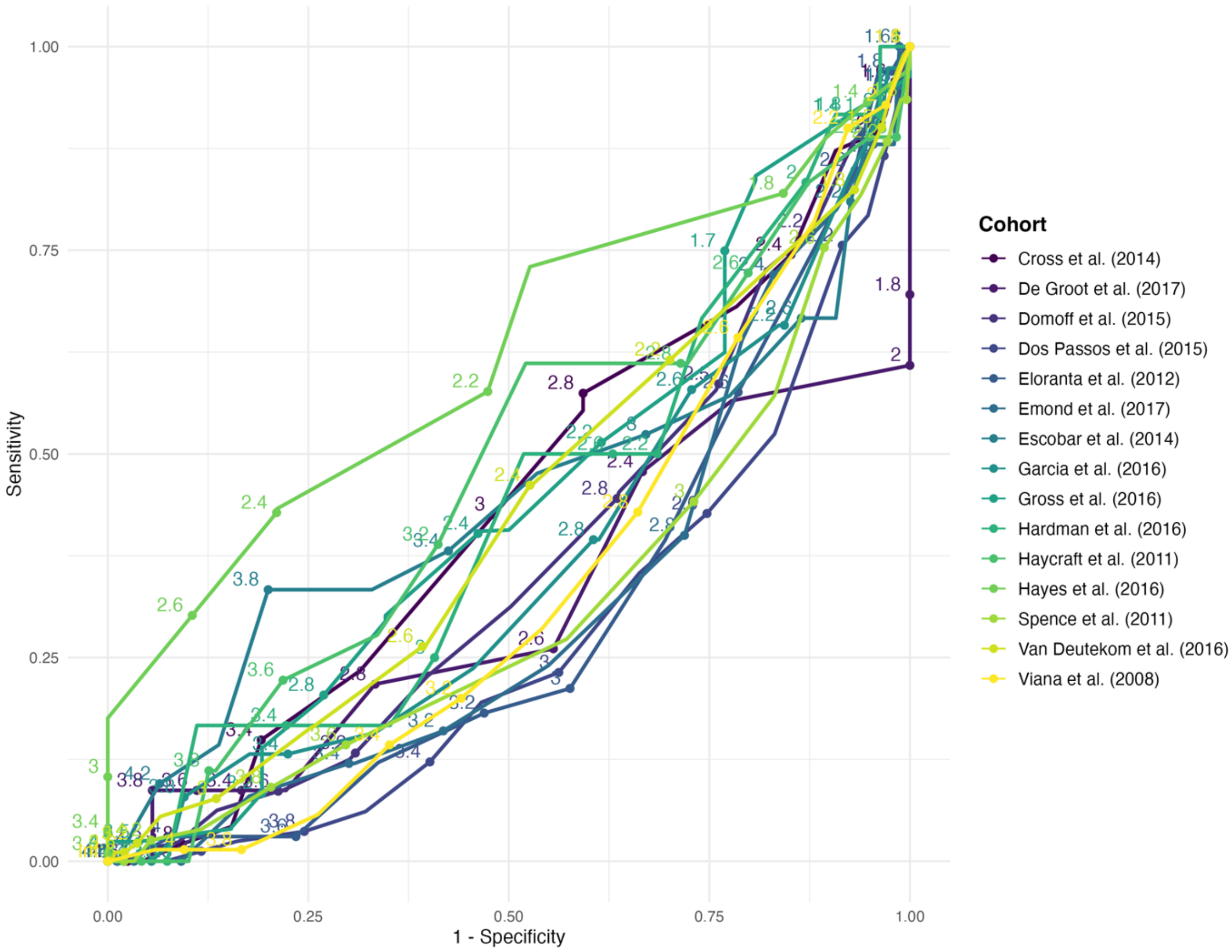

Figure SM 13-6 Receiver Operating Curve (ROC) of Slowness in Eating (SE) scores across Cohorts

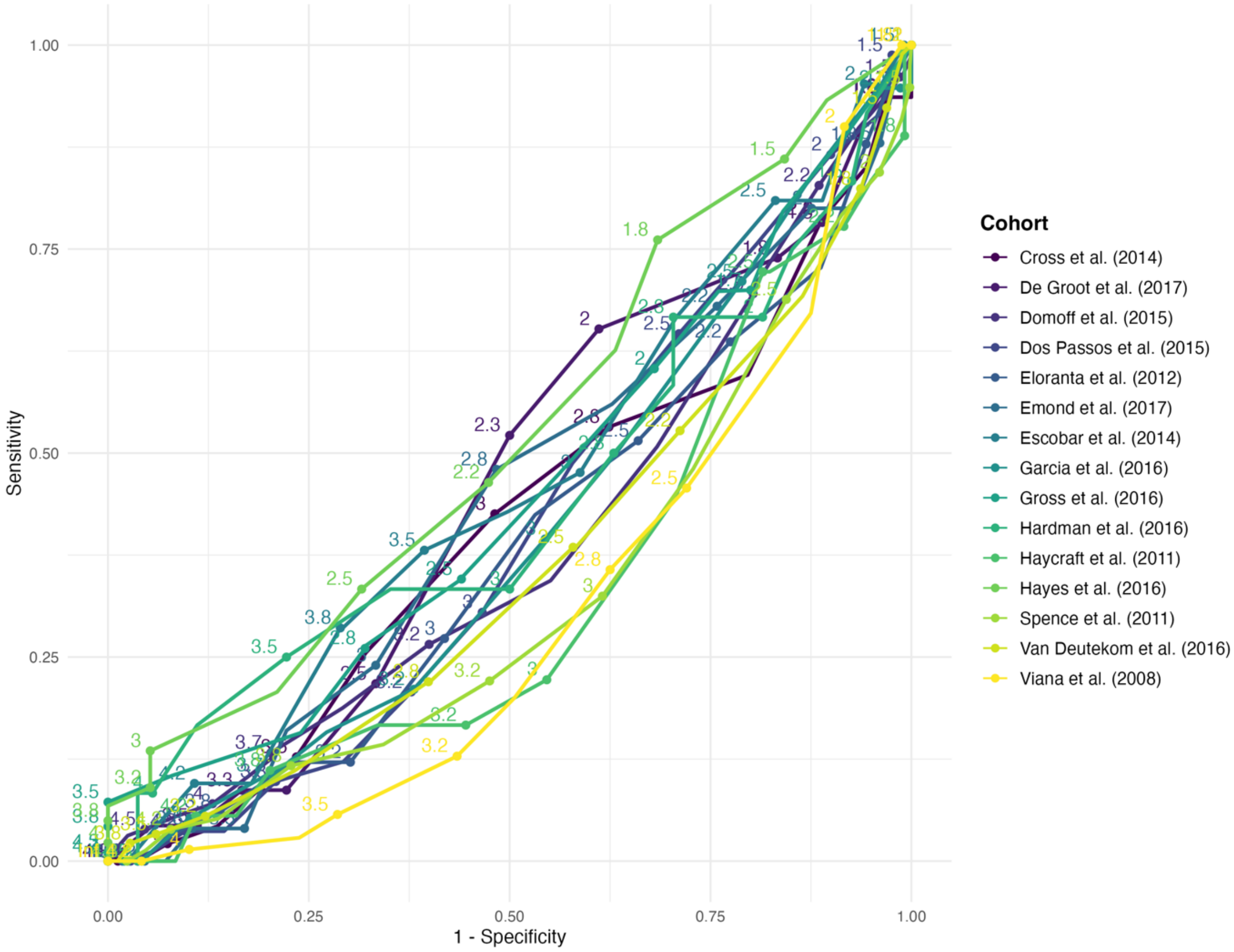

Figure SM 13-7 Receiver Operating Curve (ROC) of Food Fussiness (FF) scores across Cohorts

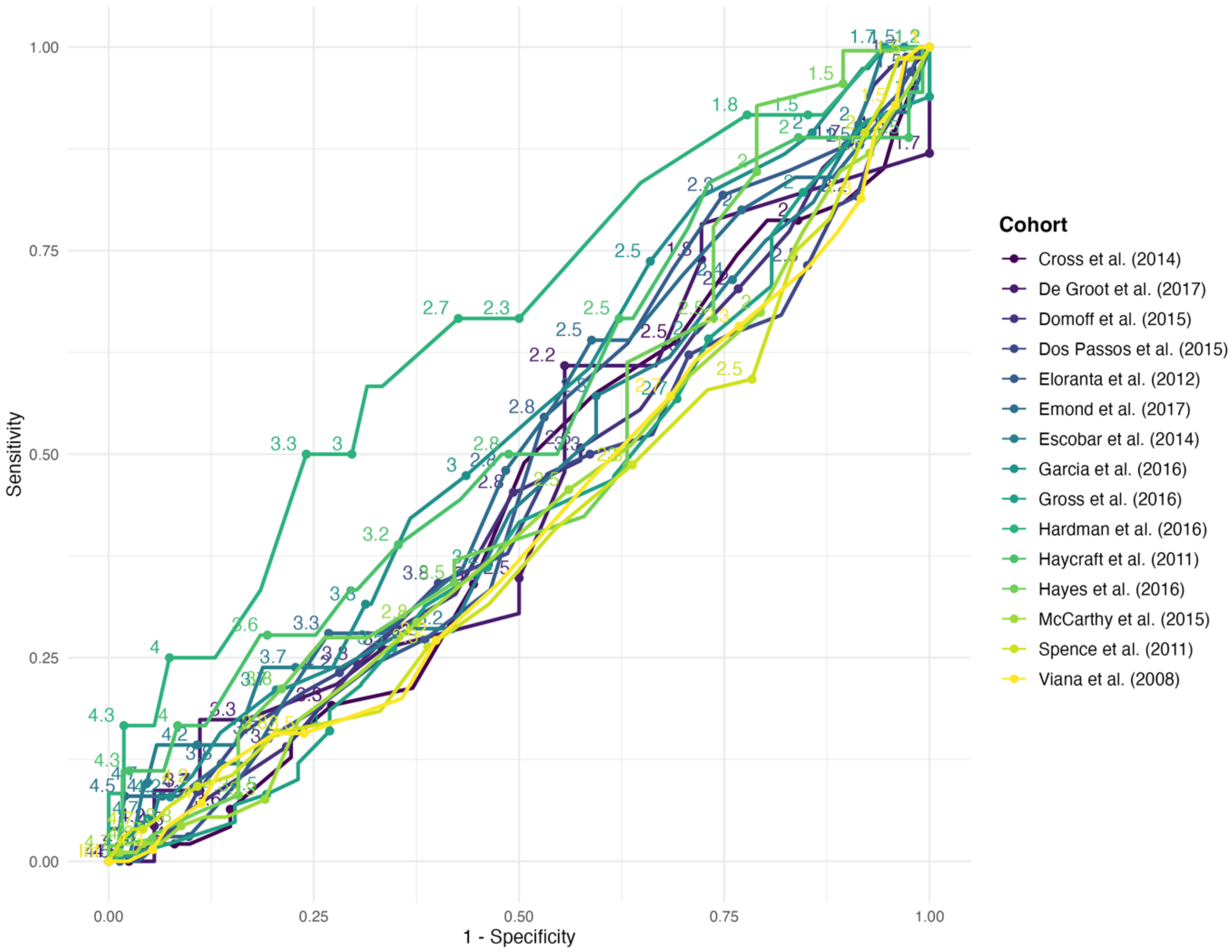

Figure SM 13-8 Receiver Operating Curve (ROC) of Emotional Undereating (EUE) scores across Cohorts

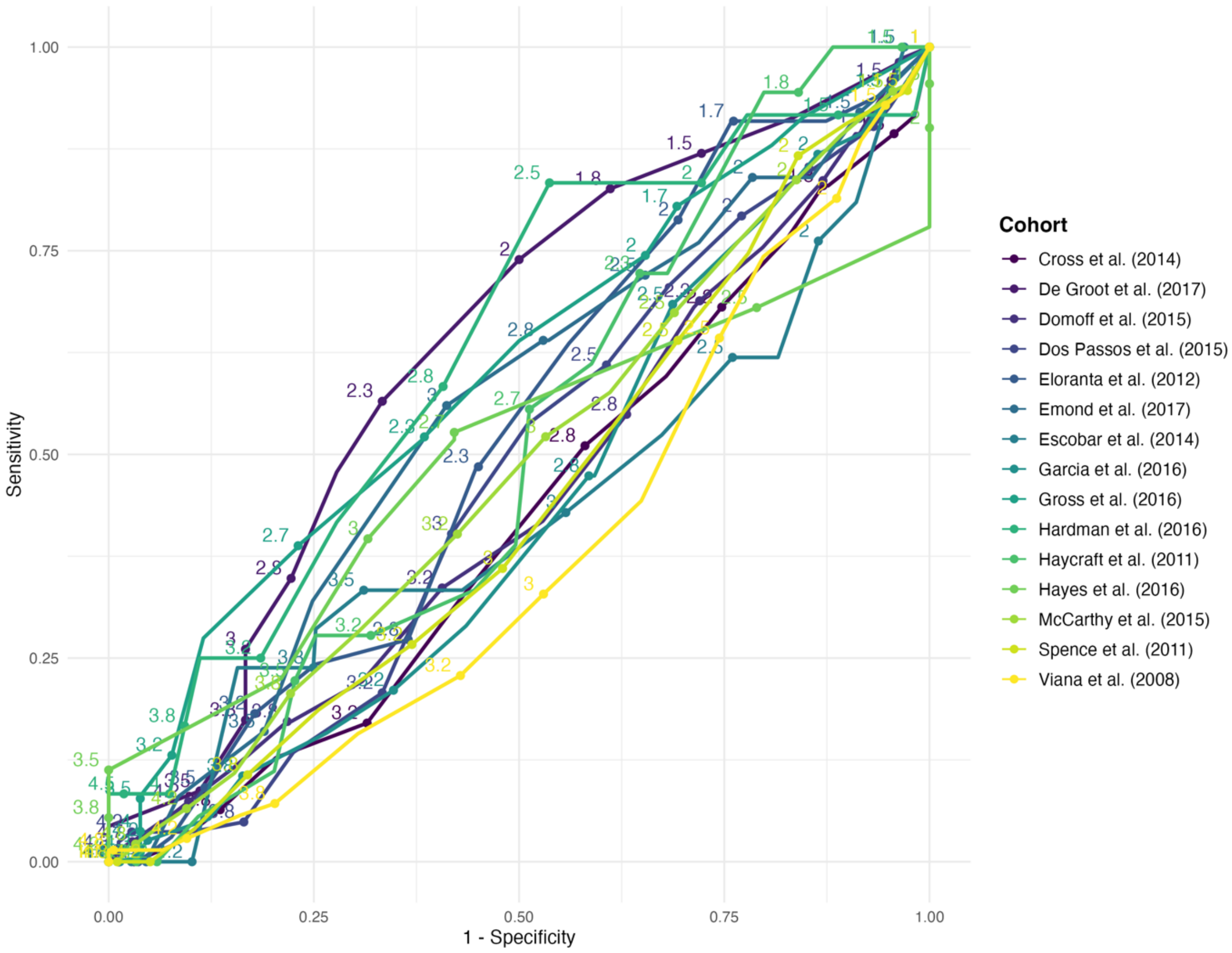

14.0 Forest Plots of AUCs

Figure SM 14-1 Forest Plot of Food Responsiveness (FR) Scores across Cohorts

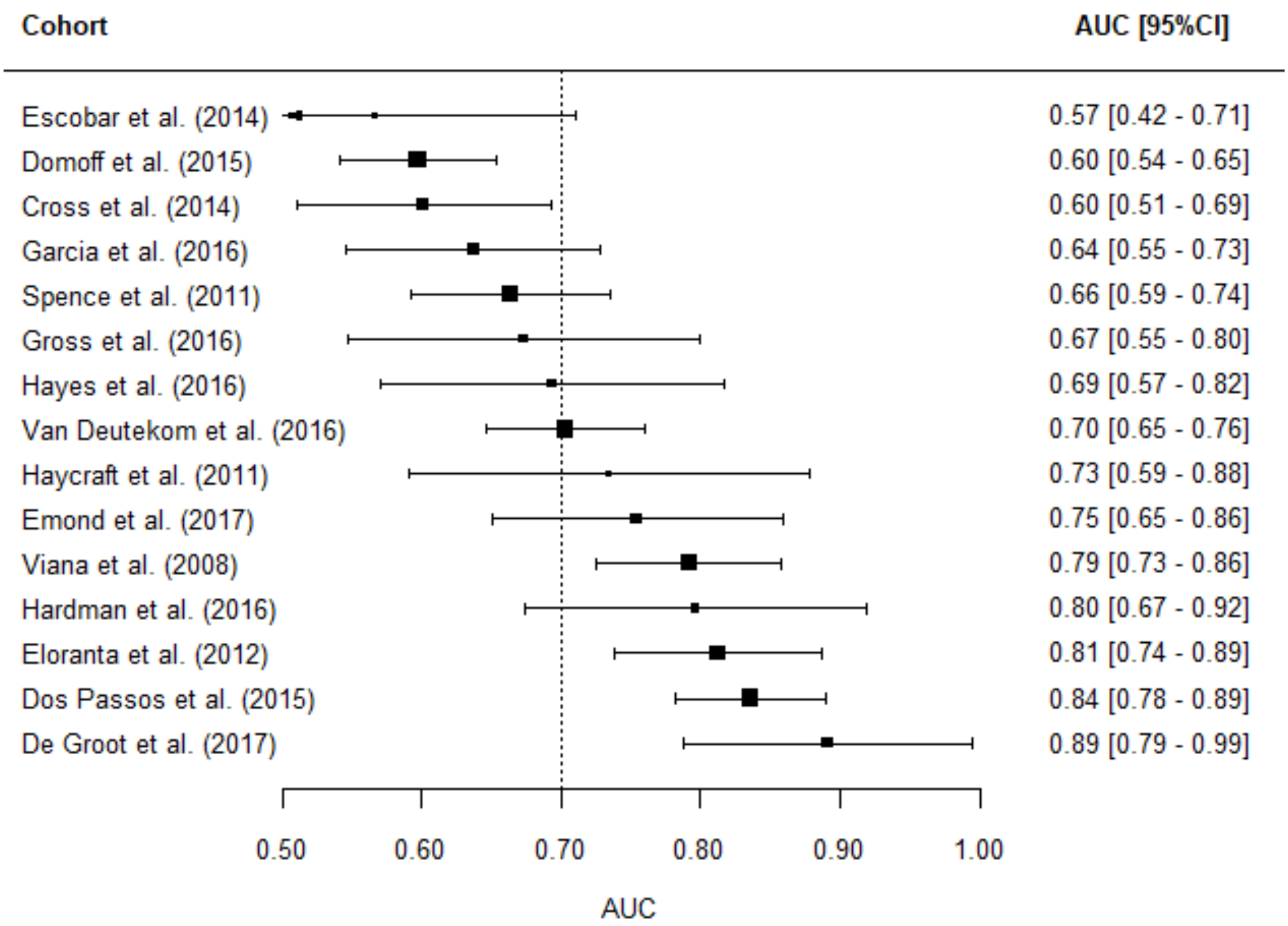

Figure SM 14-2 Forest Plot of Emotional Overeating (EOE) Scores across Cohorts

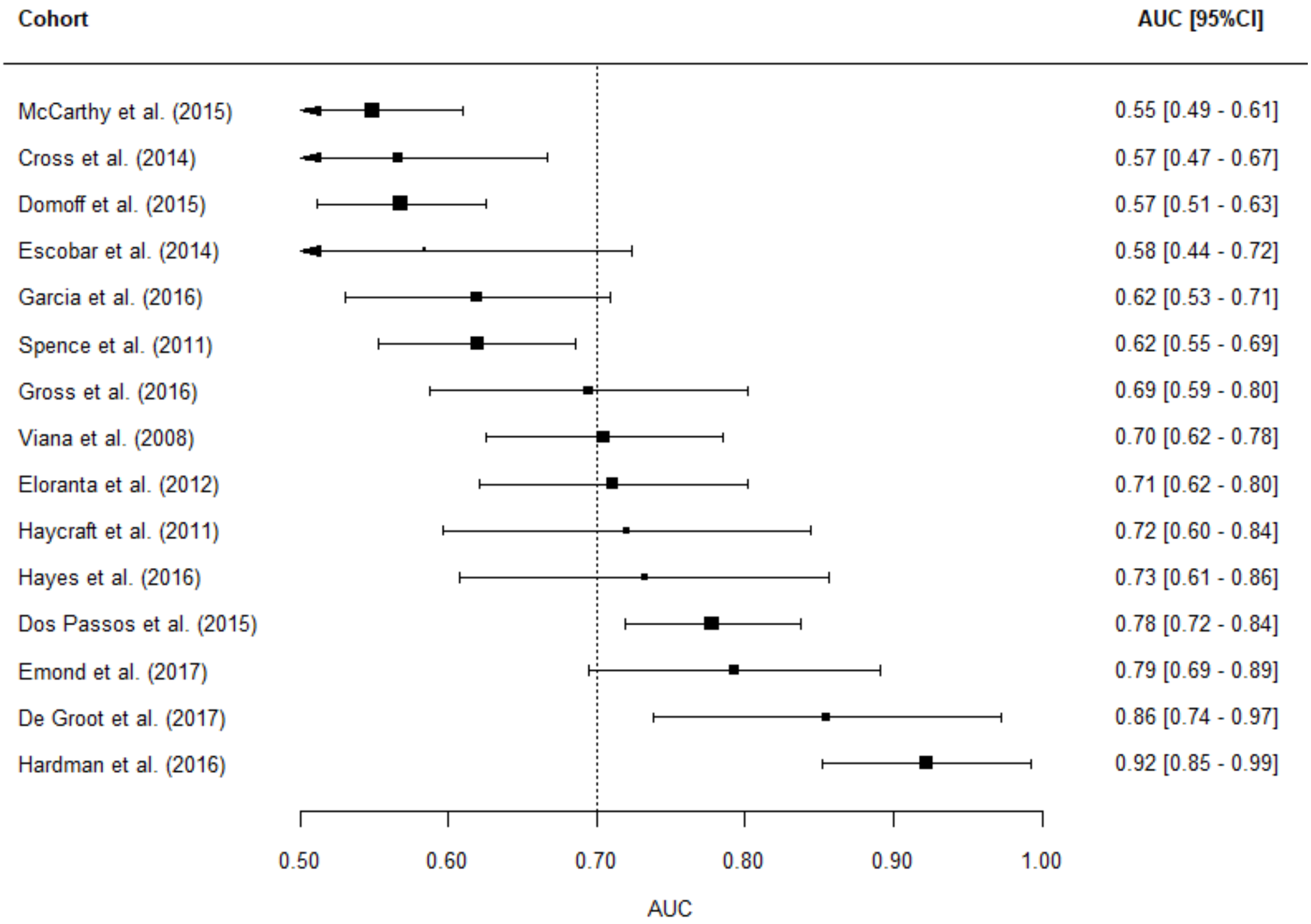

Figure SM 14-3 Forest Plot of Enjoyment of Food (EF) Scores across Cohorts

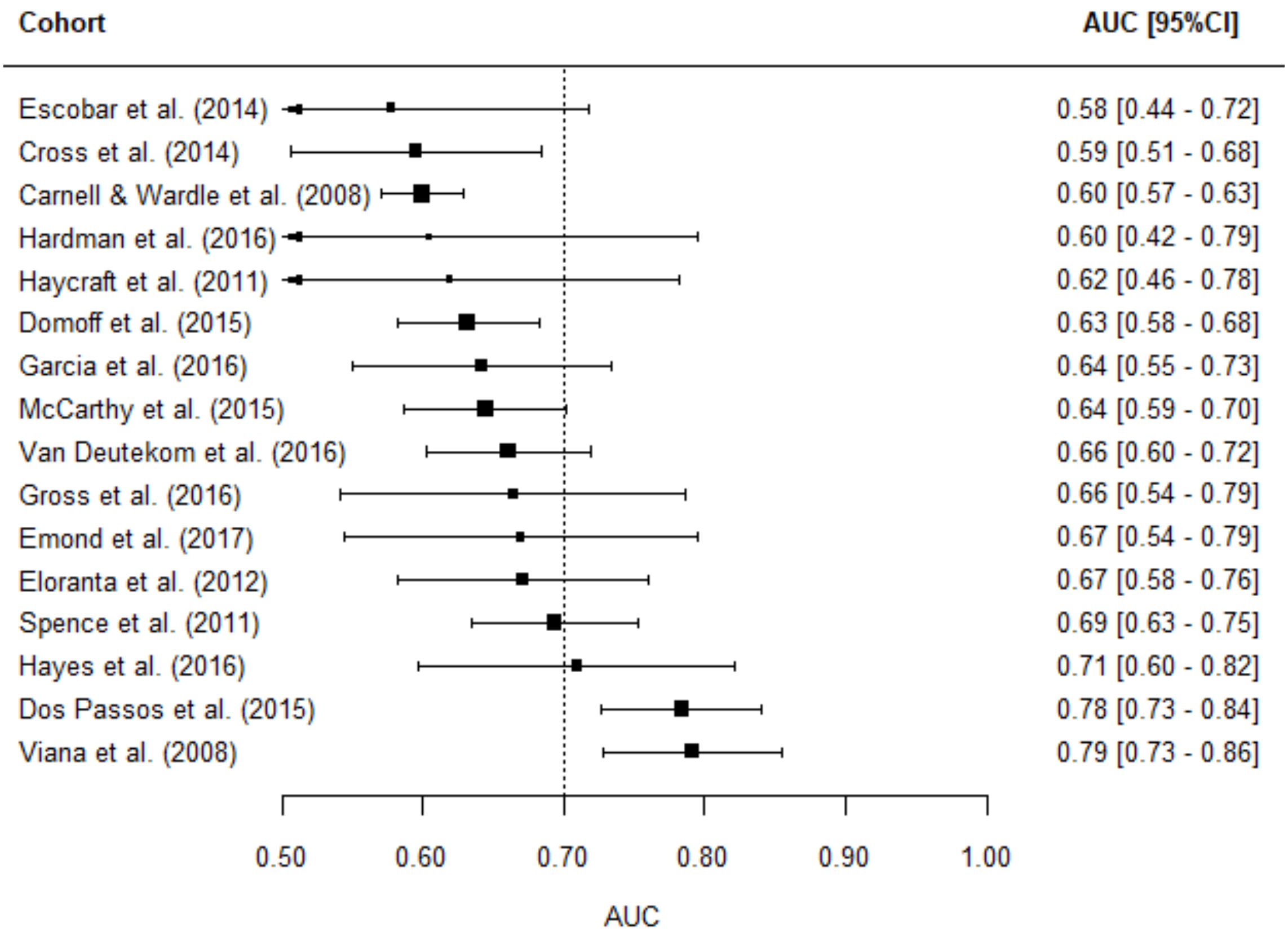

Figure SM 14-4 Forest Plot of Desire to Drink (DD) Scores across Cohorts

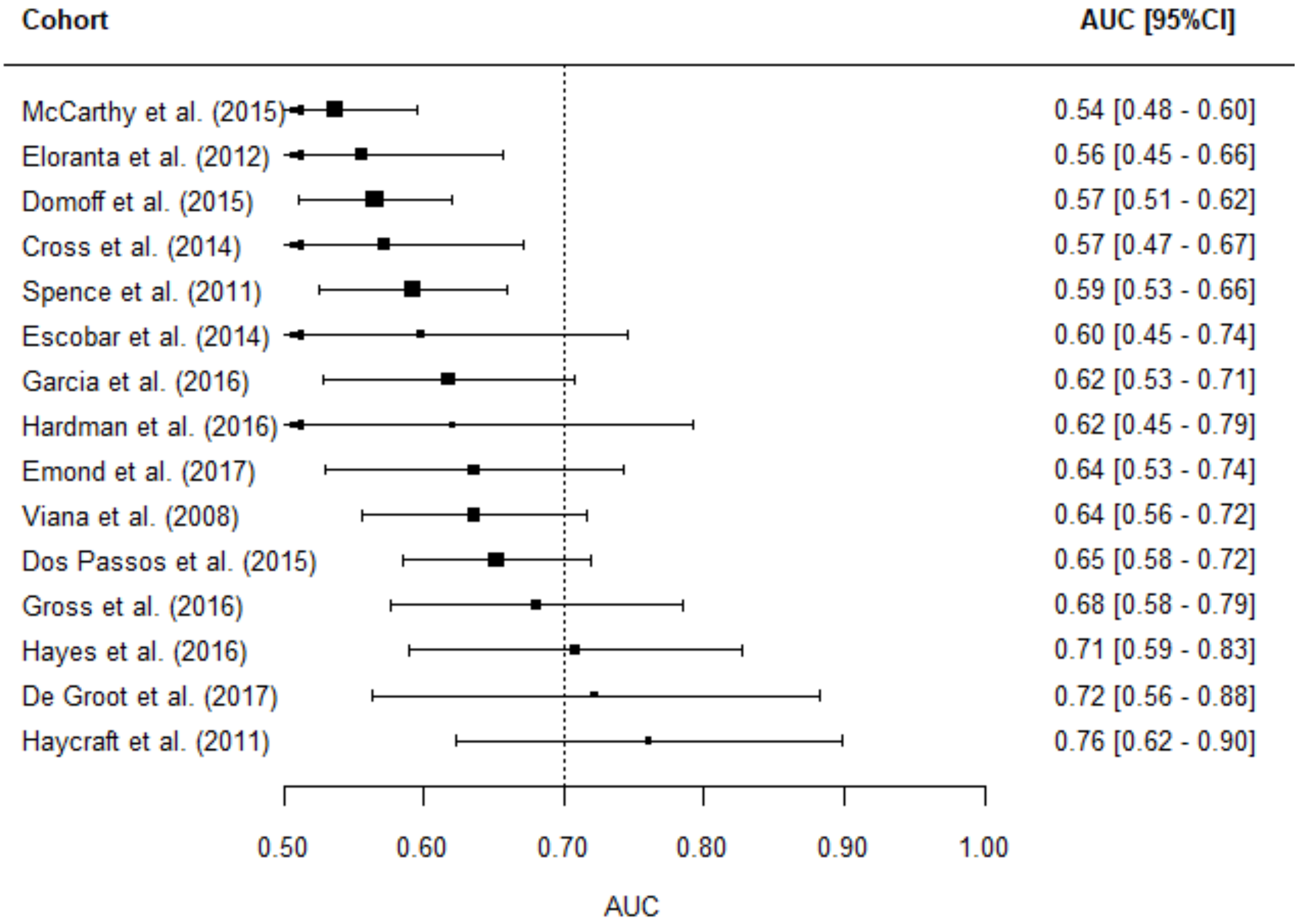

Figure SM 14-5 Forest Plot of Satiety Responsiveness (SR) Scores across Cohorts

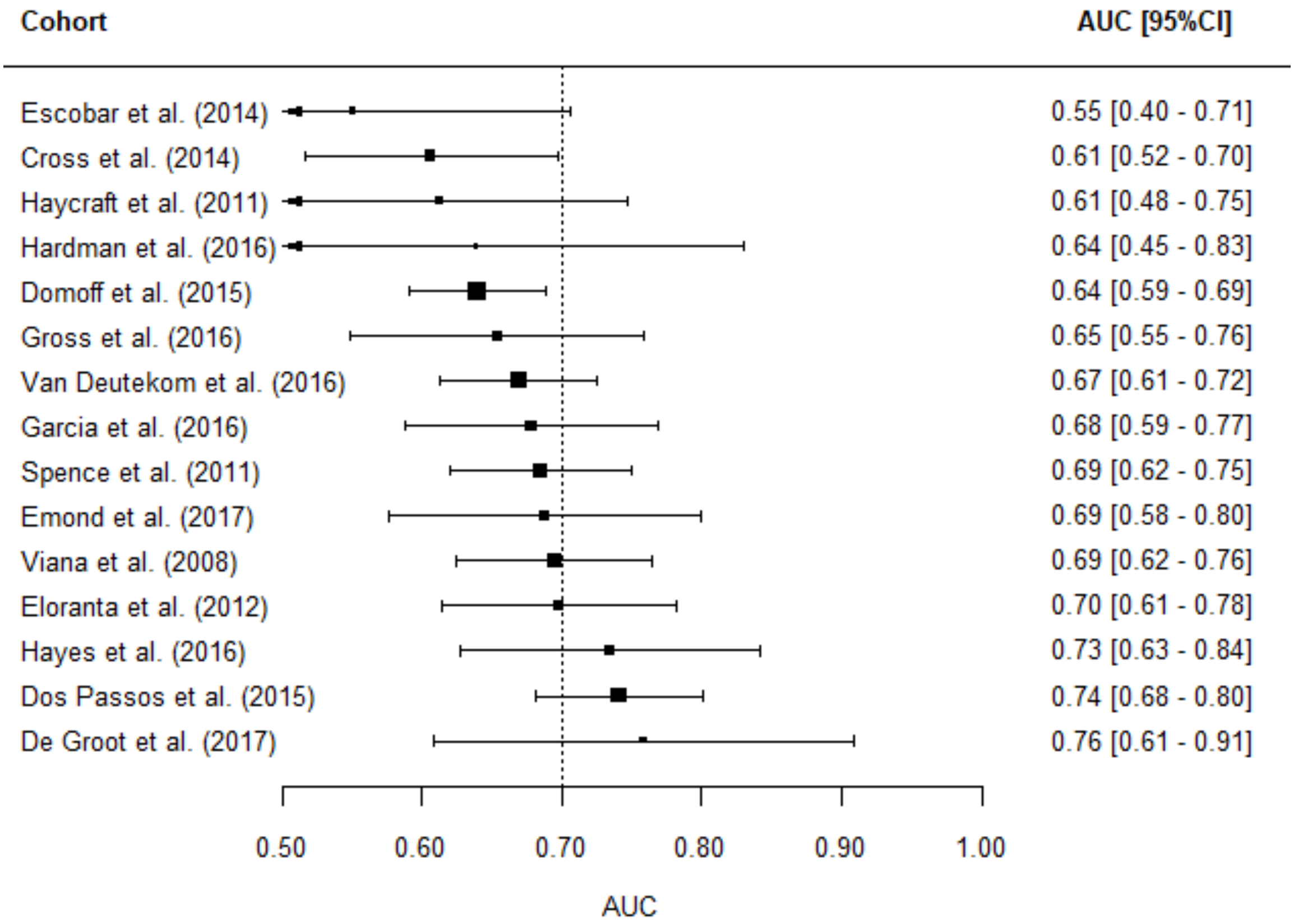

Figure SM 14-6 Forest Plot of Slowness in Eating (SE) Scores across Cohorts

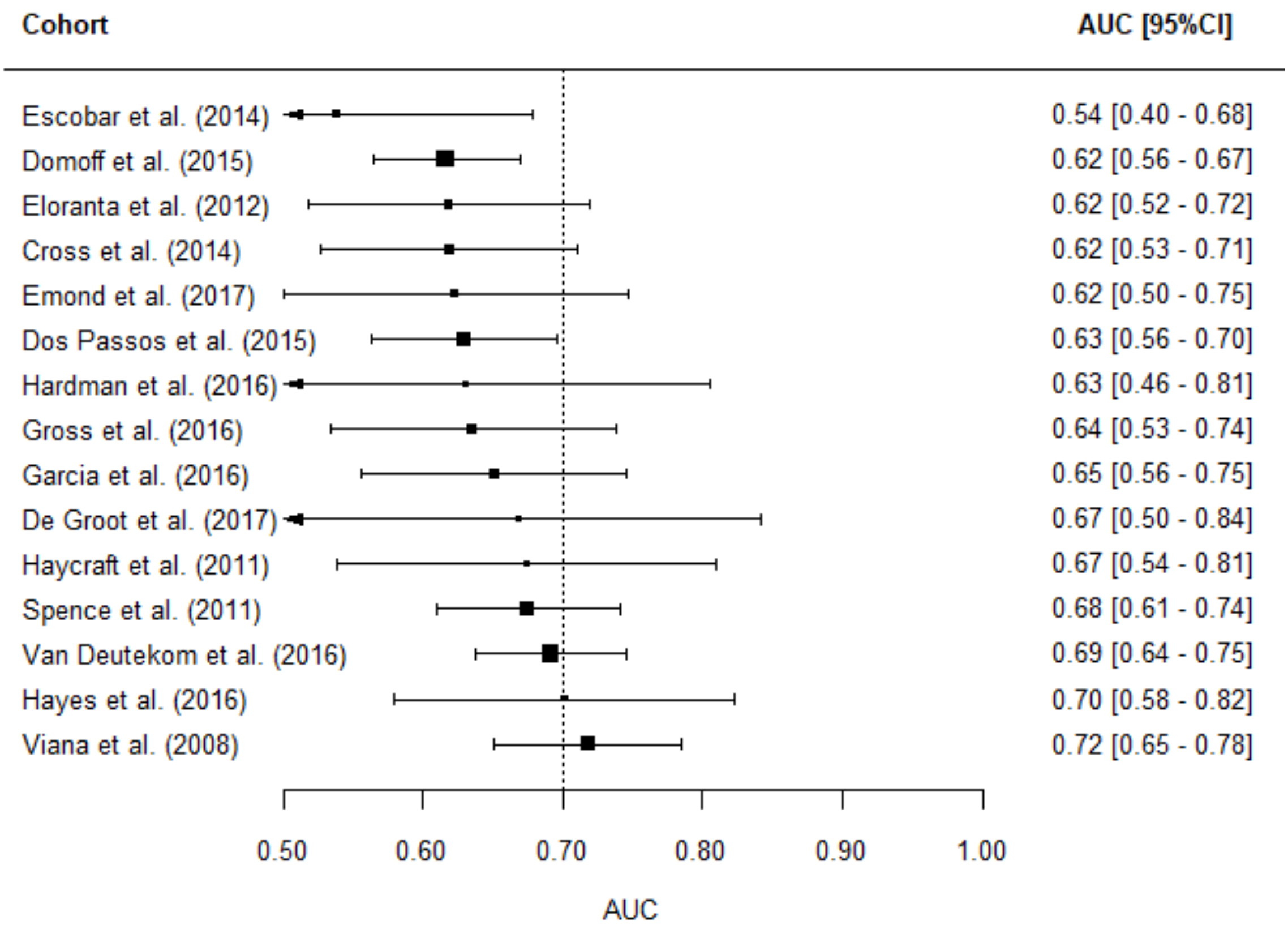

Figure SM 14-7 Forest Plot of Food Fussiness (FF) Scores across Cohorts

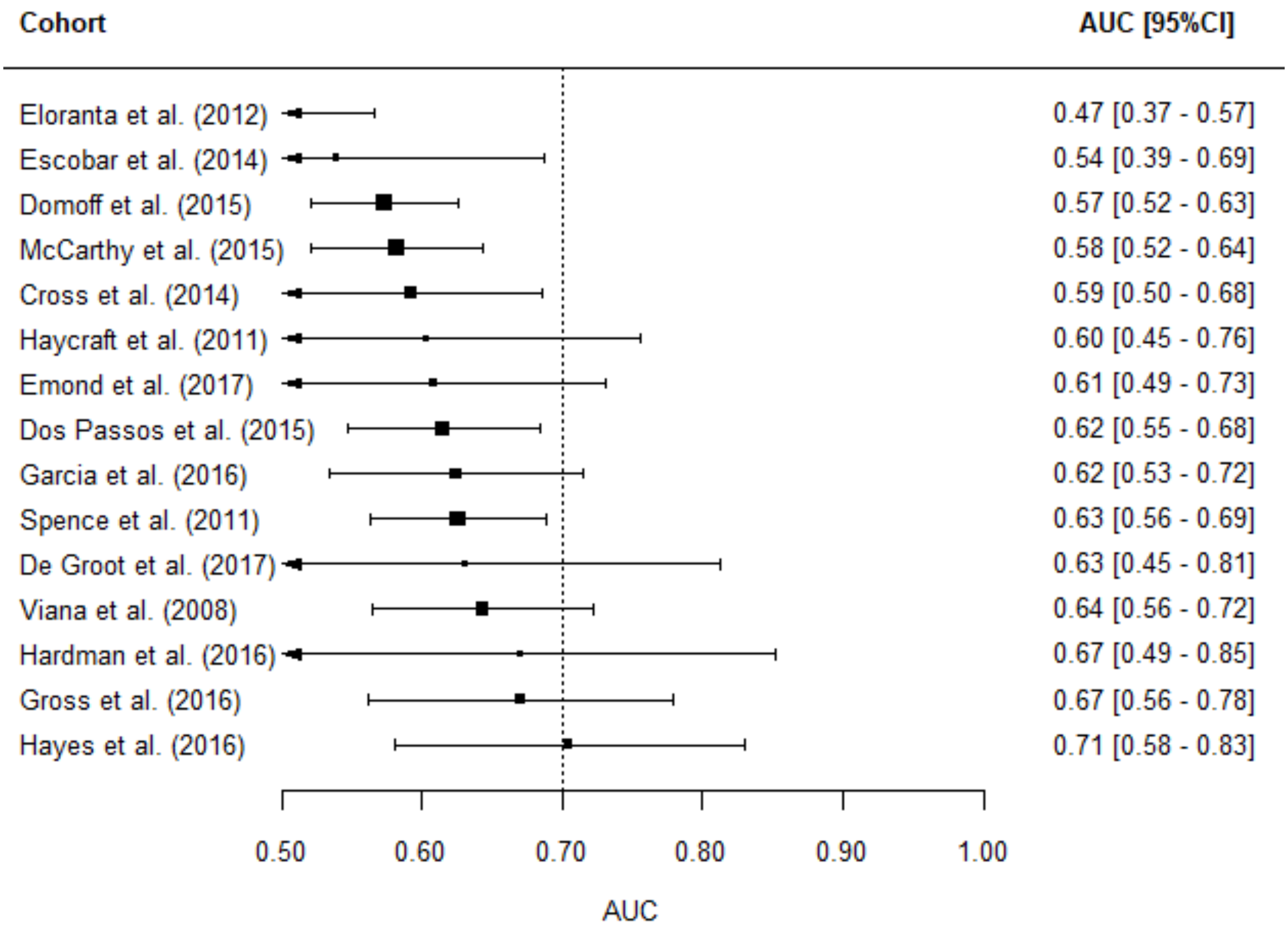

Figure SM 14-8 Forest Plot of Emotional Undereating (EUE) Scores across Cohorts

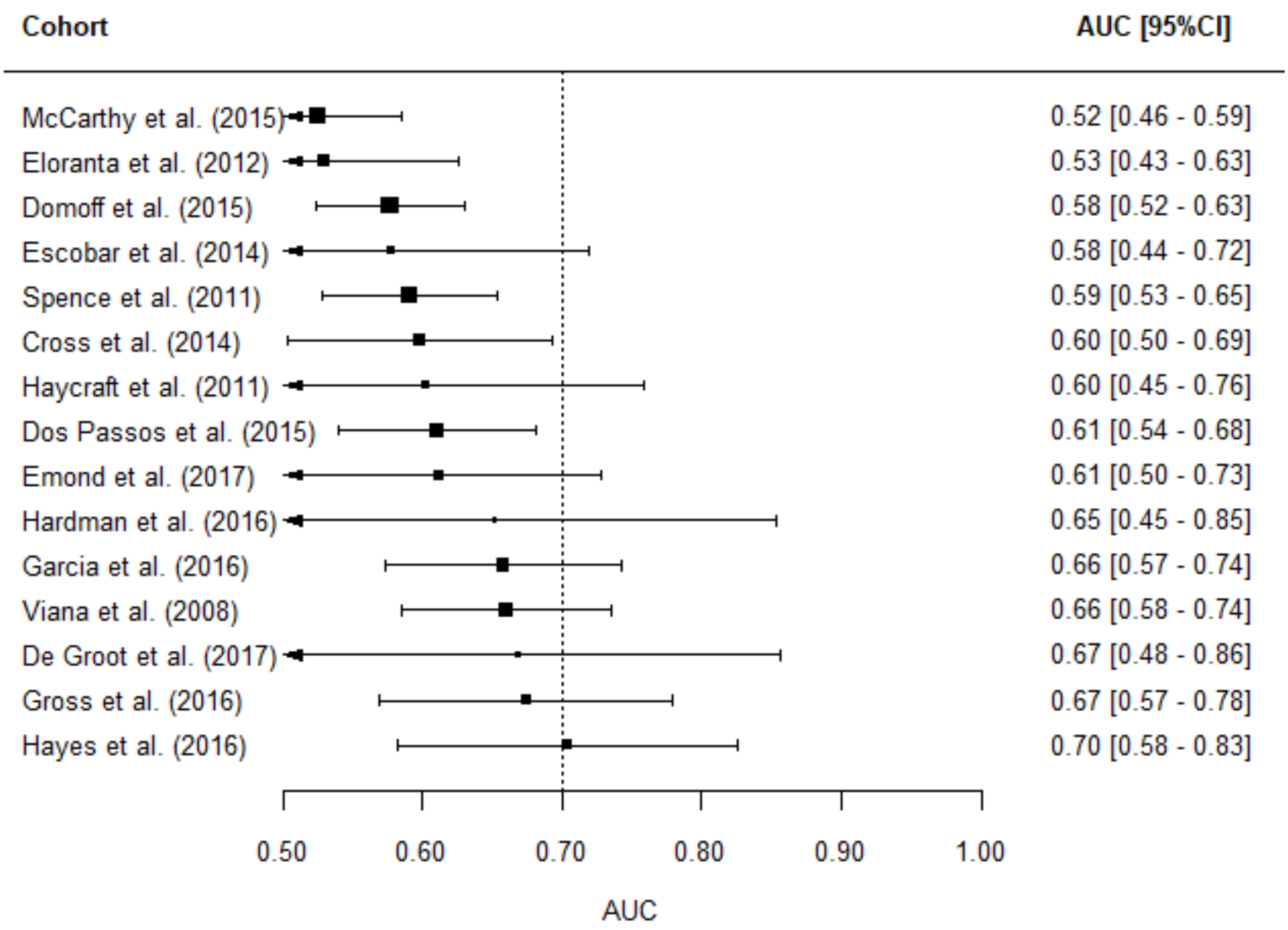

15.0 Meta-regression exploring impact of pre-defined study characteristics on AUCs

Table SM 15-1: Heterogeneity estimates from meta-regression models testing the impact of predefined study characteristics on area under the curve estimates

CEBQ subscale	Tau^2	I^2	H^2	R^2
FR	0.000	0.000	1.000	100.000
EOE	0.005	72.542	3.642	52.065
EF	0.001	37.234	1.593	69.832
DD	0.000	0.000	1.000	100.000
SR	0.000	0.000	1.000	100.000
SE	0.000	0.000	1.000	100.000
FF	0.001	19.378	1.240	0.000
EUE	0.000	0.000	1.000	100.000

Table SM 15-2: Meta-regression models testing the impact of predefined study characteristics on area under the curve (AUC) estimates

CEBQ subscale	Study Characteristic	Estimate (95%CI)	SE	p-value
FR	Intercept	0.796 (0.686, 0.905)	0.056	0.000
	Risk of Bias [a]	0.097 (0.037, 0.157)	0.030	0.001
	Sample Type (Clinical) [b]	-0.083 (-0.214, 0.048)	0.067	0.213
	Sample Type (Community) [b]	-0.014 (-0.09, 0.062)	0.039	0.719
	Language [c]	0.177 (0.13, 0.224)	0.024	0.000
	Standard Deviation	-0.21 (-0.385, -0.035)	0.089	0.018
	Proportion Obesity	0.128 (-0.009, 0.264)	0.070	0.067
EOE	Intercept	0.585 (0.295, 0.876)	0.148	0.000
	Risk of Bias	0.145 (0.036, 0.254)	0.056	0.009
	Sample Type (Clinical) [b]	-0.089 (-0.329, 0.151)	0.123	0.468
	Sample Type (Community) [b]	0.057 (-0.088, 0.202)	0.074	0.440
	Language [c]	0.087 (-0.039, 0.213)	0.064	0.175
	Standard Deviation	-0.071 (-0.592, 0.45)	0.266	0.790
	Proportion Obesity	0.204 (-0.14, 0.547)	0.175	0.245
EF	Intercept	0.668 (0.461, 0.874)	0.105	0.000
	Risk of Bias	-0.013 (-0.102, 0.075)	0.045	0.766
	Sample Type (Clinical) [b]	0.102 (-0.098, 0.303)	0.102	0.318
	Sample Type (Community) [b]	0.027 (-0.043, 0.097)	0.036	0.450
	Language [c]	0.103 (0.024, 0.182)	0.040	0.010
	Standard Deviation	-0.06 (-0.353, 0.233)	0.149	0.687
	Proportion Obesity	-0.031 (-0.254, 0.192)	0.114	0.784
DD	Intercept	0.528 (0.282, 0.774)	0.125	0.000
	Risk of Bias	0.057 (-0.013, 0.126)	0.035	0.108
	Sample Type (Clinical) [b]	-0.025 (-0.159, 0.109)	0.068	0.714
	Sample Type (Community) [b]	0.032 (-0.034, 0.098)	0.034	0.345
	Language [c]	0.024 (-0.032, 0.08)	0.028	0.397
	Standard Deviation	0.005 (-0.235, 0.245)	0.122	0.965
	Proportion Obesity	0.161 (0.005, 0.316)	0.079	0.043
SR	Intercept	0.765 (0.537, 0.993)	0.116	0.000
	Risk of Bias	-0.015 (-0.079, 0.049)	0.033	0.647
	Sample Type (Clinical) [b]	-0.039 (-0.169, 0.09)	0.066	0.552
	Sample Type (Community) [b]	0.016 (-0.065, 0.097)	0.041	0.692
	Language [c]	0.086 (0.024, 0.147)	0.031	0.006
	Standard Deviation	-0.218 (-0.65, 0.213)	0.220	0.321
	Proportion Obesity	0.115 (-0.042, 0.271)	0.080	0.150
SE	Intercept	0.915 (0.644, 1.185)	0.138	0.000
	Risk of Bias	0.016 (-0.054, 0.085)	0.036	0.660
	Sample Type (Clinical) [b]	0.072 (-0.061, 0.205)	0.068	0.289
	Sample Type (Community) [b]	0.024 (-0.061, 0.109)	0.043	0.577
	Language [c]	0.013 (-0.044, 0.07)	0.029	0.651
	Standard Deviation	-0.399 (-0.839, 0.041)	0.224	0.076
	Proportion Obesity	-0.035 (-0.188, 0.119)	0.078	0.657
FF	Intercept	0.447 (0.037, 0.857)	0.209	0.032
	Risk of Bias	0.015 (-0.061, 0.092)	0.039	0.695
	Sample Type (Clinical) [b]	0.06 (-0.101, 0.221)	0.082	0.466
	Sample Type (Community) [b]	0.016 (-0.068, 0.1)	0.043	0.710
	Language [c]	-0.03 (-0.096, 0.037)	0.034	0.378

CEBQ subscale	Study Characteristic	Estimate (95%CI)	SE	p-value
	Standard Deviation	0.15 (-0.353, 0.654)	0.257	0.559
	Proportion Obesity	0.043 (-0.162, 0.247)	0.104	0.683
EUE	Intercept	0.653 (0.122, 1.183)	0.271	0.016
	Risk of Bias	0.022 (-0.05, 0.095)	0.037	0.545
	Sample Type (Clinical) [b]	0.075 (-0.068, 0.218)	0.073	0.302
	Sample Type (Community) [b]	0.048 (-0.027, 0.123)	0.038	0.209
	Language [c]	-0.005 (-0.063, 0.053)	0.029	0.869
	Standard Deviation	-0.135 (-0.7, 0.43)	0.288	0.639
	Proportion Obesity	0.063 (-0.115, 0.241)	0.091	0.486

[a] ref. = Low
[b] ref. = Birth Cohort Sample
[c] ref. = English Language

15.1 Subgroup analyses

Table SM 15-3: Meta-analysis of AUC results for cohorts administering the CEBQ in English

CEBQ subscale	AUC (95%CI)	Tau^2	I^2	N Cohorts
FR	0.66 (0.62, 0.70)	0.0019	48.21	11
EF	0.63 (0.61, 0.66)	0.0005	28.04	13
SR	0.65 (0.63, 0.68)	0.0	0.00	11

Abbreviations: *CEBQ*= Child Eating Behaviour Questionnaire, *FR* = Food Responsiveness, *EOE* = Emotional over-eating, *EF* = Enjoyment of Food, *DD* = Desire to Drink, *SR* = Satiety Responsiveness, *SE* = Slowness in Eating, *FF* = Food Fussiness, *EUE* = Emotional under-eating, *AUC* = Area Under the Curve, *CI* = Confidence Interval, *N* = Number of

Table SM 15-4: Meta-analysis of AUC results for cohorts NOT administering the CEBQ in English

CEBQ subscale	AUC (95%CI)	Tau^2	I^2	N Cohorts
FR	0.82 (0.79, 0.85)	0.00	0.51	4
EF	0.75 (0.68, 0.82)	0.0024	65.88	3
SR	0.71 (0.68, 0.75)	0.0	0.00	4

Abbreviations: *CEBQ*= Child Eating Behaviour Questionnaire, *FR* = Food Responsiveness, *EOE* = Emotional over-eating, *EF* = Enjoyment of Food, *DD* = Desire to Drink, *SR* = Satiety Responsiveness, *SE* = Slowness in Eating, *FF* = Food Fussiness, *EUE* = Emotional under-eating, *AUC* = Area Under the Curve, *CI* = Confidence Interval, *N* = Number of

Table SM 15-5: Meta-analysis of AUC results for cohorts classified as LOW risk of bias

CEBQ subscale	AUC (95%CI)	Tau^2	I^2	N Cohorts
FR	0.68 (0.62, 0.74)	0.0074	83.74	9
EOE	0.63 (0.57, 0.69)	0.0057	78.30	9

Abbreviations: *CEBQ*= Child Eating Behaviour Questionnaire, *FR* = Food Responsiveness, *EOE* = Emotional over-eating, *EF* = Enjoyment of Food, *DD* = Desire to Drink, *SR* = Satiety Responsiveness, *SE* = Slowness in Eating, *FF* = Food Fussiness, *EUE* = Emotional under-eating, *AUC* = Area Under the Curve, *CI* = Confidence Interval, *N* = Number of

Table SM 15-6: Meta-analysis of AUC results for cohorts classified as SOME risk of bias

CEBQ subscale	AUC (95%CI)	Tau^2	I^2	N Cohorts
FR	0.78 (0.72, 0.83)	0.0017	36.83	6
EOE	0.78 (0.70, 0.86)	0.007	74.82	6

Abbreviations: *CEBQ*= Child Eating Behaviour Questionnaire, *FR* = Food Responsiveness, *EOE* = Emotional over-eating, *EF* = Enjoyment of Food, *DD* = Desire to Drink, *SR* = Satiety Responsiveness, *SE* = Slowness in Eating, *FF* = Food Fussiness, *EUE* = Emotional under-eating, *AUC* = Area Under the Curve, *CI* = Confidence Interval, *N* = Number of

16.0 Shape of association between CEBQ subscale scores and BMI-SDS groups

Figure SM 16-1 Shape of Association of Food Responsiveness (FR) and BMI-SDS Group in stratified sex- and age- groups

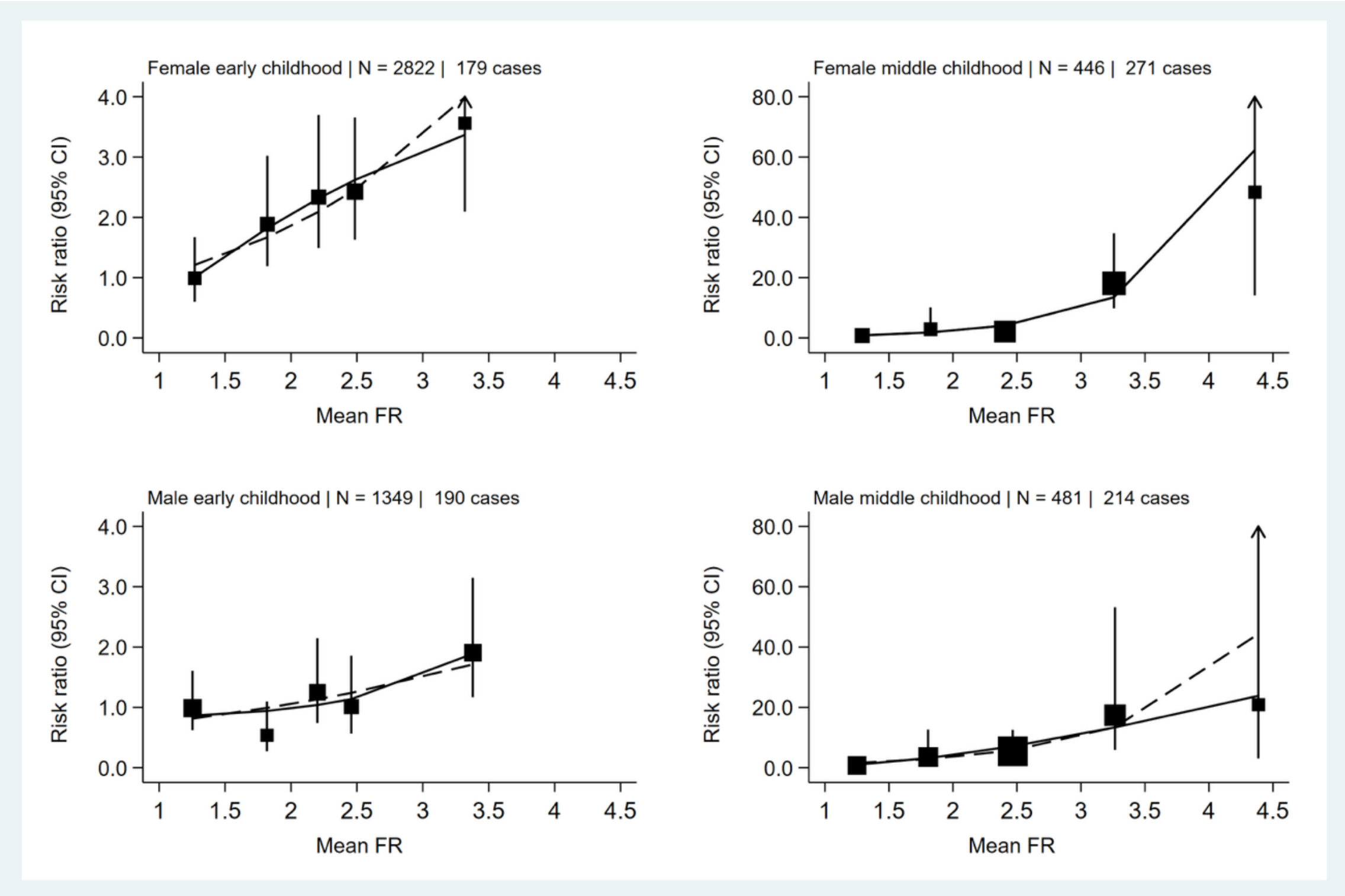

Figure SM 16-2 Shape of Association of Emotional Overeating (EOE) and BMI-SDS Group in stratified sex- and age- groups

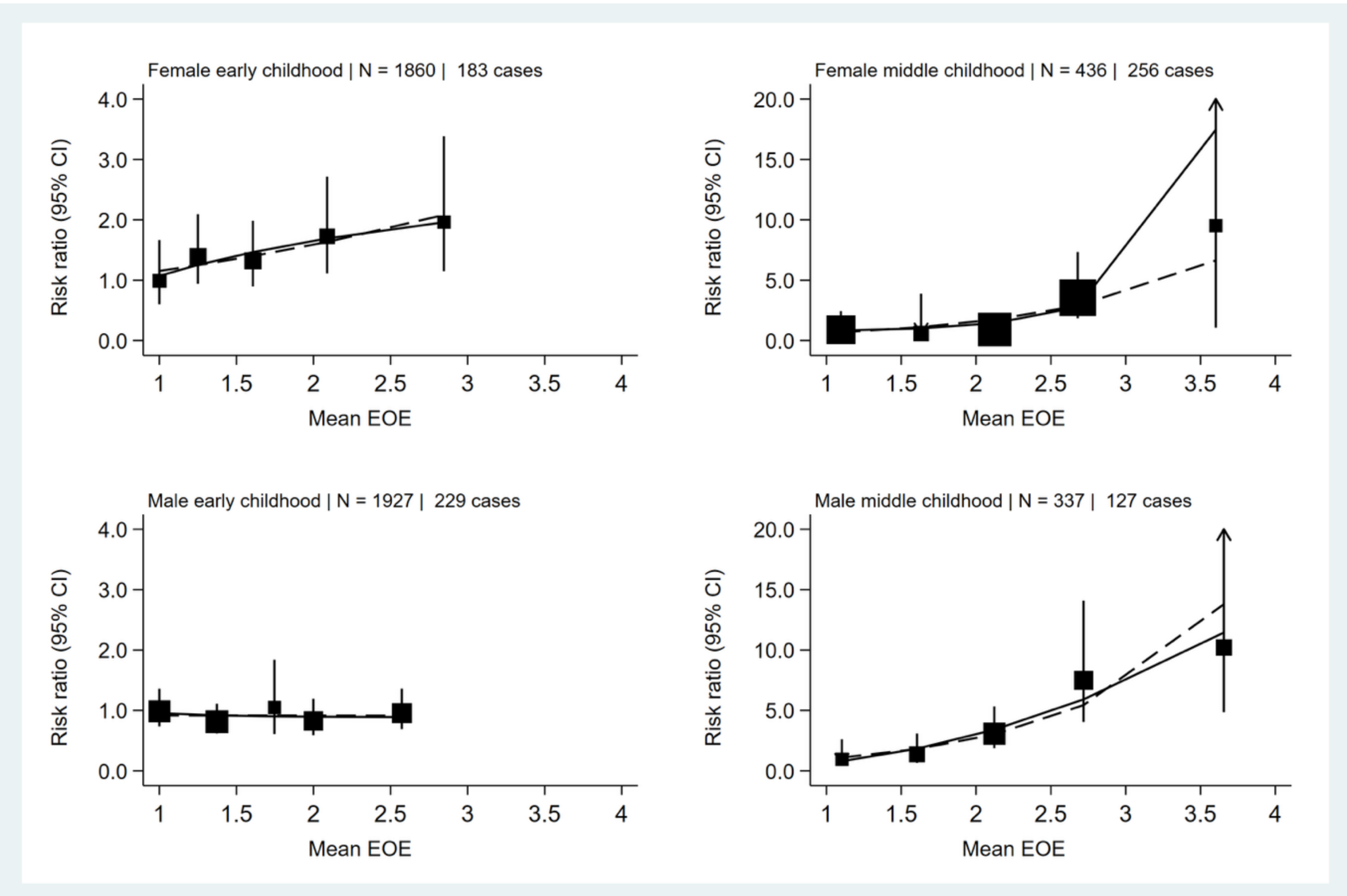

Figure SM 16-3 Shape of Association of Enjoyment of Food (EF) and BMI-SDS Group in stratified sex- and age- groups

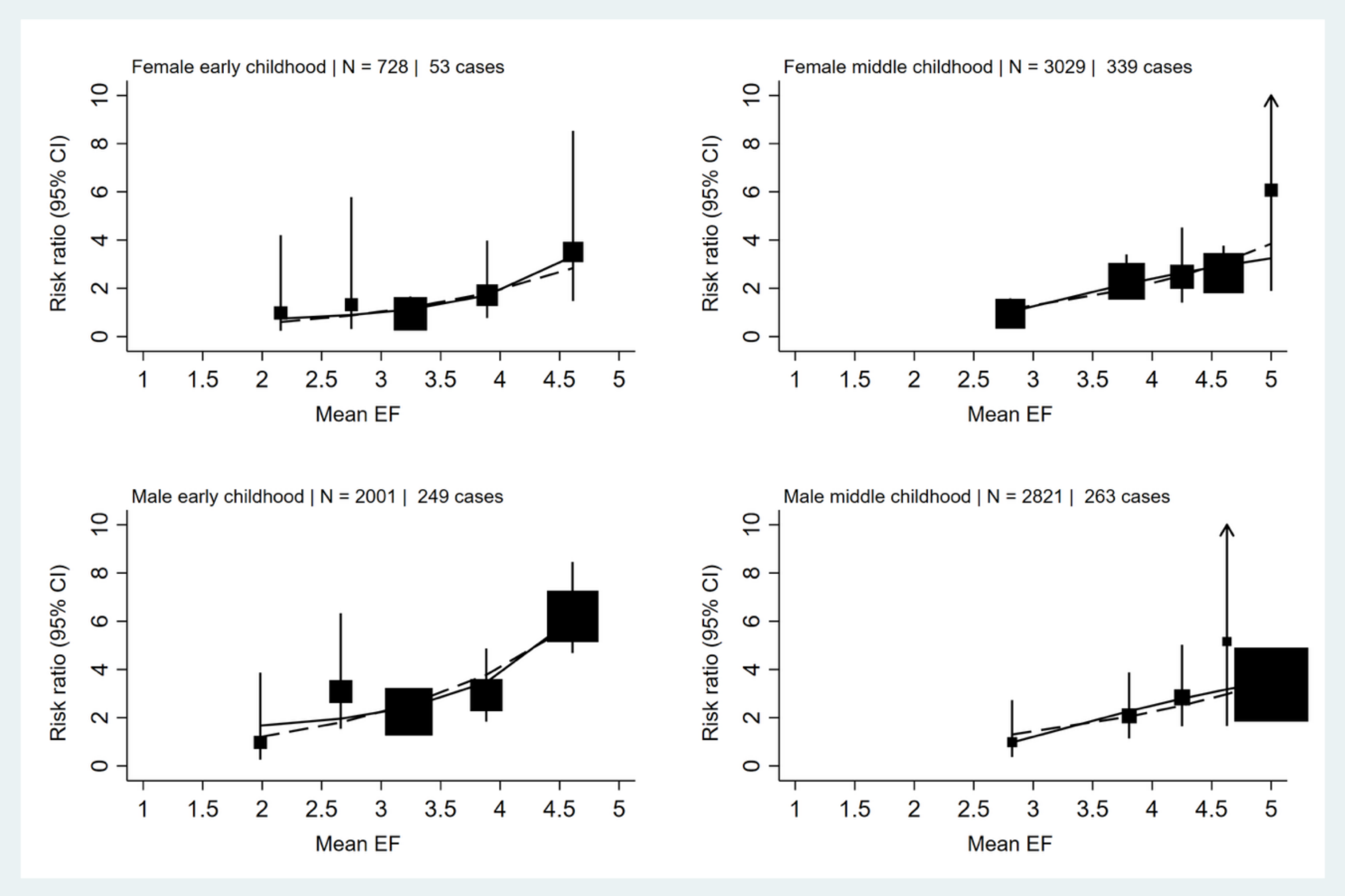

Figure SM 16-4 Shape of Association of Desire to Drink (DD) and BMI-SDS Group in stratified sex- and age- groups

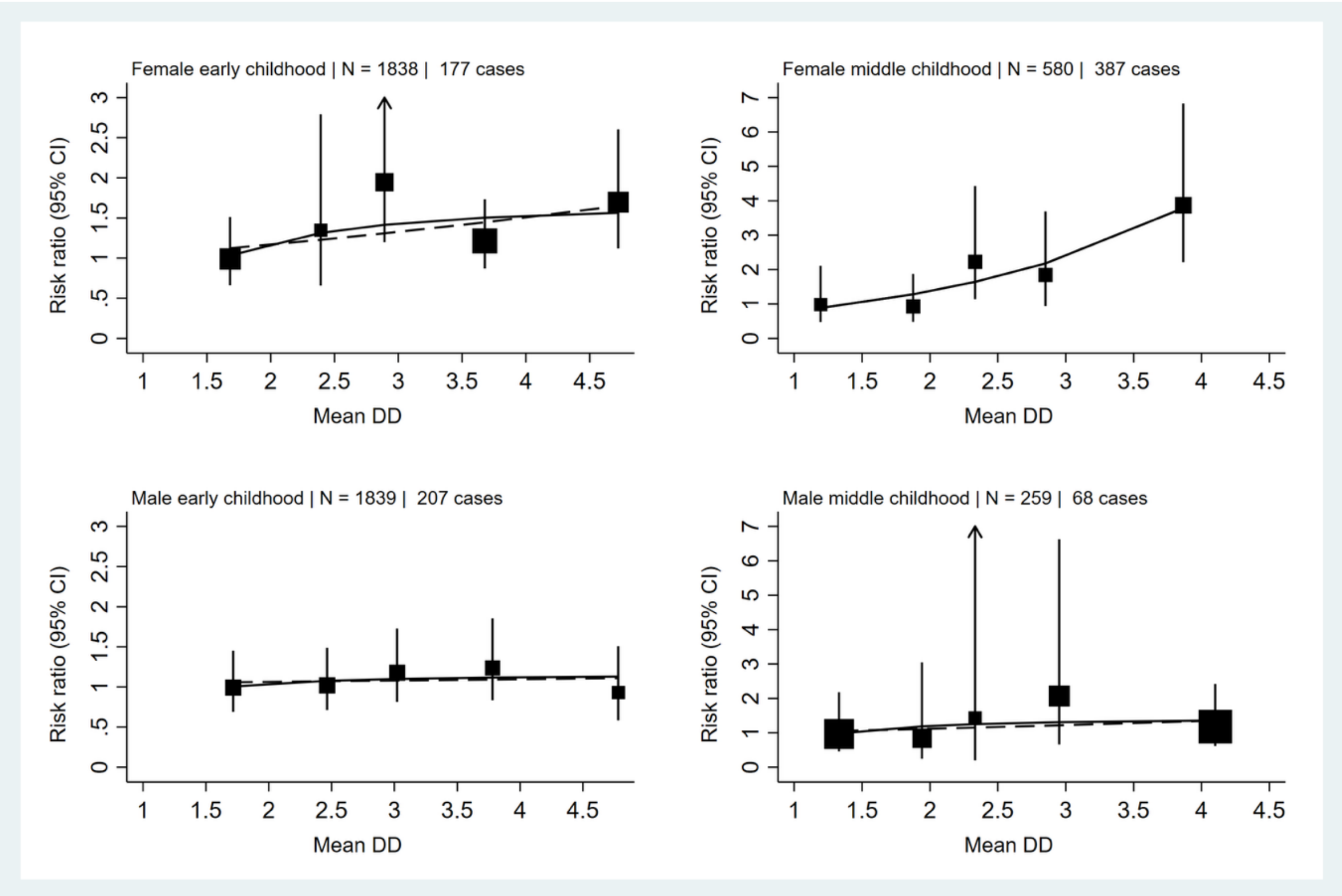

Figure SM 16-5 Shape of Association of Satiety Responsiveness (SR) and BMI-SDS Group in stratified sex- and age- groups

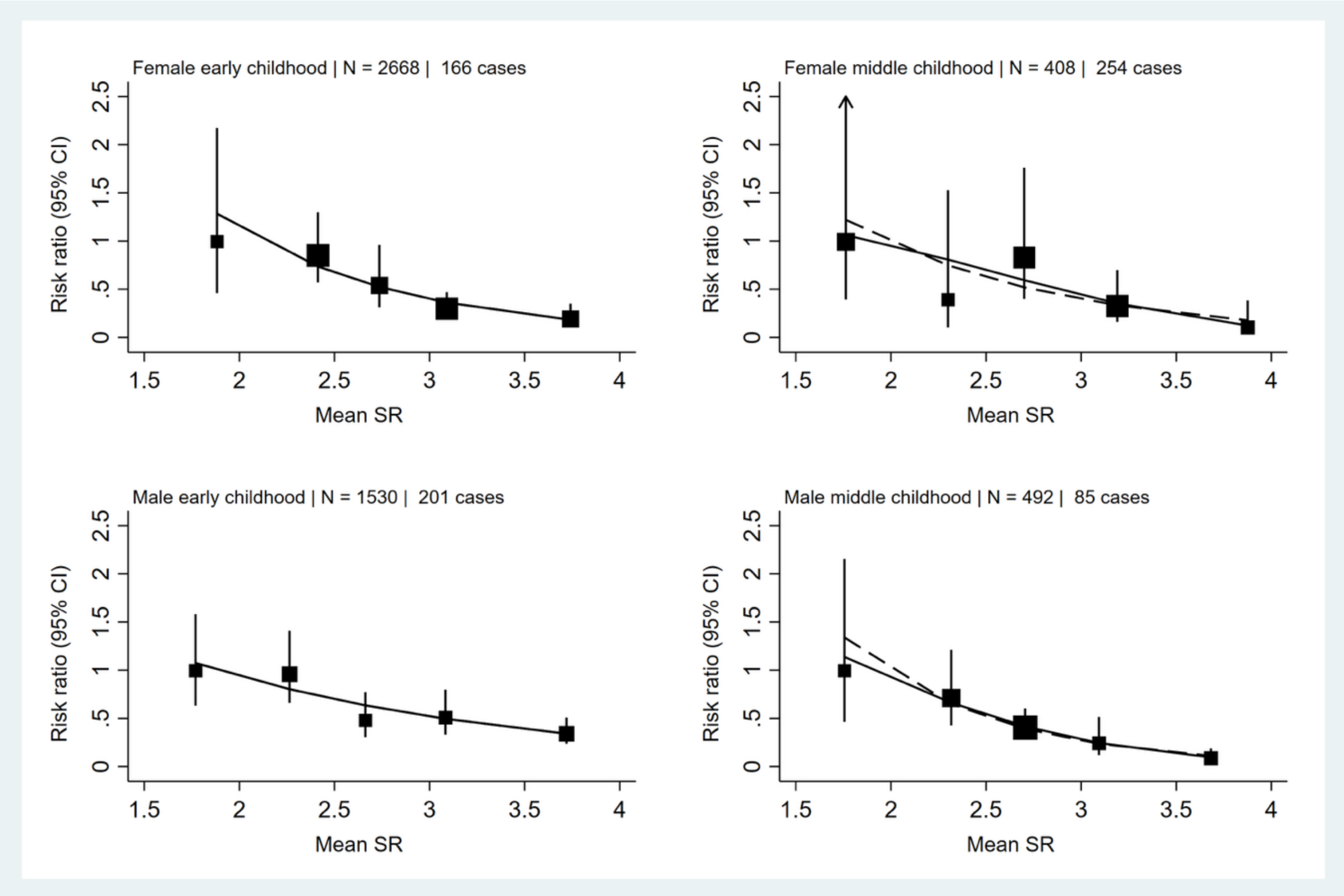

Figure SM 16-6 Shape of Association of Slowness in Eating (SE) and BMI-SDS Group in stratified sex- and age- groups

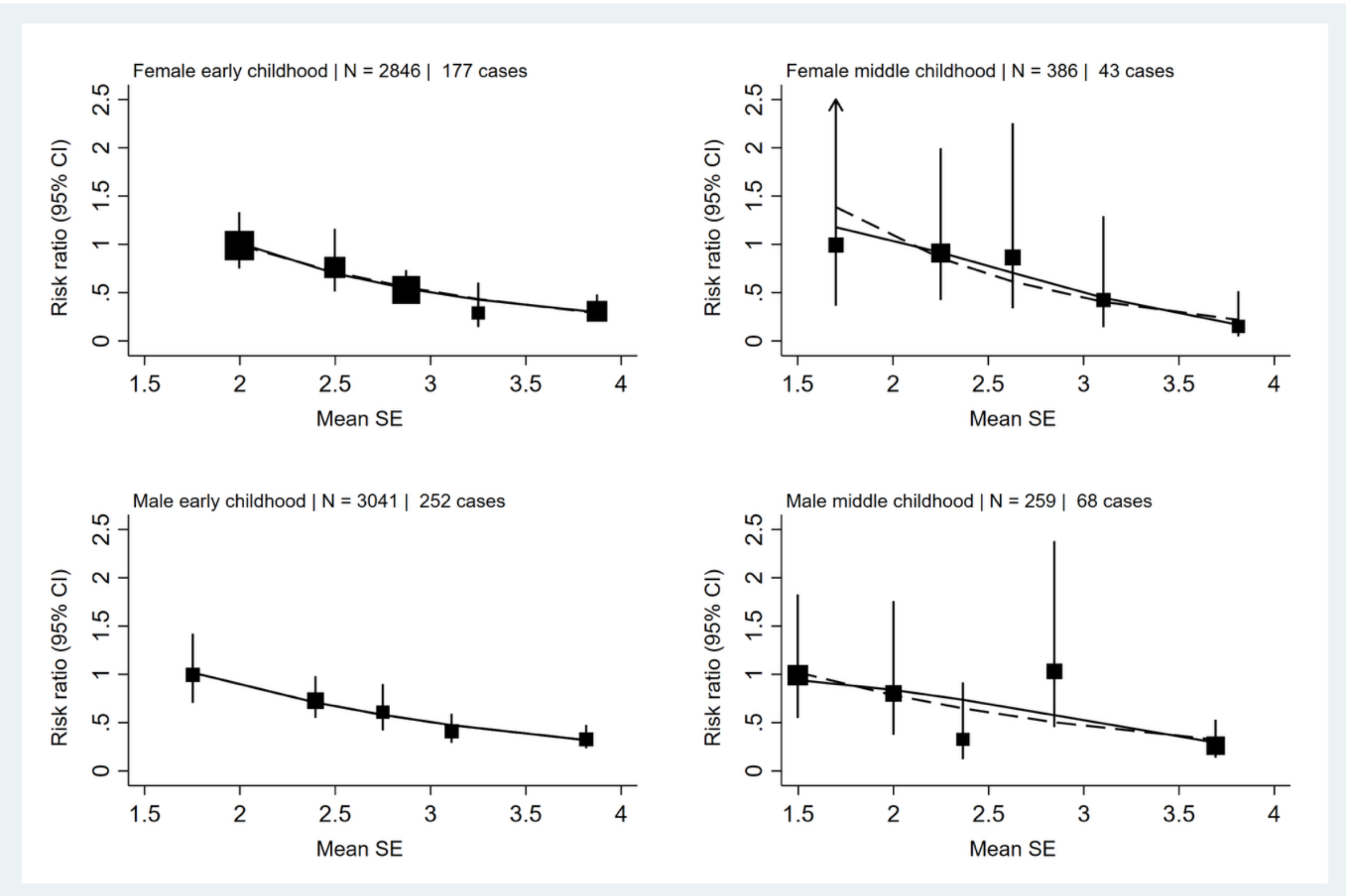

Figure SM 16-7 Shape of Association of Food Fussiness (FF) and BMI-SDS Group in stratified sex- and age- groups

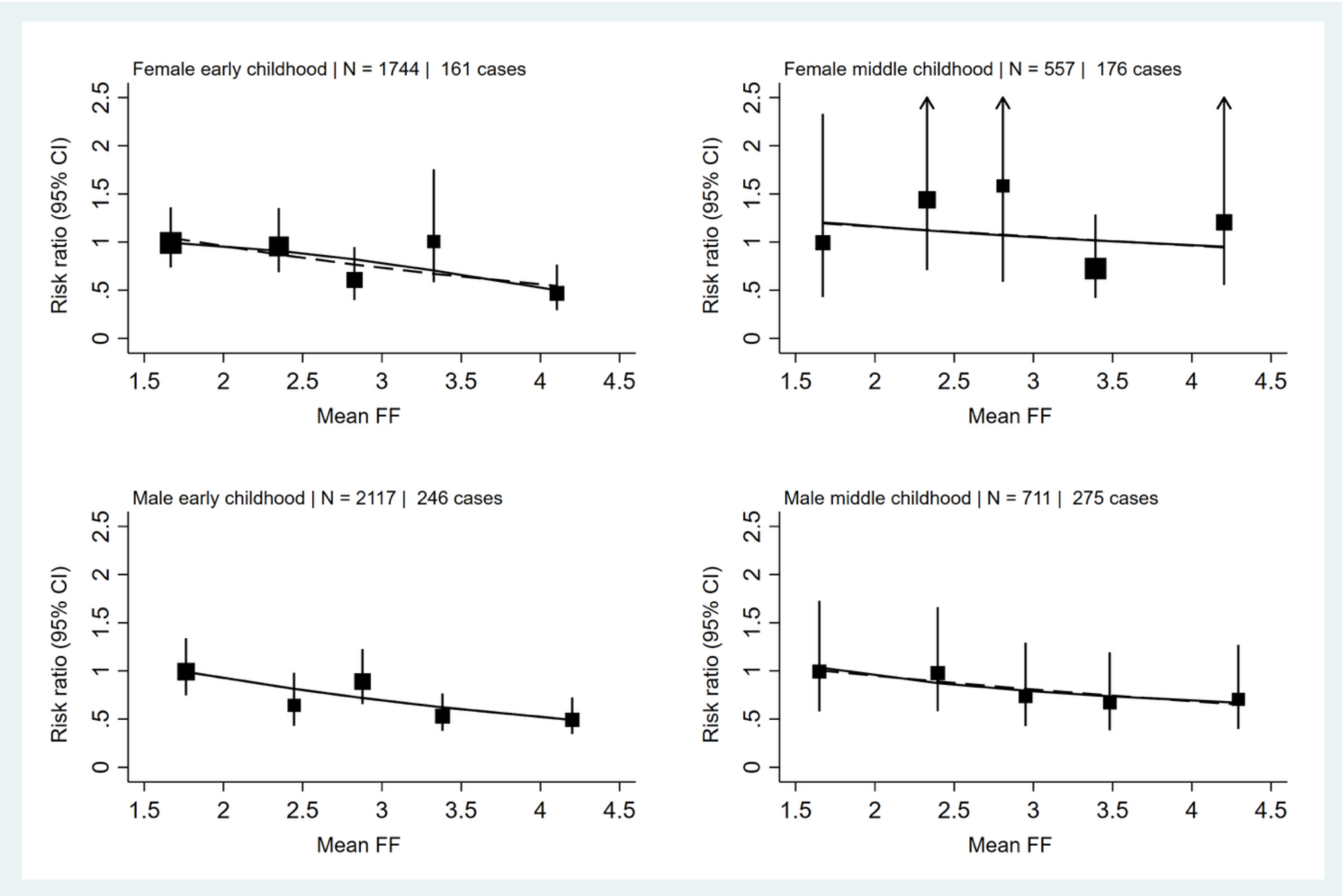

Figure SM 16-8 Shape of Association of Emotional Undereating (EUE) and BMI-SDS Group in stratified sex- and age- groups

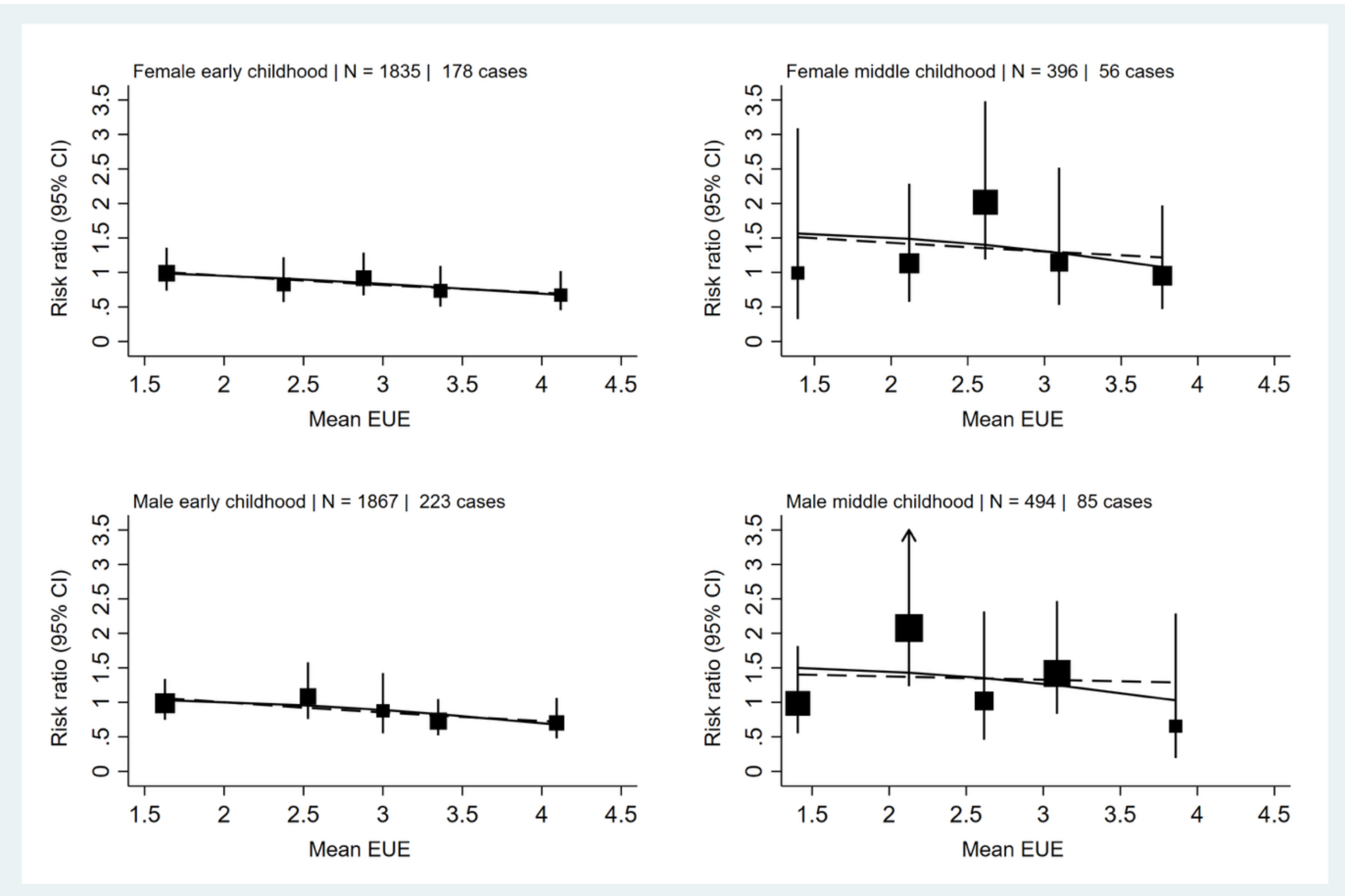

17.0 Cut-off scores derived with a fixed sensitivity level of at least 60%

Table SM 17-1: Age- and sex-specific cut-off scores to identify weight status (no obesity a vs obesity b) as derived using a minimum sensitivity level of 60%

	Early childhood[c]					Middle childhood[d]				
	**N**	**AUC [95%CI]**	**Cut-off[e]**	**Se [95%CI]**	**Sp [95%CI]**	**N**	**AUC [95%CI]**	**Cut-off[e]**	**Se [95%CI]**	**Sp [95%CI]**
**FR**										
Males	3893	0.68 [0.64, 0.72]	1.60	0.86 [0.81, 0.89]	0.24 [0.22, 0.26]	3556	0.87 [0.84, 0.89]	2.20	0.86 [0.83, 0.90]	0.68 [0.64, 0.73]
Females	3817	0.70 [0.66, 0.73]	1.80	0.85 [0.81, 0.89]	0.37 [0.35, 0.39]	3760	0.89 [0.86, 0.91]	2.40	0.85 [0.81, 0.88]	0.78 [0.74, 0.82]
**EF**										
Males	3893	0.69 [0.66, 0.72]	2.75	0.90 [0.86, 0.93]	0.31 [0.30, 0.33]	3556	0.57 [0.54, 0.59]	3.25	0.90 [0.87, 0.92]	0.18 [0.17, 0.19]
Females	3817	0.71 [0.68, 0.74]	3.00	0.86 [0.82, 0.90]	0.42 [0.41, 0.44]	3760	0.58 [0.55, 0.60]	3.50	0.86 [0.83, 0.89]	0.24 [0.22, 0.25]
**EOE**										
Males	3893	0.55 [0.52, 0.59]	1.00	1 [0.99, NA]	0 [NA, 0]	3556	0.79 [0.76, 0.82]	1.50	0.89 [0.85, 0.92]	0.42 [0.37, 0.47]
Females	3817	0.61 [0.58, 0.65]	1.00	1 [0.99, NA]	0 [NA, 0]	3760	0.79 [0.76, 0.82]	1.50	0.89 [0.85, 0.92]	0.44 [0.40, 0.49]
**DD**										
Females	3817	0.55 [0.52, 0.59]	2.00	0.89 [0.85, 0.93]	0.15 [0.13, 0.16]	3760	0.70 [0.67, 0.74]	1.67	0.89 [0.85, 0.92]	0.28 [0.24, 0.32]
**SR**										
Males	3893	0.54 [0.50, 0.57]	3.20	0.86 [0.81, 0.89]	0.18 [0.17, 0.20]	3556	0.78 [0.75, 0.81]	2.80	0.88 [0.85, 0.91]	0.49 [0.45, 0.54]
Females	3817	0.56 [0.53, 0.60]	3.20	0.85 [0.81, 0.89]	0.21 [0.19, 0.22]	3760	0.78 [0.76, 0.81]	2.80	0.87 [0.83, 0.90]	0.54 [0.50, 0.59]
**SE**										
Males	3893	0.56 [0.53, 0.60]	3.25	0.86 [0.82, 0.9]	0.18 [0.16, 0.19]	3556	0.68 [0.65, 0.72]	2.75	0.86 [0.82, 0.89]	0.34 [0.29, 0.38]
Females	3817	0.57 [0.53, 0.61]	3.25	0.85 [0.8, 0.89]	0.20 [0.18, 0.21]	3760	0.72 [0.69, 0.75]	3.00	0.89 [0.86, 0.92]	0.33 [0.29, 0.38]

Abbreviations: *CEBQ*= Child Eating Behaviour Questionnaire, *FR* = Food Responsiveness, *EF* = Enjoyment of Food, *EOE* = Emotional over-eating, *SR* = Satiety Responsiveness, *N* = Number of participants, *AUC* = Area Under the Curve, *CI* = Confidence Interval, *Se* = Sensitivity, *Sp* = Specificity

[a] Classified into the no obesity group if BMI SDS ≤ 2

[b] Classified into the obesity group if BMI SDS > 2

[c] Children aged ≥ 2 to <7 years

[d] Children aged ≥ 7 to <12 years

[e] positive classification follows a score ≤ cut-off for subscales SR and a score ≥ cut-off for subscales FR, EF, and EOE

Table SM 17-2: Random-Effects Meta-Analysis of Cut-Off Score Performance (Minimum Sensitivity 60%) and Heterogeneity Across Cohorts

	Early childhood [a]					Middle childhood [b]				
	**N Cohorts**	**Se [95%CI] [c]**	**I^2**	**Sp [95%CI] [c]**	**I^2**	**N Cohorts**	**Se [95%CI] [c]**	**I^2**	**Sp [95%CI] [c]**	**I^2**
**FR**										
Males	12	0.87 [0.83, 0.91]	0.00	0.19 [0.13, 0.25]	89.22	9	0.84 [0.74, 0.94]	85.77	0.51 [0.31, 0.70]	92.77
Females	12	0.86 [0.82, 0.91]	0.00	0.32 [0.23, 0.41]	94.48	9	0.79 [0.67, 0.90]	83.28	0.61 [0.39, 0.84]	97.08
**EF**										
Males	13	0.91 [0.84, 0.98]	82.84	0.18 [0.05, 0.30]	99.09	10	0.91 [0.87, 0.96]	56.88	0.36 [0.2, 0.51]	95.45
Females	13	0.88 [0.75, 1.00]	92.49	0.23 [0.09, 0.38]	99.21	10	0.85 [0.79, 0.91]	60.29	0.39 [0.18, 0.6]	98.18
**EOE**										
Males	12	1.00 [0.98, 1.02]	0.00	0 [0, 0]	0.00	8	0.92 [0.86, 0.97]	57.70	0.33 [0.21, 0.45]	80.18
Females	12	1.00 [0.98, 1.02]	0.00	0 [0, 0]	0.00	8	0.90 [0.85, 0.96]	43.80	0.33 [0.18, 0.48]	91.26
**DD**										
Females	12	0.91 [0.87, 0.95]	0.00	0.15 [0.09, 0.20]	89.46	8	0.85 [0.75, 0.94]	81.32	0.23 [0.15, 0.30]	66.84
**SR**										
Males	12	0.87 [0.80, 0.93]	62.20	0.26 [0.17, 0.35]	95.94	9	0.87 [0.80, 0.93]	49.09	0.50 [0.45, 0.55]	4.14
Females	12	0.87 [0.82, 0.92]	21.15	0.31 [0.21, 0.41]	96.21	9	0.82 [0.75, 0.90]	61.97	0.41 [0.23, 0.60]	94.37
**SE**										
Males	12	0.87 [0.81, 0.93]	43.20	0.26 [0.18, 0.33]	92.40	9	0.83 [0.76, 0.90]	45.12	0.36 [0.25, 0.47]	74.08
Females	12	0.86 [0.81, 0.91]	14.80	0.28 [0.20, 0.36]	93.37	9	0.90 [0.87, 0.93]	0.00	0.27 [0.17, 0.38]	81.29

Abbreviations: *FR* = Food Responsiveness, *EF* = Enjoyment of Food, *EOE* = Emotional over-eating, *SR* = Satiety Responsiveness, *N* = Number of, *Se* = Sensitivity, *Sp* = Specificity, *CI* = Confidence Interval

[a] Children aged ≥ 2 to <7 years

[b] Children aged ≥ 7 to <12 years

[c] Representing results from random effects meta-analyses after applying pooled-sample cut-offs to each cohort
